# Human Induced Pluripotent Stem Cell-Derived Microglia-Like Cells Harboring TREM2 Missense Mutations Show Specific Deficits in Phagocytosis

**DOI:** 10.1016/j.celrep.2018.07.094

**Published:** 2018-08-28

**Authors:** Pablo Garcia-Reitboeck, Alexandra Phillips, Thomas M. Piers, Claudio Villegas-Llerena, Matt Butler, Anna Mallach, Celia Rodrigues, Charles E. Arber, Amanda Heslegrave, Henrik Zetterberg, Harald Neumann, Stephen Neame, Henry Houlden, John Hardy, Jennifer M. Pocock

**Affiliations:** 1 Department of Neuroinflammation, University College London Institute of Neurology, London WC1N 1PJ, UK; 2 Department of Molecular Neuroscience, University College London Institute of Neurology, London WC1N 1PJ, UK; 3 Eisai:UCL Therapeutic Innovation Group, University College London Institute of Neurology, London WC1N 1PJ, UK; 4 Department of Psychiatry and Neurochemistry, Institute of Neuroscience and Physiology, The Sahlgrenska Academy at the University of Gothenburg, Gothenburg 41345, Sweden; 5 Neural Regeneration Group, Institute of Reconstructive Neurobiology, University of Bonn, Bonn 53127, Germany; 6 Neurology Business Group, Hatfield Research Laboratories, Neurology Innovation Centre, Eisai Limited, Hatfield, AL10 9SN, UK

**Keywords:** induced pluripotent stem cells, microglia, TREM2, phagocytosis, cytokine, Nasu-Hakola disease, Alzheimer disease

## Abstract

Dysfunction of microglia, the brain’s immune cells, is linked to neurodegeneration. Homozygous missense mutations in *TREM2* cause Nasu-Hakola disease (NHD), an early-onset dementia. To study the consequences of these *TREM2* variants, we generated induced pluripotent stem cell-derived microglia-like cells (iPSC-MGLCs) from patients with NHD caused by homozygous T66M or W50C missense mutations. iPSC-MGLCs expressed microglial markers and secreted higher levels of TREM2 than primary macrophages. TREM2 expression and secretion were reduced in variant lines. LPS-mediated cytokine secretion was comparable between control and TREM2 variant iPSC-MGLCs, whereas survival was markedly reduced in cells harboring missense mutations when compared with controls. Furthermore, TREM2 missense mutations caused a marked impairment in the phagocytosis of apoptotic bodies, but not in *Escherichia coli* or zymosan substrates. Coupled with changes in apoptotic cell-induced cytokine release and migration, these data identify specific deficits in the ability of iPSC-MGLCs harboring *TREM2* missense mutations to respond to specific pathogenic signals.

## Highlights

• Generated human microglia-like cells from *TREM2* T66M and W50C mutation carriers
• Heterozygous and homozygous *TREM2* variants impair shedding of soluble TREM2
• Cytokine secretion not altered in *TREM2* variants following LPS exposure
• Substrate specific impairment of microglial function observed in *TREM2* variants

## Graphical Abstract

**Figure:**
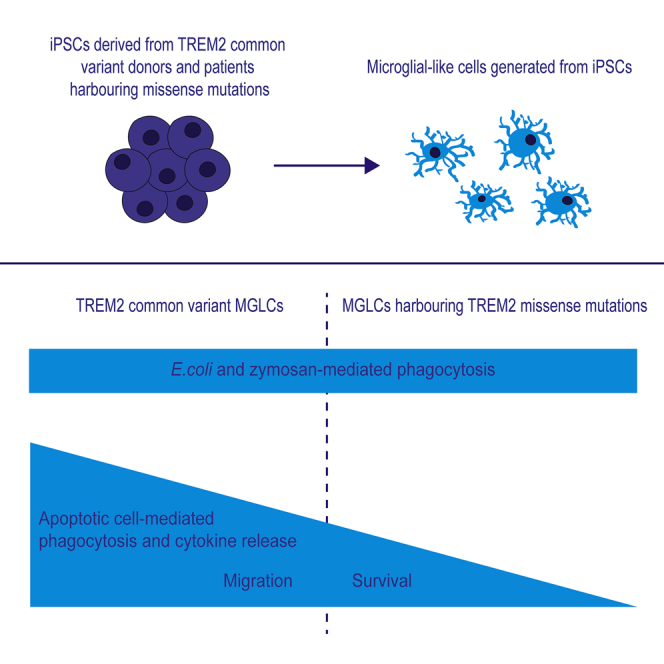

## Teaser

Garcia-Reitboeck et al. describe the generation of human induced pluripotent stem cell-derived microglia-like cells from patients with early-onset dementia caused by variants in the immune receptor gene *TREM2.* They observed functional deficits in TREM2 variant cells, including reduced soluble TREM2 secretion, selectively reduced phagocytosis of apoptotic neuronal cells, and a deficit in migratory capacity.

## Introduction

Microglia, the resident macrophages of the brain, fulfill a variety of functions in the development and maintenance of the CNS, including synaptic pruning, surveillance, response to noxious and infectious agents, and tissue repair (Wolf et al., 2017). A number of microglial genes have been identified as genetic risk factors for Alzheimer disease (AD) (Villegas-Llerena et al., 2016). The immune receptor triggering receptor expressed on myeloid cells-2 (TREM2) was recently identified as a genetic risk factor for AD (Guerreiro et al., 2013a, Jonsson et al., 2013). In addition, gene network analyses of human AD brains and mouse models of AD have highlighted a central role for microglia in AD and, in particular, TREM2 and its binding partner TYRO protein tyrosine kinase-binding protein (TYROBP), also known as DNAX-activation protein 12 (DAP12) DAP12/TYROBP (Matarin et al., 2015, Zhang et al., 2013). Whereas heterozygous variants in *TREM2* are associated with AD (Guerreiro et al., 2013a, Jonsson et al., 2013), homozygous variants in *TREM2* or its binding partner *DAP12/TYROBP* cause polycystic lipomembranous osteodysplasia with sclerosing leukoencephalopathy (PLOSL), also known as Nasu-Hakola disease (NHD). NHD is a rare autosomal-recessive early-onset dementia characterized by behavioral changes and cognitive decline, with or without pathological bone fractures (Guerreiro et al., 2013b, Paloneva et al., 2002). How TREM2 contributes to neurodegeneration remains poorly understood. Furthermore, studies investigating the impact of TREM2 signaling on the inflammatory response have produced conflicting results, demonstrating either an anti-inflammatory or a pro-inflammatory role for TREM2 (Hamerman et al., 2006, Jay et al., 2015, Jay et al., 2017, Sieber et al., 2013, Turnbull et al., 2006). Recent studies have identified a role for TREM2 in microglial survival (Wang et al., 2015), as well in controlling energy metabolism (Ulland et al., 2017). Several studies have identified a role for TREM2 in phagocytosis (Hsieh et al., 2009, Kawabori et al., 2015, Kleinberger et al., 2014, Takahashi et al., 2005, Xiang et al., 2016), although others have observed no effect (e.g., Wang et al., 2015). One possible explanation for some of these discrepancies may be species differences between rodent and human immune cells (Smith and Dragunow, 2014) or differences in phagocytic materials. To investigate the effects of dementia-causing *TREM2* missense mutations on human macrophage function, we took advantage of a recently developed protocol to derive macrophages from human induced pluripotent stem cells (iPSCs) (van Wilgenburg et al., 2013). These iPSC-macrophages were shown to arise through a transcription factor MYB-independent developmental pathway, similar to yolk sac-derived tissue-resident macrophages such as brain-resident microglia (Buchrieser et al., 2017). We confirmed that the iPSC-macrophages we isolated are in fact very similar to microglia by demonstrating the expression of microglial genes, and we therefore refer to them as iPSC-microglial-like cells (iPSC-MGLCs). We tested whether iPSC-MGLCs could be used to study the role of TREM2 in neurodegeneration by generating iPSC-MGLCs from two patients with NHD caused by homozygous T66M and W50C TREM2 variants, as well as two unaffected relatives harboring one T66M variant allele and four controls expressing common variant TREM2. We confirmed that iPSC-MGLCs express and shed soluble TREM2 (sTREM2) protein and provide the first report to assess the functional consequences of the recently described W50C mutation in our iPSC-MGLC model. We identify deficits in the ability of cells harboring TREM2 missense mutations to survive a macrophage colony stimulating factor (MCSF) starvation regimen, and furthermore, to identify a specific deficit in phagocytosis. Taken together, these data provide insights into specific pathways known to be aberrant in chronic neurodegenerative pathologies and link these pathways to TREM2.

## Results

### Generation of Human iPSC-MGLCs

**Figure 1:**
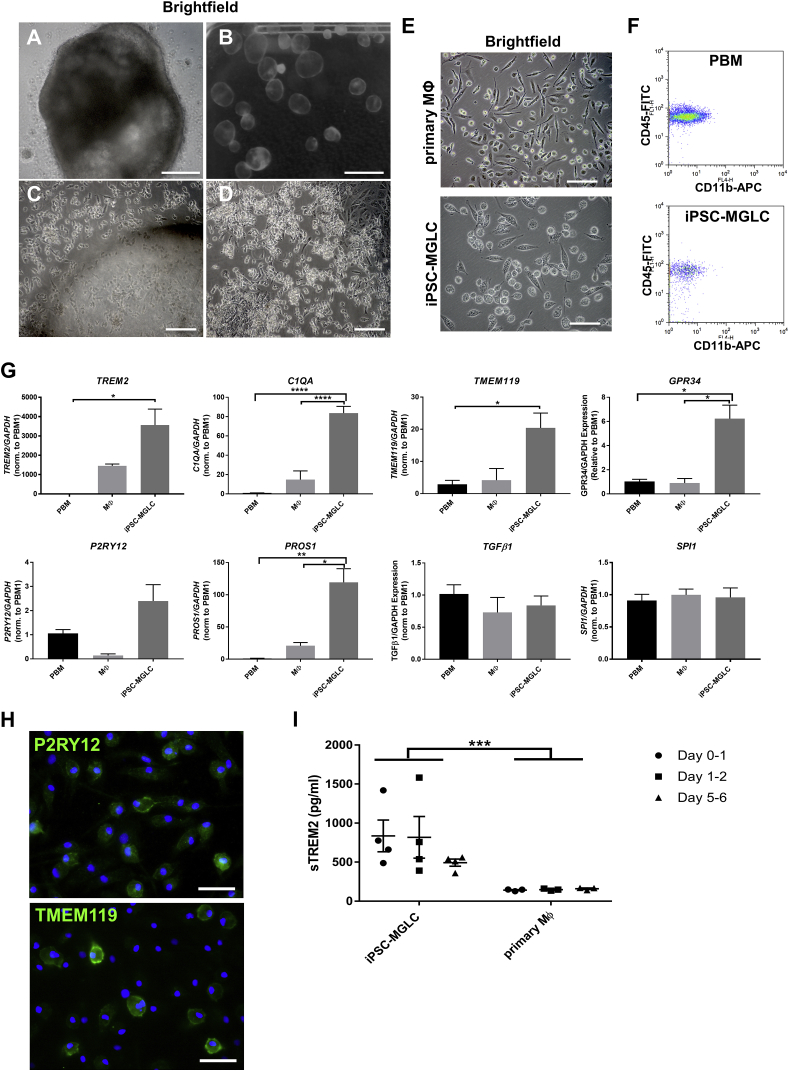
Generation and Characterization of iPSC-MGLCs (A and B) Brightfield microscopy of an embryoid body (EB) after generation in a 96 well low adherence plate (A) and free floating EBs forming large cystic structures during further culture in myeloid progenitor differentiation medium (B). (C and D) New iPSC-MGLCs are shed from either free-floating cystic EBs (C) or adherent cell layers (D). (E) Comparison of iPSC-MGLCs with primary Mϕ cultured in M-CSF by brightfield microscopy. (F) FACS analysis of the macrophage markers CD45 and CD11b in iPSC-MGLCs and primary blood-derived monocytes (PBMs), directly isolated from blood. (G) qPCR analysis of *SPI1*, *TGFβ1*, or *P2RY12* levels, as well as the microglial markers *TREM2*, *C1QA*, *GPR34*, and *TMEM119* in iPSC-MGLCs, freshly isolated PBMs, and primary Mϕ (^∗^p < 0.05, ^∗∗∗∗^p < 0.0001 using one-way ANOVA with Dunnett’s correction for multiple comparisons). (H) Immunocytochemistry with antibodies against the microglial proteins P2RY12 and TMEM119 in iPSC-MGLCs. (I) Secreted sTREM2 levels measured by ELISA from supernatant collected at days 1, 2, and 6 in iPSC-MGLCs and primary Mϕ. ^∗∗∗^p < 0.001 for each cell type using two-way ANOVA. Scale bars: 100 μm in (C)–(E), 250 μm in (A), 5 mm in (B), and 50 μm in (H). Data are represented as means ± SEMs; n = 3; ^∗∗^p < 0.01. See also Figures S2, S3, and S4.

We generated iPSC-MGLCs using recently developed macrophage differentiation protocols (van Wilgenburg et al., 2013), with minor modifications as detailed in the Supplemental Experimental Procedures. By generating embryoid bodies (EBs) in ultralow adherence 96-well plates (Figure 1A), we could reliably generate several million iPSC-MGLCs per week. Most EBs floated and generated large cystic structures (Figures 1B and 1C) or sometimes adhered to the bottom of the flasks (Figure 1D). Like other investigators (Hale et al., 2015, van Wilgenburg et al., 2013), we noticed the appearance of smaller-diameter cells 10–14 days after seeding EBs in myeloid progenitor medium containing MCSF and interleukin-3 (IL-3) that did not attach to tissue culture plates (not shown). Three to 4 weeks after seeding the EBs, the free-floating small cells were replaced by cells of a larger diameter, with fine processes that subsequently adhered to tissue culture plates and differed in morphology from primary macrophages (Mϕ) (Figure 1E), and they expressed similar levels of the myeloid markers CD45 and CD11b when compared to primary blood-derived monocytes (PBMs; Figure 1F). These cells could be harvested on a weekly basis, with several million iPSC-MGLCs being harvested from one 175-cm^2^ flask containing approximately 150 EBs.

### iPSC-MGLCs Express and Shed TREM2 and Have a Microglial Gene Signature

A recent study found that macrophages derived from iPSCs using this protocol are ontogenetically similar to tissue resident macrophages such as microglia (Buchrieser et al., 2017). We therefore investigated whether the iPSC-MGLCs generated here expressed the tissue-resident macrophage marker *TREM2* and other microglial genes. Expression of *TREM2*, as well as complement factor *C1QA*, *TMEM119*, *GPR34, PROS1*, genes that are preferentially expressed in microglia as opposed to monocytes (Butovsky et al., 2014), were higher in iPSC-macrophages than in PBM and/or primary Mϕ (Figure 1G: ^∗^p < 0.05, ^∗∗^p < 0.01, ^∗∗∗∗^p < 0.0001). The levels of *SPI1* (encoding for the myeloid transcription factor PU1) and another microglial gene, *TGFb1*, were comparable between groups, whereas we observed a trend toward higher mRNA levels of P2RY12, another marker for microglia, in iPSC-macrophages compared with PBM and primary Mϕ (Figure 1G). Immunocytochemistry, with antibodies against the microglial genes P2RY12 and TMEM119, confirmed expression at the protein level of these microglial markers (Figure 1H). We also investigated whether iPSC-MGLCs shed sTREM2 into the extracellular space, as has been reported for cell lines and murine microglia (Kleinberger et al., 2014, Wunderlich et al., 2013). Levels of sTREM2 were measured in supernatants of iPSC-MGLCs and primary Mϕ at day 1 (D1), D2, and D6 in culture, and it was found that sTREM2 levels from iPSC-MGLCs were consistently higher than primary Mϕ in a period of 6 days (Figure 1I; iPSC-MGLC: 835 ± 203 pg/mL versus PBM 141 ± 6 pg/mL on D1, iPSC-MGLC 817 ± 266 pg/mL versus PBM 147 ± 7 pg/mL on D2, and iPSC-MGLC 492 ± 45 pg/mL versus PBM 159 ± 9.5 pg/mL on D6 of culture; ^∗∗∗^p < 0.001).

In conclusion, our results demonstrate that iPSC-MGLCs differ substantially from PBMs and primary Mϕ through expression of a microglial gene signature, protein expression, and the functional shedding of TREM2. Taken together, these data allow us to refer to the cells as iPSC-MGLCs.

### Generation of iPSC-MGLCs Harboring TREM2 Variants

**Figure 2:**
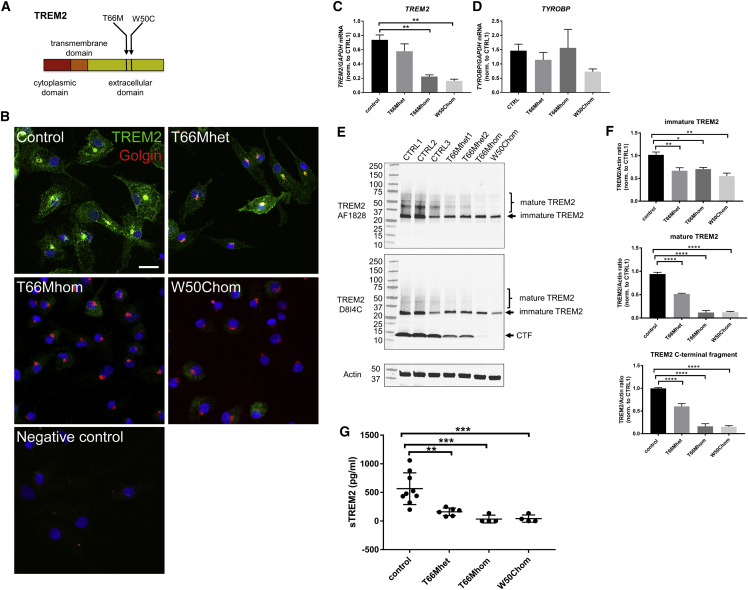
Characterization of TREM2 Expression and Secretion from Controls and TREM2 Variant iPSC-MGLCs (A) Diagram of TREM2, indicating the T66M and W50C variants in the extracellular domain of TREM2. (B) Immunocytochemistry and confocal imaging for TREM2 in control and TREM2 T66Mhet iPSC-MGLC and in TREM2 T66Mhom and W50Chom iPSC-MGLC. Scale bar: 20 μm. (C and D) qPCR of *TREM2* (C) and *TYROBP/DAP12* (D) mRNA expression in control, T66Mhet, T66Mhom, and W50Chom iPSC-MGLCs (^∗∗^p < 0.01). (E) Western blot analysis of immature (arrows) and mature, glycosylated TREM2 protein (brackets) in *TREM2* T66Mhet, T66Mhom, and W50Chom compared with controls. D8I4C antibody detects a C-terminal fragment (CTF) of TREM2 (arrow labeled CTF). (F) Quantification of TREM2 WB bands with optical densitometry; immature TREM2 levels reduced 34%, 30%, and 38%; mature TREM2 levels reduced 45%, 87%, and 86%; and TREM2 CTF levels reduced 39%, 83%, and 84% in T66Mhet, T66Mhom, and W50Chom iPSC-MGLCs compared with controls (n = 3, one clone per iPSC line, ^∗^p < 0.01, ^∗∗^p < 0.001, ^∗∗∗∗^p < 0.0001). (G) Quantification of sTREM2 secretion measured by ELISA after 24 hr in culture in T66Mhet, T66Mhom, and W50Chom iPSC-MGLCs compared with controls (control: 565 ± 92 pg/mL, T66Mhet: 160 ± 27 pg/mL, T66Mhom: 33 ± 33 pg/mL, W50Chom: 42 ± 31, ^∗∗^p < 0.01, ^∗∗∗^p < 0.001). Data are means ± SEMs. See also Figures S1, S2, S3, S4, and S5.

To investigate the effects of TREM2 variants on microglial function, iPSC-MGLCs were generated from a patient with NHD caused by a homozygous TREM2 T66M variant, two unaffected relatives carrying a heterozygous TREM2 T66M variant, and one NHD patient carrying a homozygous TREM2 W50C variant (Table 1; Dardiotis et al., 2017, Guerreiro et al., 2013b). Both mutations reside in the extracellular domain of *TREM2* (Figure 2A). TREM2 mutant iPSCs were characterized in regard to karyotype and copy-number variation (CNV; Figure S1) pluripotency and genotype (Brownjohn et al., 2018). Four iPSC control lines were used for comparison (Table 1). Sanger sequencing of *TREM2* exon 2 confirmed the homozygous and heterozygous T66M variants and the homozygous W50C variant in the TREM2 mutant lines (Figure S2A). Two clones per line were used in all of the experiments.

**Table 1:** Characteristics of TREM2 Variant and Control Common Variant iPSC Lines

iPSC Line	Age, y	Sex	Clinical Details	TREM2 Variant	ApoE Status
CTRL1	78	M	control	common variant	apoE3/E3
CTRL2	64	M	control	common variant	apoE2/E3
CTRL3	36	F	control	common variant	apoE2/E3
CTRL4	67	F	control	common variant	apoE3/E3
T66Mhet1	75	F	unaffected relative of T66Mhom	heterozygous T66M	apoE2/E3
T66Mhet2	47	M	unaffected relative of T66Mhom	heterozygous T66M	apoE3/E3
T66Mhom	51	M	NHD	homozygous T66M	apoE2/E3
W50Chom	36	F	NHD	homozygous W50C	apoE3/E3

NHD, Nasu-Hakola disease.

The morphology of controls and TREM2 variant iPSC-MGLCs was similar (Figure S2B), as was the expression of the myeloid markers CD45 and CD11b (Figure S2C). The weekly iPSC-MGLC yield did not differ between control and TREM2 variant iPSC (Figure S2D). Immunocytochemistry confirmed that control and TREM2 variant iPSC-MGLCs displayed similar staining patterns of the macrophage markers Iba1 and CD68 (Figure S3A) and CD45 and the myeloid transcription factor PU.1 (Figure S3B). Overall, no consistent difference in the expression of microglial genes or AD risk factor genes was observed between controls and TREM2 variant iPSC-MGLCs (Figure S4A). Furthermore, using fluorescence-activated cell sorting (FACS) analysis, we did not detect a difference in CD33 or CSF1R surface expression between TREM2 variant iPSC-MGLCs (Figure S4B).

### Reduced TREM2 Expression and Shedding in TREM2 Variant iPSC-MGLC

The subcellular localization of TREM2 in TREM2 variant iPSC-MGLCs was determined by confocal microscopy. In control iPSC-MGLCs, the staining pattern of TREM2 was diffuse in the cytosol, with additional prominent focal staining that colocalized with the Golgi complex (Figure 2B). TREM2 staining in T66Mhet iPSC-MGLC was similar to controls, with diffuse cytosolic staining as well as prominent perinuclear staining of TREM2 (Figure 2B). A significant reduction in TREM2 staining was observed in T66M homozygous (T66Mhomo) iPSC-MGLC and W50C iPSC-MGLCs, with an absence of focal staining (Figure 2B). *TREM2* mRNA levels did not differ between controls and T66Mhet iPSC-MGLCs, but they were markedly reduced in T66Mhom and W50Chom iPSC-MGLC (Figure 2C: ^∗∗^p < 0.01). mRNA levels of the TREM2-binding protein *TYROBP/DAP12* did not differ between control and TREM2 variant iPSC-MGLCs (Figure 2D). Western blot analysis further demonstrated a reduction in TREM2 protein levels in both T66Mhet iPSC-MGLCs and T66Mhom/W50Chom iPSC-MGLCs (Figures 2E and S5A). The levels of immature TREM2 (Figures 2E and S5A, arrows) and mature, glycosylated TREM2 (Figures 2E and S5A, brackets) were reduced in TREM2 variant iPSC-MGLCs. Using antibody D8I4C, which also recognizes the C terminus of TREM2, we observed the presence of the C-terminal fragment (CTF) of TREM2 (resulting from protease-mediated cleavage of the protein at the cell membrane) (Wunderlich et al., 2013) and identified a significant reduction in T66Mhet, T66Mhom, and W50C iPSC-MGLCs compared with control iPSC-MGLCs, which is suggestive of altered processing of mutant TREM2 protein (Figures 2E and 2F). Quantification of TREM2 western blot (WB) bands with optical densitometry confirmed a significant reduction of immature, mature, and CTF TREM2 levels (Figure 2F: ^∗^p < 0.01, ^∗∗^p < 0.001, ^∗∗∗∗^p < 0.0001).

Because of the reduced maturation of TREM2 observed here, we investigated whether TREM2 variants influenced the secretion of sTREM2 in our model. Cerebrospinal fluid sTREM2 levels were found to be significantly reduced in patients with dementia harboring NHD-associated TREM2 variants (Piccio et al., 2016), and reduced shedding of sTREM2 was observed in human kidney cell lines overexpressing NHD-associated mutant forms of TREM2 (Kleinberger et al., 2016). However, data on the secretion of sTREM2 in human iPSC-MGLCs expressing endogenous levels of mutant TREM2 protein are lacking. Using an in-house ELISA system, we measured sTREM2 levels in supernatants of control and TREM2 variant iPSC-MGLCs. sTREM2 levels were markedly reduced in T66Mhet iPSC-MGLC lines and were virtually absent in T66Mhom and W50Chom iPSC-MGLCs compared with controls (Figures 2G and S5B). Together, these results demonstrate that *TREM2* mRNA and protein expression, maturation, and shedding of sTREM2 are markedly affected by *TREM2* missense mutations in iPSC-MGLCs.

### Growth Factor Withdrawal Triggers Cell Death in TREM2 Homozygous Missense iPSC-MGLCs

**Figure 3:**
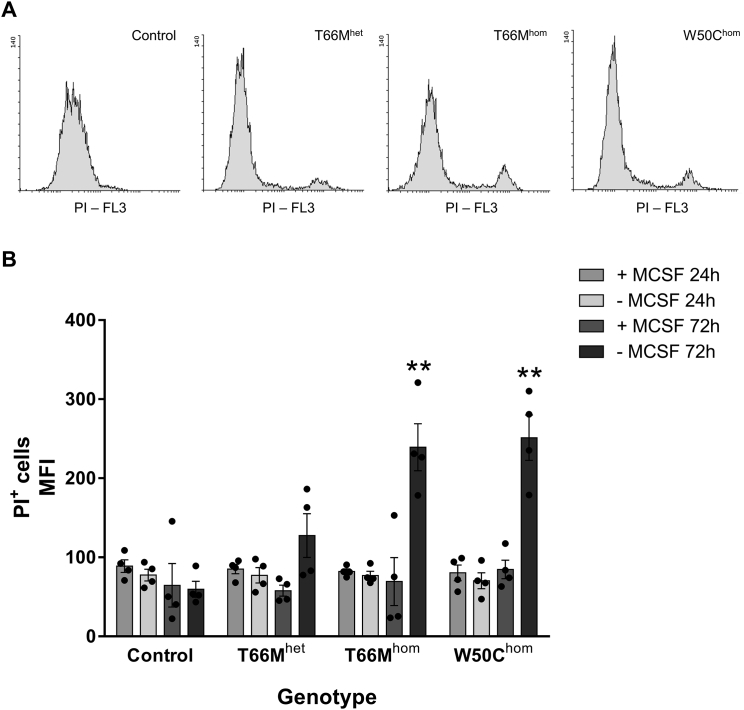
Survival in Control and TREM2 Variant iPSC-MGLCs (A) FACS analysis of propidium iodide (PI) staining of iPSC-MGLCs following 24–72 hr incubation in DMEM ± 100 ng/mL M-CSF. Representative histograms from 72 hr DMEM minus M-CSF group (n = 4). (B) Two to four control iPSC lines and 1–2 iPSC clones per TREM2 variant iPSC lines were analyzed, and mean fluorescence intensity was plotted. Two-way ANOVA with Dunnett’s multiple comparison was used for analysis. Data are presented as means ± SEMs; ^∗∗^p < 0.01.

Previous studies have demonstrated an increased sensitivity of murine TREM2 knockout microglia to the withdrawal of M-CSF, leading to cell death (Wang et al., 2015). iPSC-MGLCs were cultured for 24 and 72 hr in the presence or absence of 100 ng/mL M-CSF. Staining with the cell dye propidium iodide (PI), a marker of the loss of cell membrane integrity, was measured with FACS. No difference in PI uptake was observed either under basal conditions or following withdrawal of M-CSF for 24 hr between control and TREM2 variant iPSC-MGLCs (Figure 3A). However, after 72 hr withdrawal of M-CSF, T66Mhom cells showed a significant increase in PI uptake compared with controls, indicating an increased sensitivity to growth factor withdrawal, leading to cell death (Figure 3B).

### Cytokine Secretion in Response to LPS in TREM2 Variant iPSC-MGLCs Does Not Differ from Controls

**Figure 4:**
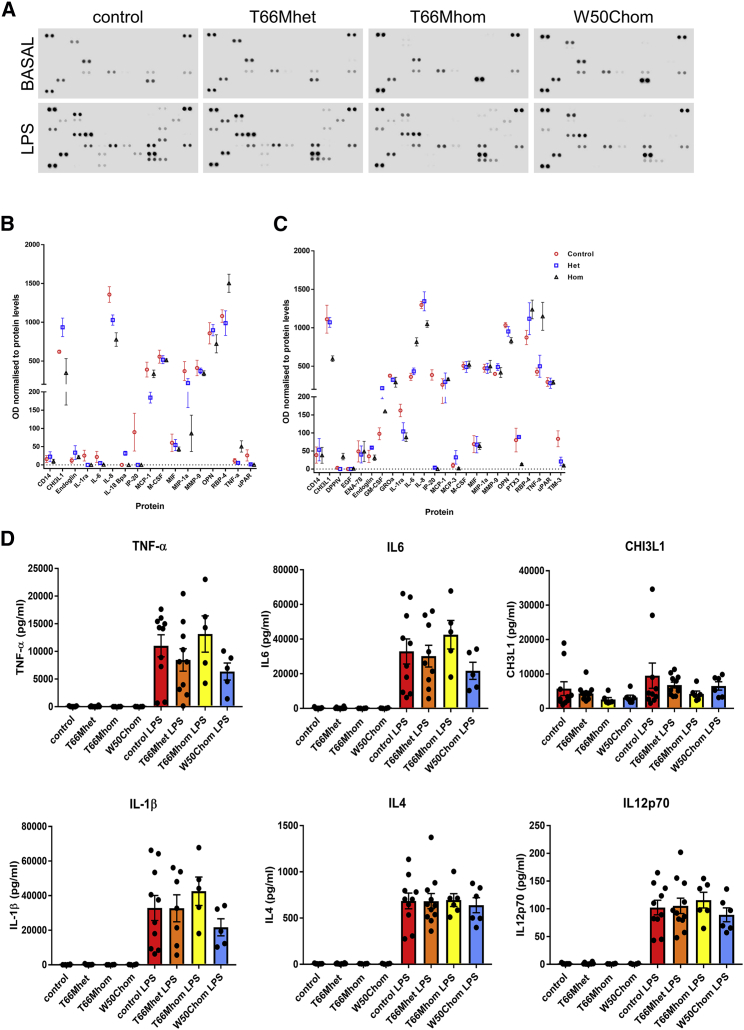
Analysis of Secreted Factors from Controls and TREM2 Variant iPSC-MGLCs after LPS Exposure (A) Proteome profiles of secreted factors from control, T66Mhet, T66Mhom, and W50Chom iPSC-MGLCs analyzed using a Human XL Cytokine Array Kit (R&D Systems). Representative dot blots from iPSC-MGLC culture supernatants from one control line, one T66Mhet line, one T66Mhom, and W50Chom iPSC-MGLCs are shown under basal (untreated) conditions and LPS-treated conditions; 100 ng/mL after 18 hr. (B and C) Analysis of dot blot intensity from supernatants of basal (B) and LPS-treated (C) cultures. Mixed supernatants from independent experiments were used; n = 3 for all genotypes. (D) Levels of 10 cytokines, including TNF-α, IL-6, CHI3L1, IL-1β, IL-4, and IL-12p70 were measured simultaneously on the Meso Scale Diagnostics (MSD) platform using a V-PLEX Pro-inflammatory Panel 1 (n = 2 for untreated, n = 3 for LPS-treated samples in four control iPSC lines, and two clones per TREM2 variant iPSC line were analyzed; data are presented as mean ±SEM). See also Figure S6.

The effect of TREM2 variants on the secretion of inflammatory cytokines in response to pro-inflammatory stimuli was determined, as previous studies report opposing effects of TREM2 on inflammation (Sieber et al., 2013, Turnbull et al., 2006). Using lipopolysaccharide (LPS), a well-characterized activator of Toll-like receptor-4 (TLR-4) and a binding partner of TREM2 (Daws et al., 2003), as a stimulant to activate iPSC-MGLCs, we undertook proteome analysis of 105 cytokines in two controls, 2 × T66Mhet lines, 1 × T66Mhom, and 1 × W50C iPSC clones (Figures 4A–4C, S6A, and S6B). An increase in the secretion of a number of cytokines, including tumor necrosis factor-α (TNF-α), IL-6, IL-8, and chitinase-3-like protein 1 (CHI3L1, also known as YKL-40), was observed following LPS stimulation, with small differences in release observed in T66Mhet and T66Mhom variant lines (Figures 4A–4C, S6A, and S6B). To investigate these differences more quantitatively, we used a V-PLEX Proinflammatory Panel 1 Human Kit (Meso Scale Diagnostics; Figures 4D and S6C). In this more sensitive assay, we found no significant differences in the levels of these cytokines between controls, T66Mhet, T66Mhom, and W50Chom iPSC-MGLCs, both under basal conditions and following LPS secretion (Figures 4D and S6C). Thus, *TREM2* missense mutations do not affect cytokine secretion under basal conditions or in the context of TLR-4 stimulation in human iPSC-MGLCs.

### iPSC-MGLCs Harboring Missense *TREM2* Mutations Show Deficits in Phagocytosis

**Figure 5:**
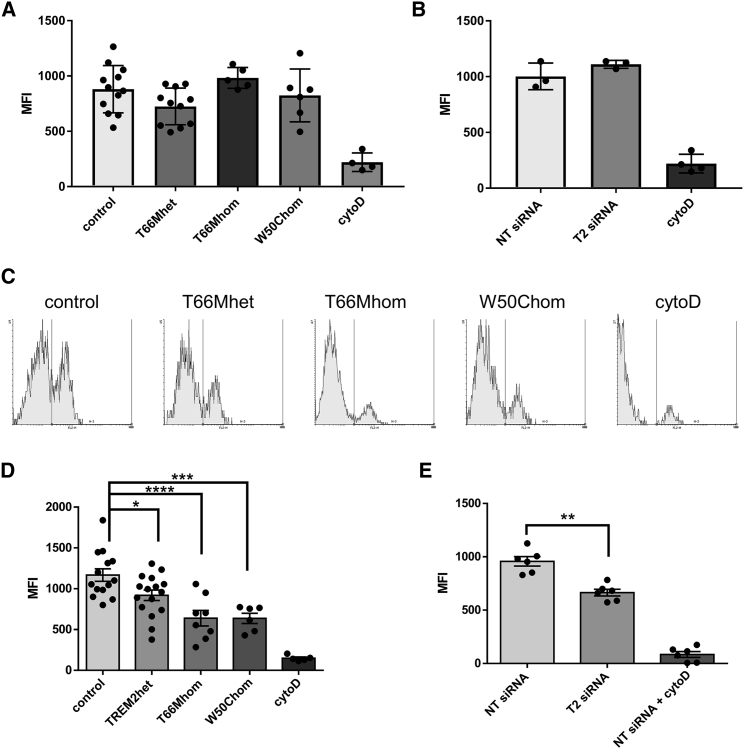
Analysis of Pathogen- and Apoptotic Cell-Associated Phagocytosis (A) FACS analysis to measure uptake of pHrodo *E. coli* particles in variant TREM2 iPSC-MGLCs compared with controls. n = 3, four control iPSC lines and two iPSC clones per TREM2 variant iPSC line were analyzed. (B) FACS analysis to measure uptake of pHrodo *E. coli* particles following siRNA knockdown of TREM2 in iPSC-MGLCs (T2 siRNA) or non-targeting siRNA (non-targeting [NT] siRNA) (n = 3). (C) FACS analysis of phagocytosis of fluorescent dye-labeled apoptotic SH-SY5Y cells by control and TREM2 variant iPSC-MGLCs. Quantification of phagocytosis shown. (D) 21%, 45%, and 45% reduction in T66Mhet, T66Mhom, W50Chom, respectively, compared with controls (n = 4, ^∗^p < 0.5, ^∗∗∗^p < 0.001, ^∗∗∗∗^p < 0.0001; four control iPSC lines and two iPSC clones per TREM2 variant iPSC lines were analyzed). (E) FACS analysis of phagocytosis of fluorescent dye-labeled apoptotic SH-SY5Y cells in iPSC-MGLC treated with TREM2 siRNA (T2 siRNA) or non-targeting siRNA (NT siRNA). Presented siRNA data are from experiments using one control iPSC line for pHrodo *E. coli* or pooled normalized data from two control iPSC lines (for phagocytosis of apoptotic neuronal cells). Student’s t test was used for the analysis of siRNA experiments and one-way ANOVA with Dunnett’s correction for comparison between control and TREM2 variant iPSC-MGLC; data are presented as mean ± SEM. See also Figure S7.

Because recent studies using TREM knockout (KO) mice and genetically manipulated cell lines have reported conflicting findings regarding the effect of TREM2 on phagocytosis (Kleinberger et al., 2014, Wang et al., 2015, Xiang et al., 2016), we investigated whether phagocytosis was impaired in human TREM2 variant iPSC-MGLCs. Non-evoked phagocytic activity was measured using fluorescent pH-sensitive rhodamine *Escherichia coli* (pHrodo *E. coli*), using FACS analysis. Cytochalasin D, a widely used inhibitor of phagocytosis, abolished the uptake of *E. coli* particles (Figures 5A and 5B). No significant differences were observed between T66Mhet, T66Mhom, and W50Chom iPSC-MGLCs when compared with control iPSC-MGLCs (Figure 5A). Furthermore, phagocytosis of pHrodo *E. coli* particles in iPSC-MGLCs (control cells) that had been treated with TREM2 or non-targeting small interfering (siRNA) showed no significant differences (Figure 5B), with the TREM2 knockdown being confirmed with qPCR, WB, and sTREM2 ELISA (Figures S7A–S7C). We also observed no difference in the phagocytosis of pHrodo zymosan, a putative ligand of the TLR-2 and TLR-6 receptors, when comparing genotypes (Underhill, 2003).

Because TREM2 binds phospholipids, including phosphatidylserine (Wang et al., 2015), which becomes exposed on the surface of apoptotic cells, we hypothesized that TREM2 may affect microglial phagocytosis in a substrate-specific way. We therefore measured the phagocytosis of apoptotic Vybrant CM-Dil dye-labeled human neuronal SH-SY5Y cells by FACS. Apoptosis was induced with UV radiation, and the presence of phosphatidylserine on the surface of apoptotic cells was confirmed with fluorescently labeled annexin V (Figure S7E). Cytochalasin D significantly inhibited the uptake of apoptotic SH-SY5Y cells (Figures 5C–5E). Comparing with controls, we found a marked reduction in the phagocytosis of apoptotic neuronal cells in T66Mhet, T66Mhom, and W50Chom (Figures 5C and 5D: ^∗^p < 0.5, ^∗∗∗^p < 0.001, ^∗∗∗∗^p < 0.0001). We also found a significant reduction in apoptotic cell uptake in TREM2 siRNA versus non-targeting siRNA-treated iPSC-MGLCs, confirming a TREM2-specific effect (Figure 5E: ^∗∗^p < 0.01). These data, taken together, suggest a substrate-specific deficit in phagocytic ability that is dependent on the TREM2 genotype or, more specifically, the availability of TREM2 protein for signaling.

### iPSC-MGLCs Harboring Missense *TREM2* Mutations Show Deficits in Chemokine Release

**Figure 6:**
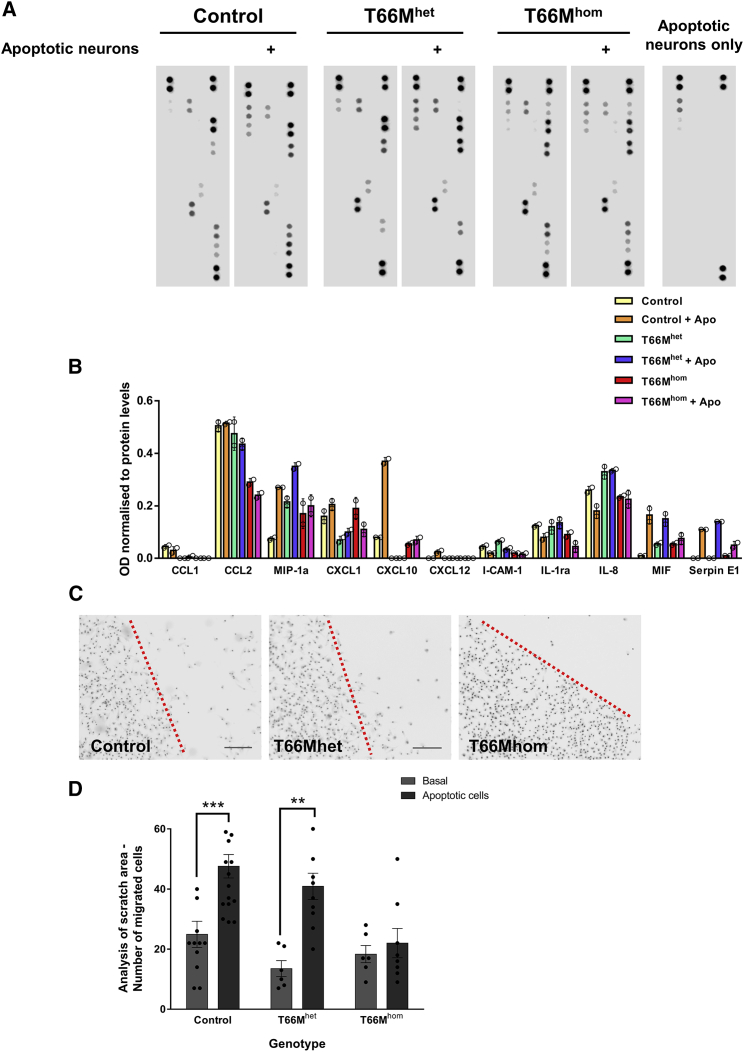
Apoptotic Cell-Mediated Cytokine Responses Are Dependent on Genotype (A) Proteome profiles of secreted factors from control, T66Mhet, and T66Mhom iPSC-MGLCs were analyzed using a human cytokine array. Representative dot blots from pooled cell culture supernatants from two control, T66Mhet, and T66Mhom lines, and apoptotic cells only are shown under basal (untreated) conditions and following exposure to apoptotic SH-SY5Y cells for 48 hr. (B) Analysis of dot blot intensity from supernatants of basal cell- and apoptotic cell-exposed cultures revealed modulation of secreted factors associated with chemotaxis and chemoattraction with a dependence on genotype (C and D) Mixed supernatants from independent experiments were used; n = 3 for all genotypes. Representative images from cell culture scratch assays. Scale bar: 200 μm (C). Cultures were imaged live after 24h exposure to trans-wells containing apoptotic SH-SY5Y and subsequent Hoechst staining (D). Analysis was performed using ImageJ thresholding and particle quantification of the scratch area in (D). Two-way ANOVA with Bonferroni multiple comparison was used for analysis. Data are presented as means ± SEMs from two control lines, and one T66Mhet, and one T66Mhom line; n = 3 (^∗∗^p < 0.01, ^∗∗∗^p < 0.001). See also Figure S7.

Deficits in the ability of cells harboring TREM2 missense mutations to phagocytose apoptotic cells suggest either aberrant ligand recognition, defects in the cells’ ability to efficiently migrate to areas of damage, or both. Using a proteome profiler array, we identified clear deficits in the release of particular cytokines related to chemotaxis and chemoattraction, namely MIP1α and CXCL10, from cells harboring the T66Mhom mutation when exposed to apoptotic cells (Figures 6A, 6B, S7F, and S7G). Furthermore, scratch assays to determine the ability of cells to migrate toward an area of damage, in this case a concentration of apoptotic cells, identified a significant deficit in iPSC-MGLCs from T66Mhom patient lines (Figures 6C and 6D). Taken together, these data strongly suggest a global deficit in the ability of cells harboring TREM2 missense mutations to efficiently respond to signaling associated with neurodegenerative pathologies.

## Discussion

Transcriptome analysis indicated that the macrophages or microglial-like cells derived from iPSCs have a high expression of genes associated with tissue-resident macrophages (Alasoo et al., 2015). A recent study using clustered regularly interspaced short palindromic repeats (CRISPR)/CRISPR-associated protein-9 nuclease (Cas9) demonstrated that iPSC-macrophages develop in an MYB-independent manner that is characteristic of tissue-resident macrophages, including microglia (Buchrieser et al., 2017). Furthermore, another recent study demonstrated that iPSC-microglia generated with the same protocol used in the present study have a gene transcription profile that is very similar to primary human microglia, which can be brought even closer by co-culturing with neurons (Haenseler et al., 2017). In addition to the tissue-resident macrophage marker *TREM2*, we found that our iPSC-MGLCs expressed *C1QA*, *TMEM119*, *GPR34*, and *PROS1*, which were previously identified as microglial markers (Butovsky et al., 2014). Whereas the mRNA levels of the microglial gene *P2RY12* were not markedly different from blood-derived monocytes, we found that iPSC-MGLCs nevertheless showed positive staining for P2RY12 by immunocytochemistry. Differences in the transcriptome between microglia, the resident macrophages of the brain, and macrophages were recently reported (Butovsky et al., 2014, Hickman et al., 2013); it is worth noting, however, that microglia and other macrophages share many similarities, including the ability to phagocytose and secrete pro-inflammatory cytokines. Recently, other protocols to generate human iPSC-MGLCs have been described (Muffat et al., 2016, Pandya et al., 2017). We used a protocol that allows the robust generation of large numbers of iPSC-MGLCs from several iPSC lines. We have shown that these iPSC-MGLCs serve as a strong model to study the impact of *TREM2* variants on phagocyte function and have demonstrated a strong deficit in the ability of iPSC-MGLCs harboring TREM2 missense mutations to phagocytose endogenously associated debris, with deficits observed in the ability to efficiently migrate to areas mimicking pathology.

Initial characterization identified mRNA, and protein expression, maturation, and shedding of sTREM2 into the extracellular space are markedly reduced in T66Mhet, T66Mhom, and W50Chom, with virtually undetectable sTREM2 in TREM2 homozygous iPSC-MGLCs. Given the recently developed role of sTREM2 in promoting macrophage survival and inflammatory responses (Wu et al., 2015, Zhong et al., 2017), the reduction or absence of sTREM2 is likely to contribute to macrophage dysfunction in NHD. Reduced cleavage of TREM2 from the cell surface has also been shown to affect TREM2 intracellular signaling (Glebov et al., 2016, Wunderlich et al., 2013). Further studies are needed to determine whether sTREM2 influences other aspects of microglial function and interactions of microglia with other brain cells. *TREM2* mRNA levels were previously found to be reduced in patients with NHD caused by TREM2 variants (Chouery et al., 2008, Sasaki et al., 2015), and we found this to be the case for T66Mhom and W50Chom but not for T66Mhet iPSC-MGLCs. We found no difference in the mRNA and protein surface expression level of the dementia-causing or associated genes *CSF1R* and *CD33*. It was recently reported that *CD33* modulates TREM2 surface expression in human peripheral blood mononuclear cells (Chan et al., 2015) and that TREM2 KO macrophages have increased apoptosis in response to the withdrawal of CSF-1 (Wu et al., 2015). At least under basal conditions, we found no impact of NHD-causing TREM2 variants on these 2 AD-associated macrophage receptors in iPSC-MGLCs. However, after analyzing the sensitivity of TREM2 variant iPSC-MGLCs to growth factor withdrawal, we observed an increase in cell death in TREM2 homozygous missense mutation-harboring iPSC-MGLCs, when starved for 72 hr (three times the length of time used for TREM2 KO mouse microglia; Wang et al., 2015).

No deficits were observed in non-endogenous phagocytic ligands of *E. coli* or zymozan-mediated cytokine release, suggesting that TREM2 missense mutations alone are not sufficient to alter these phagocytic mechanisms. This was further supported by siRNA studies that showed no difference in *E. coli* phagocytosis. However, phagocytosis of apoptotic neuronal cells was markedly reduced in both *TREM2* heterozygous and homozygous iPSC-MGLCs in a gene dosage-dependent manner and confirmed with siRNA knockdown studies. The fact that TREM2 heterozygous variants in particular impair the phagocytosis of apoptotic cells is of relevance to AD, in which heterozygous TREM2 variants (mainly the R47H variant) were found to be associated with AD (Guerreiro et al., 2013a, Jonsson et al., 2013), although further studies with cells from patients harboring this specific mutation are required for confirmation. A possible explanation for the observed substrate-dependent impairment in phagocytosis may be that *E. coli* or zymosan particles, known ligands of TLR4 and TLR2, respectively, do not engage the TREM2 receptor, whereas apoptotic cells (possibly through phosphatidylserine and other phospholipid molecules on their surface) do, and could therefore induce TREM2 signaling, which may boost phagocytic capability. Our results using human iPSC-MGLCs extend previous studies looking at the effects of TREM2 on phagocytosis, including those in TREM2-overexpressing cell lines, isolated microglia from TREM2 KO mice, and CRISPR-Cas9-manipulated microglial cell lines (Atagi et al., 2015, Kleinberger et al., 2014, Takahashi et al., 2005, Xiang et al., 2016). We saw a reduction in the phagocytosis of apoptotic cells in all TREM2 disease variants (including two clones per TREM2 variant iPSC line), with a gene dose response in TREM2 variants, strongly suggesting that these data did not result from other human genetic variability.

The strength of our approach lies in the fact that we have studied the biology of TREM2 in patient-derived iPSC-MGLCs that harbor NHD-causing TREM2 variants and express TREM2 at endogenous levels. We conclude that these iPSC-MGLCs serve as a strong model to study TREM2 and other myeloid genes associated with neurodegenerative disease, including NHD and AD. Both TREM2 heterozygous and homozygous variants affected the phagocytosis of apoptotic cells, with observations from cytokine array profiles suggesting deficits in chemotaxis and chemoattractant pathways when harboring a TREM2 homozygous mutation, which were supported by deficits in functional migration studies and thus supported previously published data (Mazaheri et al., 2017). Further work investigating the mechanisms of how TREM2 influences the reaction of microglia to neuronal injury may shed light on the microglial dysfunction in neurodegenerative diseases and identify suitable drug targets for future treatments.

## Experimental Procedures

### Generation of Human iPSC-Derived Microglia-Like Cells

TREM2 variant primary fibroblast lines were generated from 4 mm skin punch biopsies, obtained under informed consent. Ethical permission for this study was obtained from the National Hospital for Neurology and Neurosurgery and the Institute of Neurology joint research ethics committee (study reference 09/H0716/64) or approved by the ethics committee of the Istanbul Faculty of Medicine, Istanbul University (for collection of T66M mutant fibroblasts to Dr. Ebba Lohmann). iPSCs were generated from fibroblast cultures using 4-factor Sendai virus reprogramming at The Gurdon Institute and the Department of Biochemistry, University of Cambridge (Cambridge, UK). TREM2 mutations were confirmed with Sanger sequencing (Figure 3A), and pluripotency was confirmed (Brownjohn et al., 2018). Human iPSC-derived MGLCs were generated using recently published protocols (van Wilgenburg et al., 2013), with minor modifications (see Supplemental Experimental Procedures).

### Isolation of Human Blood-Derived Monocytes

Monocytes were obtained from blood through centrifugation with Histopaque (Sigma, USA) to isolate peripheral blood mononuclear cells followed by separation with CD14-labeled magnetic beads (Miltenyi, USA). Primary blood-derived monocytes (PBMs) were either analyzed immediately or matured into primary macrophages (Mϕ) by incubation with macrophage end-differentiation medium: X-Vivo 15 medium (Lonza, Switzerland) with 1% GlutaMAX (Life Technologies, USA), 100 U penicillin/streptomycin (Life Technologies), and 100 ng/mL M-CSF (PeproTech, USA).

### sTREM2 ELISA

iPSC-MGLCs were seeded at 4 × 10^4^ cells/well in 96-well plates (Corning, USA) in macrophage end-differentiation medium. The following day, the medium was changed to fresh medium, and supernatants were collected after 24 hr. Quantification of sTREM2 from cell culture supernatants was performed using an in-house-generated ELISA using MaxiSORP 96-well plates (Nunc, Thermo Fisher Scientific, USA) coated with 1 μg/mL of a rat anti-mouse/human TREM2 monoclonal antibody (clone 237920, R&D Systems, USA) overnight at 4°C. After a blocking step, cell culture supernatant samples and standards (recombinant human TREM2-His; Life Technologies) were incubated for 2 hr at room temperature (RT) with biotinylated polyclonal goat anti-human TREM2 capture antibody (0.1 μg/mL; AF1828, R&D Systems). After incubation with streptavidin-horseradish peroxidase (HRP) (0.1 μg/mL; Invitrogen), followed by the addition of a chromogenic substrate solution (TMB, Life Technologies), the reaction was terminated with the addition of stop solution (0.16 M H_2_SO_4_) and absorbance was read at 450 nm (GENios, Tecan, USA). In addition, a second method using a mesoscale assay validated the results (see Supplemental Experimental Procedures).

### FACS

At least 1 × 10^5^–1 × 10^6^ cells per iPSC line were used for FACS analysis. Cells were harvested into PBS without Ca^2+^/Mg^2+^. Cells were washed with FACS buffer (PBS + 0.5% BSA (Sigma) + 2 mM EDTA), incubated with Fc block (1:20, Miltenyi) and primary antibodies (1:10) or isotype control antibodies (1:10) for 1 hr at 4°C, washed three times with FACS buffer, and analyzed using a Becton Dickinson FACSCalibur analyzer. The results were analyzed using FlowJo software (version 8.8.7; Tree Star, USA).

### PI Cell Death Assay

iPSC-MGLCs were seeded 3 days before the assay in 6-well plates (Corning) at 5 × 10^5^ cells/well in macrophage end-differentiation medium. The day before the assay, the medium was changed to DMEM (Life Technologies), with or without 100 ng/mL M-CSF. After 24–72 hr, supernatants were collected, cells were rinsed with PBS without Ca^2+^/Mg^2+^, and cells were harvested with trypsin LE medium (Lonza). Cells were pelleted and resuspended in FACS buffer before incubation for 10 min with PI (Miltenyi). Cells were analyzed using a Becton Dickinson FACSCalibur analyzer, and data were analyzed using Flowing software version 2.5.1.

### Cytokine Arrays and Pro-inflammatory Cytokine Panel

iPSC-MGLCs were seeded at 2 × 10^4^ cells/well in 96-well plates (Corning) in macrophage end-differentiation medium. The following day, cells were stimulated with 100 ng/mL LPS (Sigma) or 1 × 10^5^ apoptotic SH-SY5Y cells. Proteome Profiler Human XL Cytokine antibody array membranes or human cytokine array membranes (R&D Systems) were incubated with supernatants pooled from three independent experiments, according to *TREM2* genotype after basal, LPS, or apoptotic cell treatment, as per the manufacturer’s instructions. Data were analyzed using the Protein Array Analyzer Palette plug-in (ImageJ, USA), and plotted as a mean ± SEM after normalizing to membrane reference positive controls and intracellular protein concentrations. The levels of 10 cytokines were measured using a V-PLEX Proinflammatory Panel 1 Human Kit (Meso Scale Diagnostics, USA) on a SECTOR Imager 6000 analyzer (Meso Scale Diagnostics), according to the manufacturer’s instructions.

### Transfection with TREM2 siRNA

iPSC-MGLCs were seeded at 5 × 10^5^ cells/well in 6-well plates (Corning) for protein or siRNA extraction, or 1 × 10^5^ cells/well in 24-well plates (Corning) for phagocytosis assay or sTREM2 ELISA. Cells were transfected using SMARTpool ON-TARGETplus TREM2 (Dharmacon, USA) or non-targeting siRNA (Horizon Discovery, UK) and Lipofectamine RNAiMAX reagent (Thermo Fisher Scientific), according to the manufacturer’s instructions. Medium was changed before transfection. Cells were transfected 72 hr before assays or collection.

### Phagocytosis of pHrodo *E. coli or* pHrodo Zymosan

iPSC-MGLCs were seeded at 1 × 10^5^ cells/well in 24-well plates (Corning) 48 hr before the assay. As a negative control, cells were preincubated for 30 min with 10 μM cytochalasin D (Sigma). Cells were incubated with pHrodo *E. coli* (50 μg) or pHrodo zymosan (25 μg) (Life Technologies) particles for 2 hr. Cells were analyzed with a Becton Dickinson FACSCalibur flow cytometer, and results were analyzed with Flowing software (Cell Imaging Core of the Turku Centre for Biotechnology, flowingsoftware.btk.fi).

### Phagocytosis of Apoptotic Neuronal Cells

SH-SY5Y cells (a gift from Dr. R. de Silva, University College London Institute of Neurology [UCL ION]) were seeded at 4 × 10^5^ cells/well in 6-well plates in DMEM with 10% heat-inactivated bovine calf serum (Life Technologies) and 1% penicillin/streptomycin (Life Technologies). The following day, cells were loaded with Vybrant CM-Dil dye (1:200; Thermo Fisher Scientific) for 15 min, and the medium was changed to 500 μL PBS. Cells were irradiated with 500 J/m^2^ using a UV Crosslinker (Stratech, UK) and then incubated for 24 hr in normal DMEM. Apoptosis was confirmed using fluorescein isothiocyanate (FITC)-labeled annexin V (Miltenyi; see Figure S4B). Dye-labeled apoptotic SH-SY5Y cells were harvested in PBS without Ca^2+^/Mg^2+^, spun down, resuspended in macrophage end-differentiation medium, and counted. A total of 500,000 apoptotic SH-SY5Y cells were added to each well of the iPSC-MGLCs (seeded at 1 × 10^5^ cells/well in 24-well plates for 7–9 days and changed into 200 μL of fresh macrophage end-differentiation medium before the assay) for 2 hr. As a negative control, iPSC-MGLCs were pre-incubated for 30 min with 10 μM cytochalasin D (Sigma). iPSC-MGLCs were harvested with trypsin LE (Life Technologies) before being resuspended in FACS buffer and analyzed using a Becton Dickinson FACSCalibur analyzer. Data were analyzed using Flowing software version 2.5.1.

### Functional Migration Studies Using Scratch Assays and Trans-Wells

iPSC-MGLCs were scratched away in a line down the central area of the population with a sterile metal spatula, followed by washing to remove residual floating cells. Heat-shocked apoptotic SH-SY5Y cells were added to trans-wells (0.4 μm pore size) and placed over the scratched region for 24 hr. Trans-wells were removed before live cell staining with Hoechst 33342 for 45 min, followed by image capture on a Zeiss Axioskop 2 fluorescent microscope and image analysis using AxioVision 4.8 and ImageJ software.

### Statistical Analysis

Results were analyzed using Prism software (version 7, GraphPad, USA). One-way ANOVA with Dunnett’s correction for multiple comparisons was used unless otherwise indicated. Statistical analysis was performed on pooled controls and separate groups for each TREM2 heterozygous or homozygous mutation.

## Supplemental Information

Document S1. Supplemental Experimental Procedures and Figures S1–S7

Document S2. Article plus Supplemental Information
