# Supplementary material for: Human Induced Pluripotent Stem Cell-Derived Microglia-Like Cells Harboring TREM2 Missense Mutations Show Specific Deficits in Phagocytosis
Source: Cell Rep. 2018 Aug 28;24(9):2300–11. doi: 10.1016/j.celrep.2018.07.094 (PMC6130048; doi:10.1016/j.celrep.2018.07.094)
Supplement: Document S1. Supplemental Experimental Procedures and Figures S1–S7 [file mmc1.pdf]

Cell Reports, Volume *24*

## Supplemental Information

# Human Induced Pluripotent Stem Cell-Derived Microglia-Like Cells Harboring TREM2 Missense Mutations Show Specific Deficits in Phagocytosis

**Pablo Garcia-Reitboeck, Alexandra Phillips, Thomas M. Piers, Claudio Villegas-Llerena, Matt Butler, Anna Mallach, Celia Rodrigues, Charles E. Arber, Amanda Heslegrave, Henrik Zetterberg, Harald Neumann, Stephen Neame, Henry Houlden, John Hardy, and Jennifer M. Pocock**

**Supplementary Experimental Procedures**

**Supplementary text**

The karyotype of TREM2 mutant iPSC-lines was assessed with CNV analysis. Both clones of T66Mhet2 had a 47, XYY phenotype (Suppl. Fig1A). Both T66Mhom clones had large areas of loss of heterozygosity on chromosome 6 and 7 (suppl. Fig1B). Clone 3 of the T66Mhom iPSC line featured a heterozygous deletion of approximately 7MB on chromosome 3, starting at position 63348134 and ending at 69934331 (suppl. Fig. 1C). Karyotype analysis of T66Mhom clone 3 was normal (suppl. Fig. 1D). The CNV analysis data for all TREM2 mutant lines can be seen in suppl. Table 1.

**Generation of human iPSC-derived microglia-like cells**

TREM2 mutant primary fibroblast lines were generated from 4mm skin punch biopsies, which were obtained under informed consent. Ethical permission for this study was obtained from the National Hospital for Neurology and Neurosurgery and the Institute of Neurology joint research ethics committee (study reference 09/H0716/64 for collection of W50C mutant fibroblasts) or approved by the Ethics Committee of Istanbul Faculty of Medicine, Istanbul University (for collection of T66M mutant fibroblasts to Dr Ebba Lohmann). iPSC were generated from fibroblast cultures using 4 factor Sendai virus reprogramming and characterised by RT-PCR for plasmid integration, immunofluorescence for expression of pluripotent markers, and 3-germ layer differentiation and at the NIHR Cambridge Biomedical Research Centre (BRC), human Induced Pluripotent Stem Cells core facility (see Brownjohn et al. 2018). Karyotypic integrity was assessed by CNV analysis using OmniExpress-24 chips run on the Illumina Infinum platform. CNVs were checked visually on GenomeStudio software. The following control iPSC lines were used: CTRL1 (kindly provided by Dr Selina Wray, UCL Institute of Neurology), ND (called CTRL2 in this paper, from Coriell Institute of Medical Research Biorepository, kindly provided by Dr Rickie Patani, UCL Institute of Neurology), SBAD03 (called CTRL3, from Stembancc), CTRL4 (SFC840, from Stembancc). iPSC colonies were maintained in E8 medium (Life Technologies), and split every 4-5 days using Versene (Lonza). iPSC colonies were differentiated into iPSC-macrophages within 5 passages. Human iPSC-derived microglia-like cells (iPSC-MGLC) were generated using recently published protocols (van Wilgenburg et al. 2013), with minor modifications. To generate embryoid bodies at day 0 in vitro (D0), iPSC colonies at 60-80% confluence were dissociated for 4 min with TrypLE (Life Technologies), collected in 10 x the volume of PBS without $Ca^{2+}/Mg^{2+}$, pelleted for 3 min at 300 g and resuspended in 1 ml EBDiff medium (E8 medium with 10 µmol ROCK inhibitor (Cambridge Bioscience), 50 ng/ml BMP-4 (Preprotech), 50 ng/ml VEGF, (Preprotech), 20 ng/ml SCF (Miltenyi)). Cells were counted, resuspended in EBDiff medium at $10^5$ cells/ml and 100 µl /well (i.e. $10^4$ cells/well) were distributed into low adherence 96 well plates (Corning 7007), centrifuged at 800rpm (115g) for 3 min, and gently

transferred to a tissue culture incubator at 37ºC with 5% $CO_2$. On D2, 50 µl of EBDiff medium/well were gently added to each well. On D4, embryoid bodies were collected with a P1000 Gilson pipette, transferred into a 15 ml Eppendorf tube, and left to settle at the bottom. The old medium was aspirated and myeloid differentiation medium (X-vivo 15 medium (Lonza) supplemented with 1% Glutamax (Life Technologies), 100U Penicillin/Streptomycin (Life Technologies) and 50 µM mercaptoethanol (Life Technologies), with 100 ng/ml MCSF (Peprotech) and 25 ng/ml IL-3 (Cell Guidance Systems) added. Approximately 150 embryoid bodies were transferred into a 175 $cm^2$ flask. Myeloid differentiation medium was changed every 5-7 days, making sure that the medium did not become acidotic. After 3-4 weeks, iPSC-MGLC were harvested once a week by replacing 2/3 of the medium and filtering the cells through a 40 micron filter (BD Bioscience). Harvested IPSC-MGLC were further differentiated in X-Vivo 15 medium with 1% Glutamax, 100 U Penicillin/Streptomycin and 100 ng/ml MCSF. iPSC-MGLC were used for experiments between day 2 and day 7 post harvesting.

**TREM2 genotyping by Sanger sequencing**

Genomic DNA was extracted from approximately 1 million cells using DNeasy Blood & Tissue Kits (QIAGEN), following the manufacturer's instructions. DNA was diluted with nuclease free water to 25ng/µl for TREM2 genotyping. TREM2 genotyping was carried out by PCR and Sanger sequence analysis of the complete second exon of the TREM2 gene. The amplification reaction was performed in a volume of 50µl, which contained 2XKAPA HiFi HotStart ReadyMix (Kapa Biosystems), 0.3µM of each primer (Forward primer: 5'-AGTGGGTGGTTCTGCACAC-3' and Reverse primer: 5'-TCCTTCAGGGCAGGATTTTT-3'), and 50ng genomic DNA. PCR cycling conditions were as follows: Initial denaturation at 95°C for 3 minutes followed by 35 cycles with denaturation at 98°C for 20 sec, annealing at 62°C for 15 sec, extension at 72°C for 30 sec; then a final extension at 72°C for 1 minute. PCR were subsequently visualized on a 1.5% agarose gel to verify the size of the amplicon (533 bp). Finally PCR products were purified using the QIAquick PCR Purification Kit (QIAGEN) and then sequenced commercially (Source Bioscience, Nottingham, UK). Alignment of sequences and analysis was carried out using SnapGene software (GSL Biotech, version 3.3.3).

**Isolation of human blood derived-monocytes**

Phlebotomy was performed and blood collected into heparinised tubes. This study was approved by University College London (UCL)/UCL Hospitals Joint Research Ethics Committee (Project ID: 7451/001) and all subjects gave informed written consent. Plasma was separated by incubating the heparinized blood with 3% dextran 500 (Sigma) in an upright position for 60 minutes at room temperature. The leukocytes were pelleted from plasma by centrifugation for 6 minutes at

1600 rpm (461g). The cells were resuspended in PBS, layered over Histopaque (Sigma) and centrifuged with low acceleration and brake for 20 minutes at 720g. The resulting peripheral blood mononuclear cell (PBMC) layer was collected and CD14 Microbeads (Miltenyi Biotec) were used to isolate the monocytes, according to the manufacturer's instructions. Blood-derived monocytes were cultured at a density of $5x10^5$ per well in X-Vivo medium (Lonza) supplemented with 100U/ml penicillin/streptomycin (Life Technologies), 1% Glutamax (Life Technologies) and matured into macrophages through continuous culture in 100 ng/ml MCSF (Preprotech) for 6 days.

**Quantitative PCR**

Cells were lysed and homogenised using RLT buffer (QIAGEN) and total RNA was extracted from cells using an RNeasy minikit (QIAGEN), with an on-column DNase digestion step using RNase-free DNase set (QIAGEN). Complementary DNAs were generated using the High-Capacity RNA-cDNA kit (Life Technologies), according to the manufacturer's instructions. Quantitative PCR were conducted using TaqMan Universal Mastermix II (ThermoFisher) in the Stratagene Mx3000p qPCR system and MxPro qPCR software. Expression was normalised to GAPDH. The following primers were used (all purchased from Applied Biosystems):

| **Gene name** | **Primer ID** |
|---|---|
| APOE | Hs00171168_m1 |
| C1QA | Hs00381122_m1 |
| CD33 | Hs01076281_m1 |
| CSF1R | Hs00911250_m1 |
| GAPDH | Hs0278991_g1 |
| GPR34 | Hs00758331_m1 |
| PROS1 | Hs00165590_m1 |
| SPI1 | Hs02786711_m1 |
| P2Y12 | Hs00224470_m1 |
| TGFB1 | Hs00998133_m1 |
| TMEM119 | Hs01938722_u1 |
| TREM2 | Hs00219132_m1 |

| | |
|---|---|
| TYROBP | Hs00182426_m1 |

**Western blotting**

Cellular protein was solubilised using a modified RIPA buffer (25mM TRIS, pH 7.4, 50mM NaCl, 0.5% NP-40, 0.25% Na-Deoxycholate, 1mM EDTA) with protease/phosphatase inhibitors (Life Technologies), and denatured in 4 X sample buffer + 10% Dithiothreitol (DTT). Samples were resolved on sodium dodecyl sulfate (SDS)-polyacrylamide gels, and transferred to 0.2µm nitrocellulose membranes (BioRad). Primary antibodies against goat anti-TREM2 (AF1828, 1:500, R & D Systems; Cell Signaling), rabbit anti-DAP12 (MAB5240, 1:500, R & D Systems), rabbit anti-TREM2 (D8I4C, 1:1000, Cell Signaling Technology) and anti-β-actin (clone AC15, 1:15000, Sigma) were purchased from commercial sources. Immunoreactive bands were probed with goat/donkey anti-rabbit/mouse/goat IgG (H+L) IRDye 680RD or IRDye 800CW (1:10 000, LiCOR) secondary antibodies for 1h and developed using the Odyssey detection system (LiCOR). Optical densities (O.D.) of immunoreactivity were quantified using Odyssey version 3.0 software.

**Fluorescence-activated cell sorting**

At least $1x10^5$ – $1x10^6$ cells per iPSC line were used for FACS analysis. Cells were harvested by rinsing cells in PBS withouy $Ca^{2+}/Mg^{2+}$ and incubating for 10 minutes in PBS without $Ca^{2+}/ Mg^{2+}$. Cells were washed 2x with FACS buffer (PBS + 0.5% bovine serum albumin (BSA, Sigma) + 2mM EDTA), incubated with FC-block (Miltenyi) and primary antibodies (1:10) or isotype control antibodies for 1h at 4ºC, washed 3x with FACS buffer and analysed using a BD FACS Calibur analyser. Results were analysed using FlowJo software (v8.8.7, Tree Star). The following antibodies were used for FACS: rat anti-CD11B-APC (ICRF44, Insight Biotechnologies), mouse anti-CD45-FITC (130-080-202, Miltenyi), anti-CSF1R FITC (FAB329F-100, R&D systems), mouse anti-CD33 FITC (130-098-695, Miltenyi).The following isotype antibodies were used: mouse IgG2a-FITC (Miltenyi), mouse IgG1-FITC (130-092-213, Miltenyi), rat IgG2-APC (130-103-085, Miltenyi).

**Immunocytochemistry**

Cells were seeded at $2.5x10^4$ cells/well, fixed in 4% paraformaldehyde in PBS, washed 1x with PBS, permeabilised with 0.2% Triton X in PBS for 10 minutes at room temperature (RT), blocked in 5% donkey (TREM2 AF1828 antibody) or 5% goat (all other antibodies) serum in PBS for 1h at RT, and incubated in 1% donkey or goat serum in PBS with primary antibody for 2h at RT. For double-immunofluorescence with anti-TREM2 and Golgin antibodies, cells were

blocked with 3% bovine serum albumin (BSA) and incubated with 1% BSA in PBS with primary and secondary antibodies, Cells were washed 3x with PBS and incubated for 1h with Alexa Fluor secondary antibodies in 1% serum PBS. Images were taken using a Leica DM550 microscope and LAS AF software (Leica, V2.6.3). For live cell imaging of pHrodo particles, images were taken using a Zeiss Axioskop 2 microscope and Axiovison software (Zeiss, v4.8). Confocal images were taken with a Zeiss LSM 710 confocal microscope using Zen software (Zeiss, Version 2012), and images processed with Image J1.5 (www.imagej.nih.gov/ij). The following antibodies were used for immunocytochemistry: primary antibodies: mouse anti-CD68 (1:100, DAKO), rabbit anti-iba1 (1:500, WAKO), goat anti-TREM2 AF1828 (10µg/ml, R&D systems), rabbit-anti-TREM2 (D814C, 1:400, Cell Signaling Technology), rabbit-anti-Golgin (1:100, Life Technologies). The following secondary antibodies were used: Alexa Fluor donkey anti-goat 568, goat-anti-rabbit 568, goat-anti mouse 488 (all 1:500, Life Technologies).

**sTREM2 ELISA**

Quantification of sTREM2 from cell culture supernatants was performed using an in house-generated ELISA. MaxiSORP 96 well plates (Nunc) were coated with 1µg/ml of a rat anti-mouse/human monoclonal antibody (R&D Systems; clone 237920) overnight at 4°C. Plates were washed with 1x PBS/0.1% Tween-20 (PBS-T) then blocked with 1% bovine serum albumin (BSA) in 0.1% Tween 20 in PBS (1% BSA/PBS-T; pH 7.4) for 45 minutes at room temperature (RT) with gentle shaking, followed by 3 washes with PBS-T. Cell culture supernatant samples and standards (Recombinant human TREM2-His; Life Technologies; 0 - 20ng/ml in 1% BSA/PBS-T) were then incubated for 2h at RT with gentle shaking, followed by 3 washes with PBS-T. For the detection of bound human sTREM2 from the cell culture supernatants, plates were incubated for 1.5h at RT with biotinylated polyclonal goat anti-human TREM2 capture antibody (0.1µg/ml; R&D Systems) diluted in 1% BSA/PBS-T. Plates were subsequently washed 4 times with PBS-T, followed by incubation with a streptavidin-HRP (Invitrogen; 0.1µg/ml) diluted in PBS-T for 45 minutes at RT with gentle shaking. Plates were washed 3 times with PBS-T followed by the addition of a chromogenic substrate solution (TMB) in the dark. The reaction was terminated with the addition of stop solution (0.16M $H_2SO_4$) and absorbance was read at 450 nm (Tecan Genios). To further verify sTREM2 secretion a second method was employed, adapted from Kleinberger et al., (2014). Streptavidin-coated 96-well plates (Meso-Scale discovery (MSD)) were blocked overnight at 4°C in block buffer (0.5% bovine serum albumin (BSA) and 0.05% Tween 20 in PBS (pH 7.4). The plates were next incubated with the biotinylated polyclonal goat anti-human TREM2 capture antibody (0.25µg/ml R&D Systems BAF1828) diluted in block buffer for 1h at room temperature. They were subsequently washed four times with wash buffer (0.05% Tween 20 in PBS) and incubated for 2h at RT with the undiluted cell culture media samples or a standard

curve constructed from recombinant human TREM2 protein (4000 -62.5pg/ml Sino Biological Inc 11084-H08H) diluted in assay buffer (0.25% BSA and 0.05% Tween 20 in PBS (pH=7.4). Plates were again washed three times with wash buffer before incubation for 1h at RT with the detector antibody monoclonal mouse anti-human TREM2 antibody (1µg/ml Santa Cruz Biotechnology; B-3, sc373828). After three additional washing steps, plates were incubated with the secondary antibody (SULFO-TAG–labeled anti-mouse secondary antibody, MSD) and incubated for 1h in the dark. Lastly, plates were washed three times with wash buffer followed by two washing steps in PBS alone. The electrochemical signal was developed by adding MSD Read buffer (1 in 2) and the light emission measured using the MSD SECTOR Imager 6000. The concentration of sTREM2 was calculated using a five-parameter logistic curve fitting method with the MSD Workbench software package. Intra-assay cvs were < 10%, and all samples were measured on the same day using the same reagents.

**pHrodo *E.coli* and pHrodo zymosan phagocytosis assay**

iPSC-MGLC were seeded at $10^5$ cells /well in 24 well plates (Corning) 48h before the assay. As a negative control, cells were preincubated for 30 minutes with 10µM Cytochalasin D (Sigma). pHrodo green *E.coli* particles (Life Technologies) were diluted in PBS without $Ca^{2+}/Mg^{2+}$ to a concentration of 1mg/ml, and 50µg were added per well in 200µl of macrophage end-differentiation medium. PHrodo zymosan particles (Life Technologies) were diluted in PBS without $Ca^{2+}/Mg^{2+}$ to a concentration of 0.5mg/ml, and 25µg were added per well in 200µl of macrophage end-differentiation medium. Cells were incubated with pHrodo *E.coli* or pHrodo zymosan particles for 2h, rinsed and harvested with PBS without $Ca^{2+}/Mg^{2+}$ and washed twice with FACS buffer (PBS + 0.1% BSA, 2mM EDTA, 0.05% sodium azide). Cells were analysed with a BD FACS Calibur flow cytometer and results analysed with Flowing software (Cell Imaging Core of the Turku Centre for Biotechnology, flowingsoftware.btk.fi). Mean fluorescence intensities were calculated for each sample from 3 independent experiments, normalised to one control line and pooled for statistical analysis.

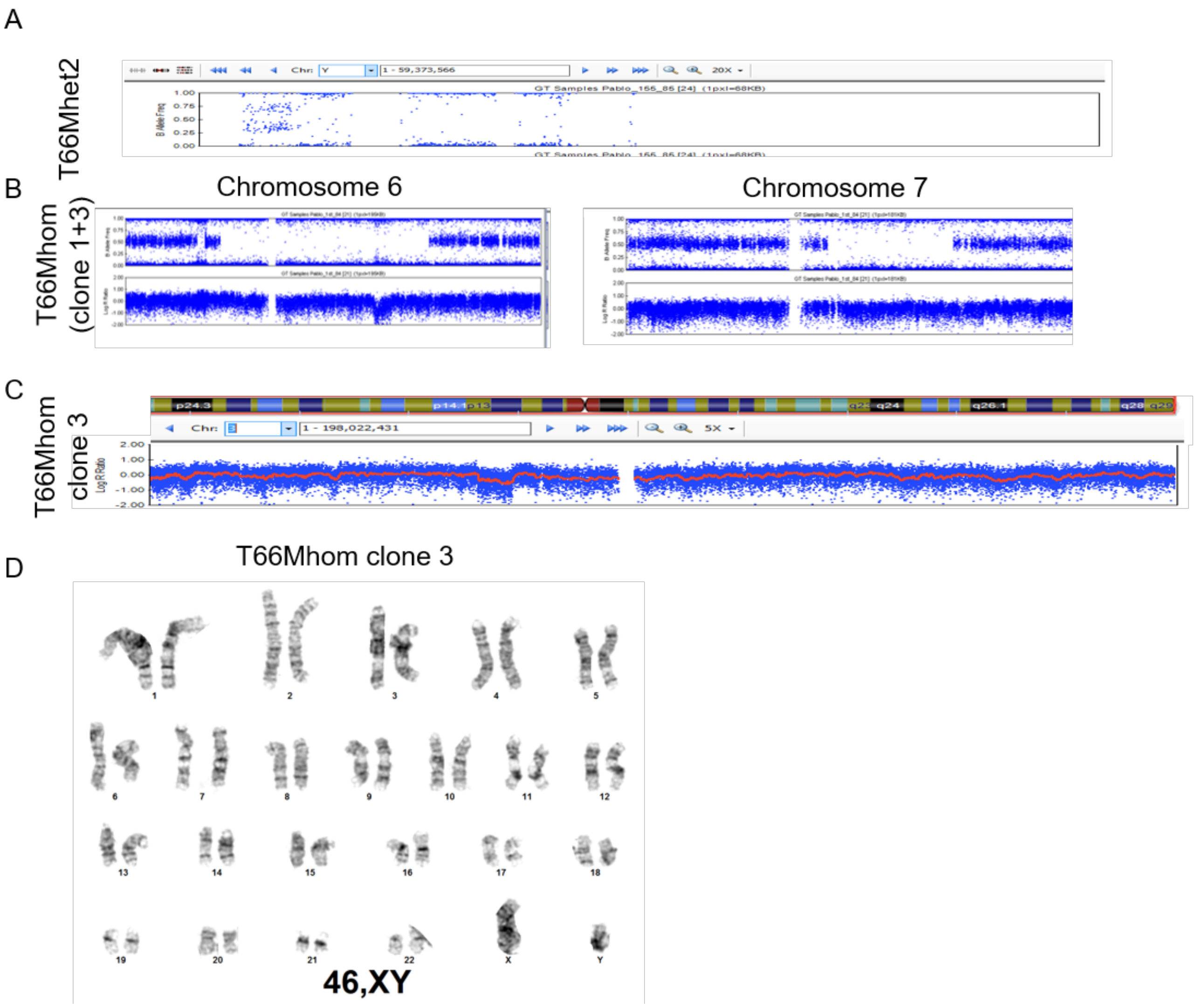


**Figure S1. Karyotyping and CNV analysis of TREM2 mutant iPSC. Related to Figure 2.**

A 47, XYY karyotype was identified for both clones of T66Mhet2 iPSC line (A, analysis of one clone shown). Areas of prominent loss of heterozygosity were identified on chromosome 6 and 7 for clones of the T66Mhom line (B). The T66Mhom clone 3 iPSC line featured a heterozygous deletion of approximately 7MB on chromosome 3, starting at position 63348134 and ending at 69934331 (C). Karyotype analysis of T66Mhom was normal (D).

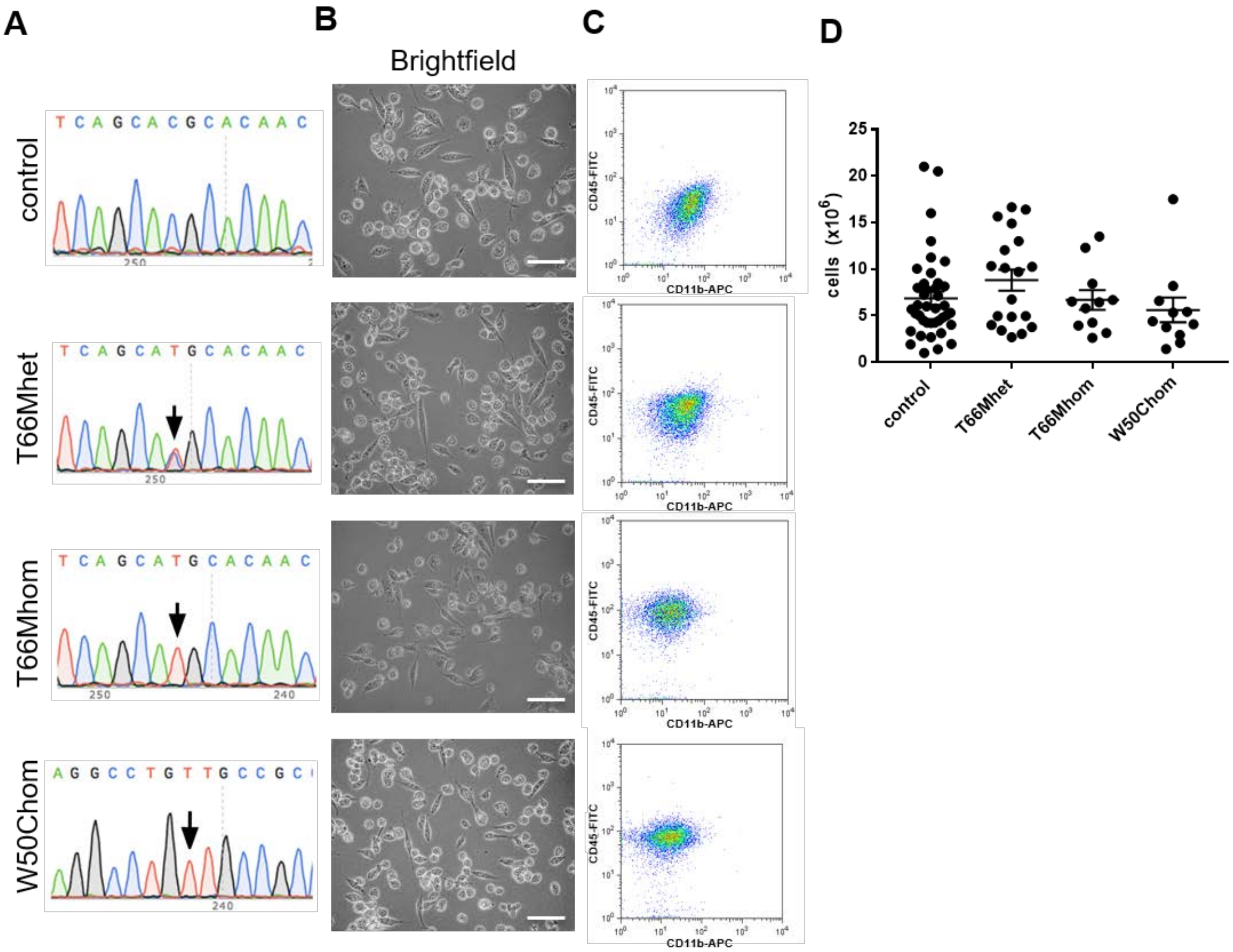


**Figure S2. Genotype characterisation. Related to Figures 1 and 2.**

Sanger sequencing of T66M heterozygous, T66M and W50C homozygous mutations in TREM2 variant iPSC-MGLC (arrows, A, one representative control and T66Mhet iPSC-MGLC shown). Brightfield microscopy of control and TREM2 variant iPSC-MGLC, scale bar=100 μm (B). FACS analysis of the macrophage markers CD45 and CD11b in control and TREM2 variant iPSC-MGLC (C). The weekly iPSC-MGLC yield between control and TREM2 variant iPSC (D). One representative control and T66Mhet line are shown in Figures 2A-C. Data are represented as mean ± SEM.

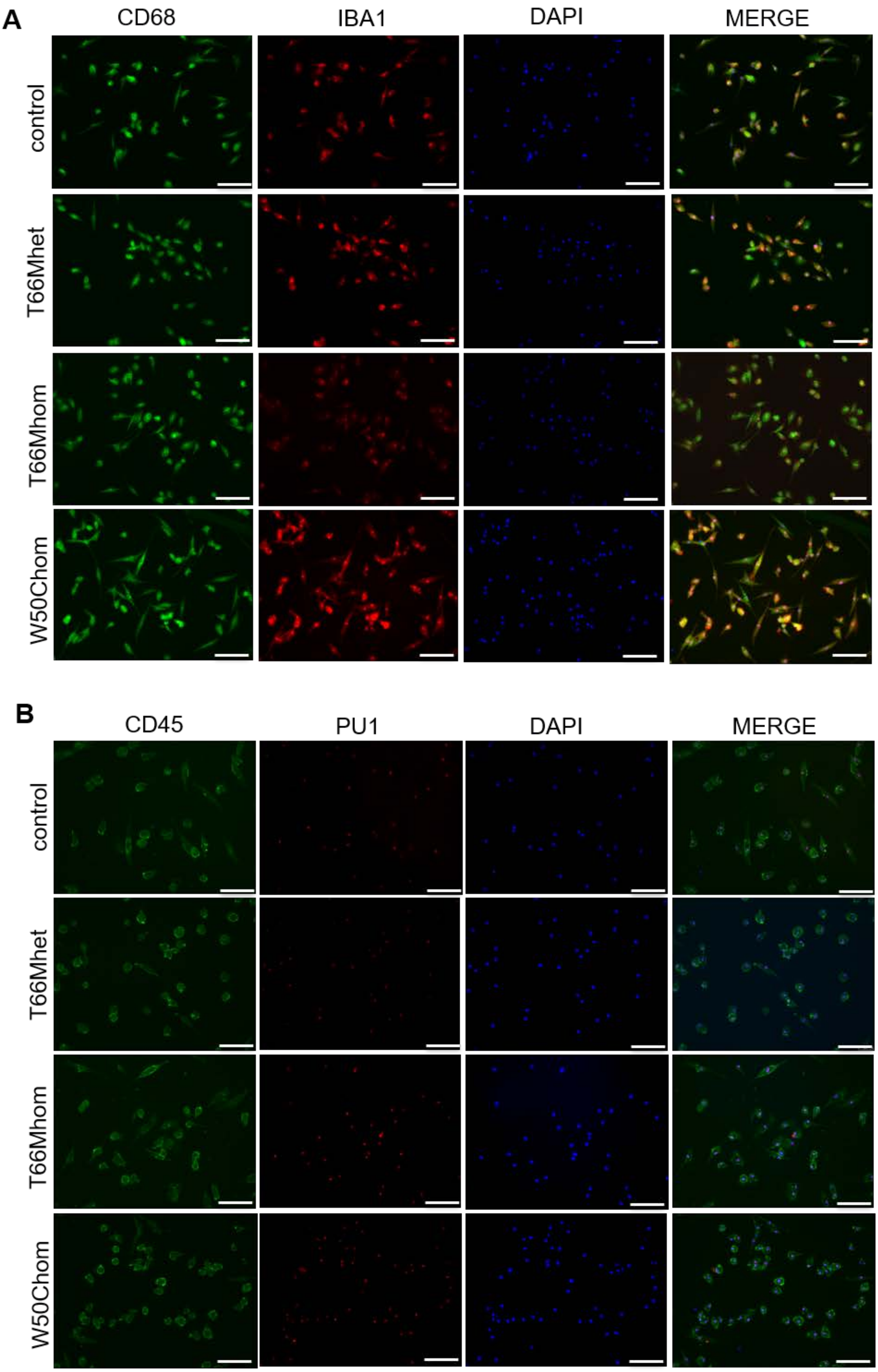
A
CD68
IBA1
DAPI
MERGE
control
T66Mhet
T66Mhom
W50Chom
B
CD45
PU1
DAPI
MERGE
control
T66Mhet
T66Mhom
W50Chom

**Figure S3. Expression of macrophage markers in control and TREM2 variant iPSC-MGLC. Related to Figures 1 and 2.**

Double-immunofluorescence staining for the macrophage markers CD68 and Iba1 (A) and CD45 and Pu1 (B) in control, T66Mhet, T66Mhom, and W50Chom iPSC-MGLC. Scale bar: 100μm

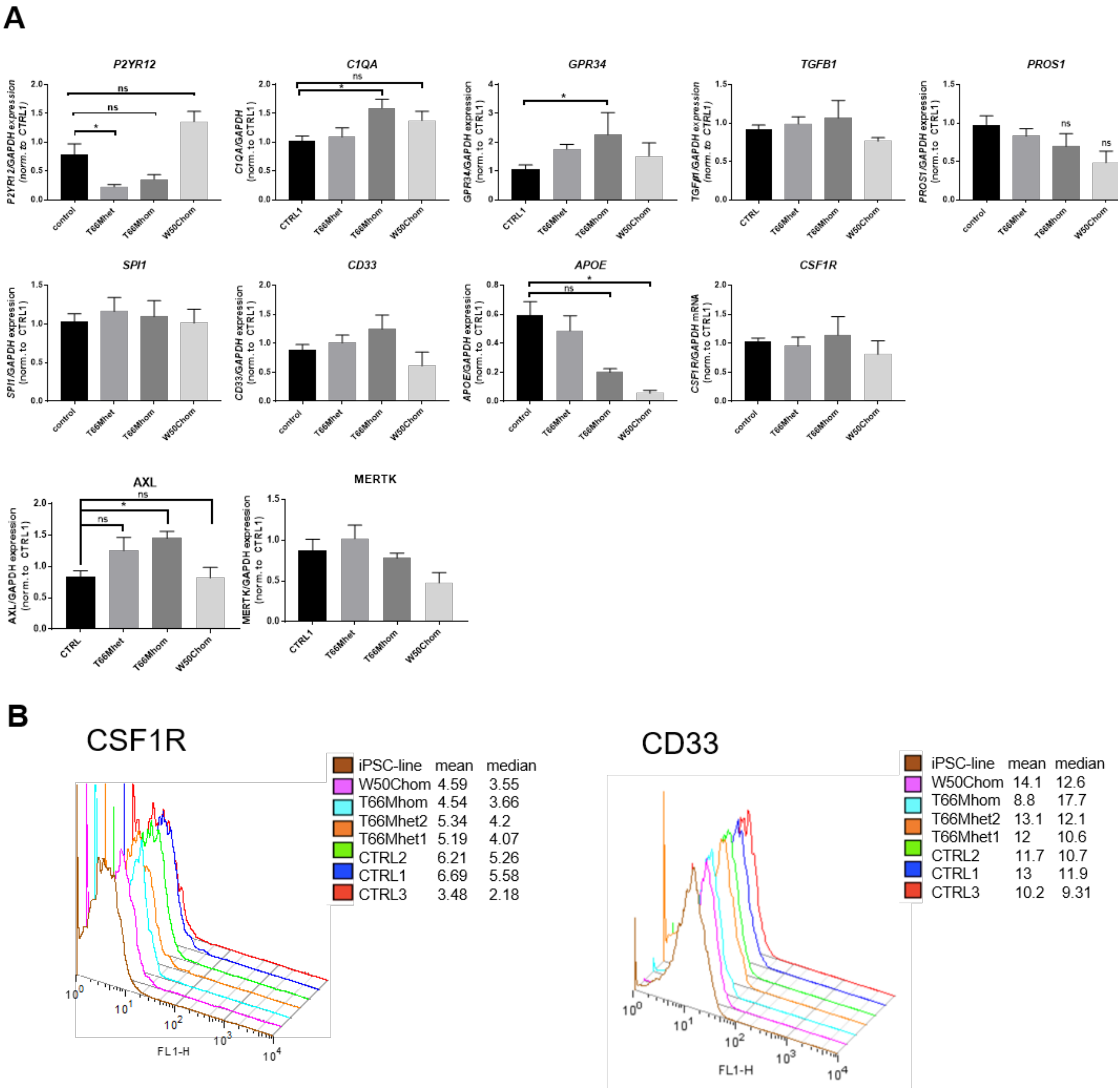


**Figure S4. Microglial gene and protein signature analysis. Related to Figures 1 and 2.**

Comparison of microglial gene expression in control and TREM2 variant iPSC-MGLC (A). (* $p<0.05$). FACS analysis of surface expression of CSF1R or CD33 (B) in TREM2 variant iPSC-MGLC compared with controls; n=3, the table displays mean and median fluorescent intensities.

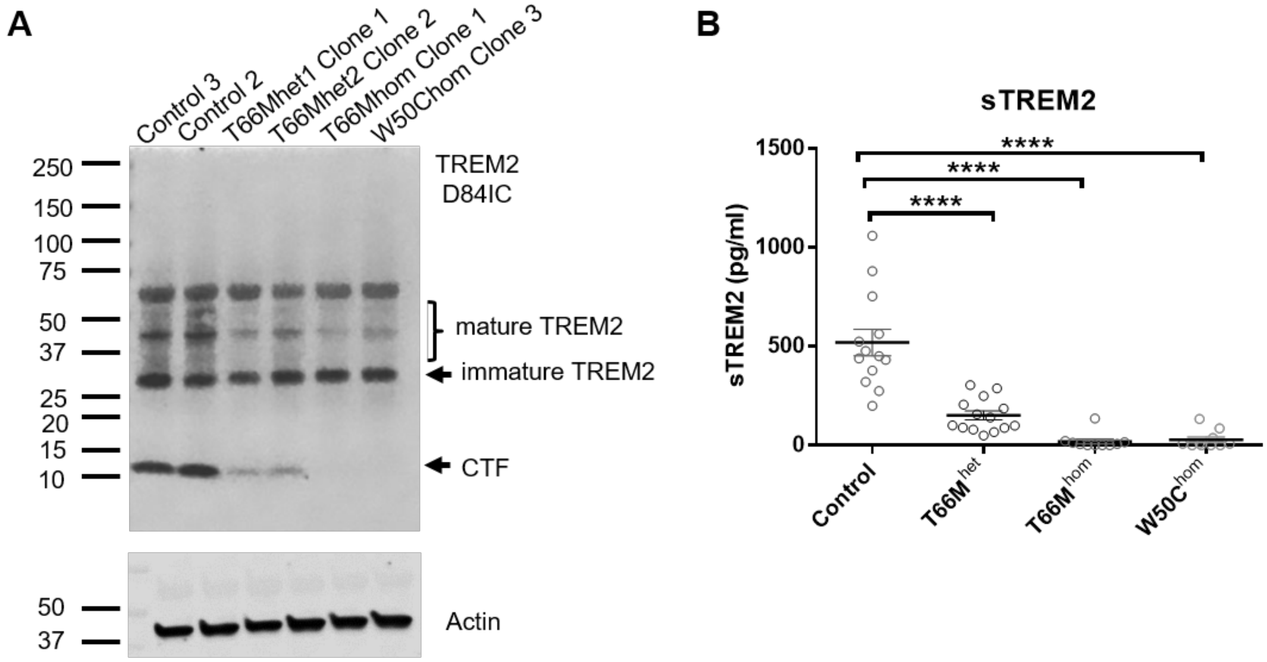


**Figure S5. Protein analysis of additional clones. Related to Figure 2.**

WB using D8I4C antibody against TREM2 on additional TREM2 variant iPSC clones (1 additional clones per TREM2 variant line) (A). sTREM2 ELISA of additional TREM2 variant iPSC clones (1 additional clone per TREM2 mutant line) (B, **** $p<0.0001$).

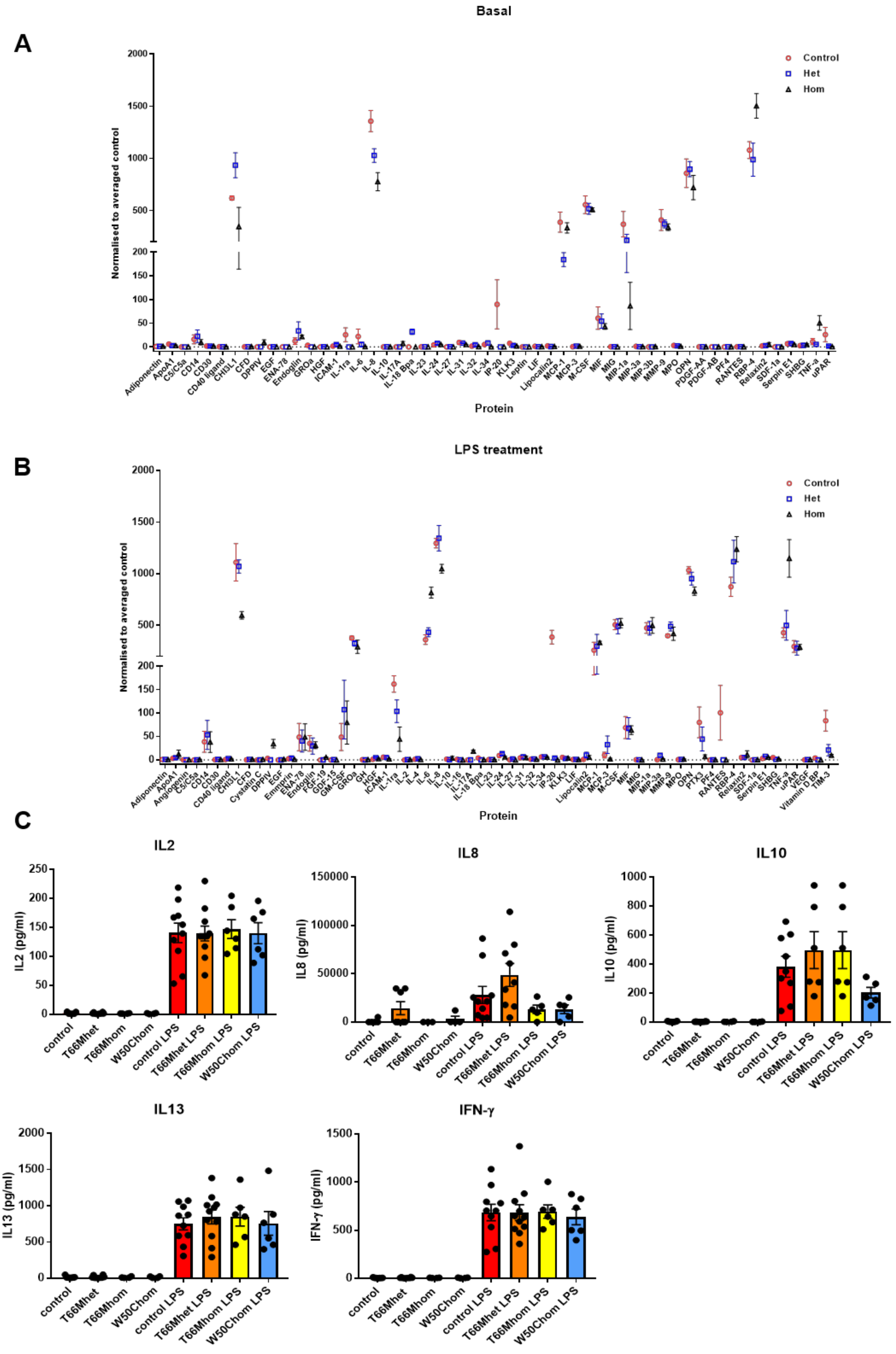
A
Basal
Normalised to averaged control
Protein
Control
Het
Hom
Adiponectin ApoA1 C5/C5a CD14 CD30 CD40 ligand CHI3L1 CFD DPPIV EGF ENA-78 Endoglin GROa HGF ICAM-1 IL-1ra IL-6 IL-8 IL-10 IL-17A IL-18 Bpa IL-23 IL-24 IL-27 IL-31 IL-32 IL-34 IP-20 KLK3 Leptin LIF Lipocalin2 MCP-1 MCP-3 M-CSF MIF MIG MIP-1a MIP-3a MIP-3b MMP-9 MPO OPN PDGF-AA PDGF-AB PF4 RANTES RBP-4 Relaxin2 SDF-1a Serpin E1 SHBG TNF-a uPAR
B
LPS treatment
Normalised to averaged control
Protein
Control
Het
Hom
C
IL2
IL2 (pg/ml)
IL8
IL8 (pg/ml)
IL10
IL10 (pg/ml)
IL13
IL13 (pg/ml)
IFN-γ
IFN-γ (pg/ml)
control
T66Mhet
T66Mhom
W50Chom
control LPS
T66Mhet LPS
T66Mhom LPS
W50Chom LPS

**Figure S6. Complete proteome profiler analysis and other MSD cytokine profiles. Related to Figure 4.**

Complete cytokine profiles using Human XL cytokine array on cell culture supernatants from one control line, one T66Mhet line, one T66Mhom and W50Chom iPSC-MGLC under basal (A; untreated) conditions and following stimulation with LPS, 100 ng/ml after 18 h (B). Levels of remaining tested cytokines: IL2, IL8, IL10, IL13, and IFNγ measured on the MSD platform using a V-PLEX Pro-inflammatory Panel 1 (C). (n=2 for untreated, n=3 for LPS-treated samples in 4 control iPSC lines, and 2 clones per TREM2 variant IPSC-lines were analysed).

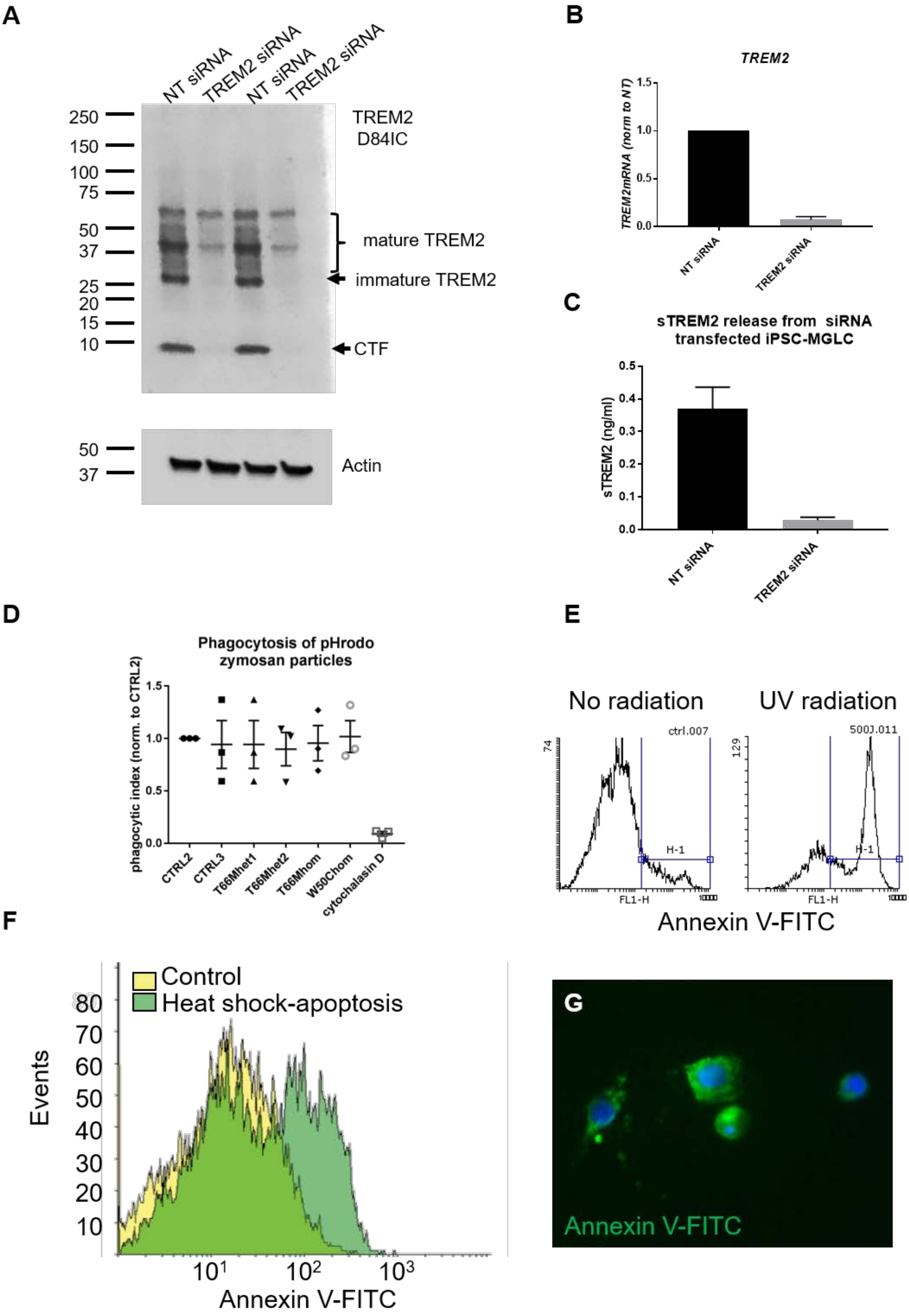
A
NT siRNA
TREM2 siRNA
NT siRNA
TREM2 siRNA
250
150
100
75
50
37
25
20
15
10
TREM2
D84IC
mature TREM2
immature TREM2
CTF
50
37
Actin
B
TREM2
TREM2mRNA (norm to NT)
1.5
1.0
0.5
0.0
NT siRNA
TREM2 siRNA
C
sTREM2 release from siRNA transfected iPSC-MGLC
sTREM2 (ng/ml)
0.5
0.4
0.3
0.2
0.1
0.0
NT siRNA
TREM2 siRNA
D
Phagocytosis of pHrodo zymosan particles
phagocytic index (norm. to CTRL2)
1.5
1.0
0.5
0.0
CTRL2
CTRL3
T66Mhet1
T66Mhet2
T66Mhom
W50Chom
cytochalasin D
E
No radiation
UV radiation
ctrl.007
500J.011
74
129
H-1
FL1-H
Annexin V-FITC
F
Control
Heat shock-apoptosis
Events
80
70
60
50
40
30
20
10
10^1
10^2
10^3
Annexin V-FITC
G
Annexin V-FITC

**Figure S7. Characterisation of siRNA knockdown, pHrodo zymosan phagocytosis, and generation of apoptotic SH-SY5Y cells. Related to Figures 5 and 6.**

Reduction of TREM2 protein (A), TREM2 mRNA (B), and TREM2 secretion (C) using TREM2 siRNA compared with non-targeting siRNA confirmed by WB, qPCR, or sTREM2 ELISA, respectively (n=3 for qPCR, n=2 for WB and sTREM2 ELISA). Phagocytosis of pHrodo zymosan particles in variant *TREM2* iPSC-MGLC and controls (D, n=3). Cytochalasin D was used as negative control. Data are presented as mean ± SEM. Fluorescent Annexin V staining to demonstrate apoptosis of SH-SY5Y cells following UV radiation and further incubation over a 24h period, compared with un-treated cells (E). FACS analysis for Annexin V staining in SH-SY5Y controls (non-heat shocked, yellow filled graph) versus heat-shock treated (green filled graph) (F). Annexin V immunocytochemistry in heat-shocked SH-SY5Y cells imaged by fluorescence microscopy (G).
