# Supplementary material for: Human Induced Pluripotent Stem Cell-Derived Microglia-Like Cells Harboring TREM2 Missense Mutations Show Specific Deficits in Phagocytosis
Source: Cell Rep. 2018 Aug 28;24(9):2300–11. doi: 10.1016/j.celrep.2018.07.094 (PMC6130048; doi:10.1016/j.celrep.2018.07.094)
Supplement: Document S2. Article plus Supplemental Information [file mmc2.pdf]

Cell Reports

Article

Human Induced Pluripotent Stem Cell-Derived Microglia-Like Cells Harboring TREM2 Missense Mutations Show Specific Deficits in Phagocytosis

Graphical Abstract

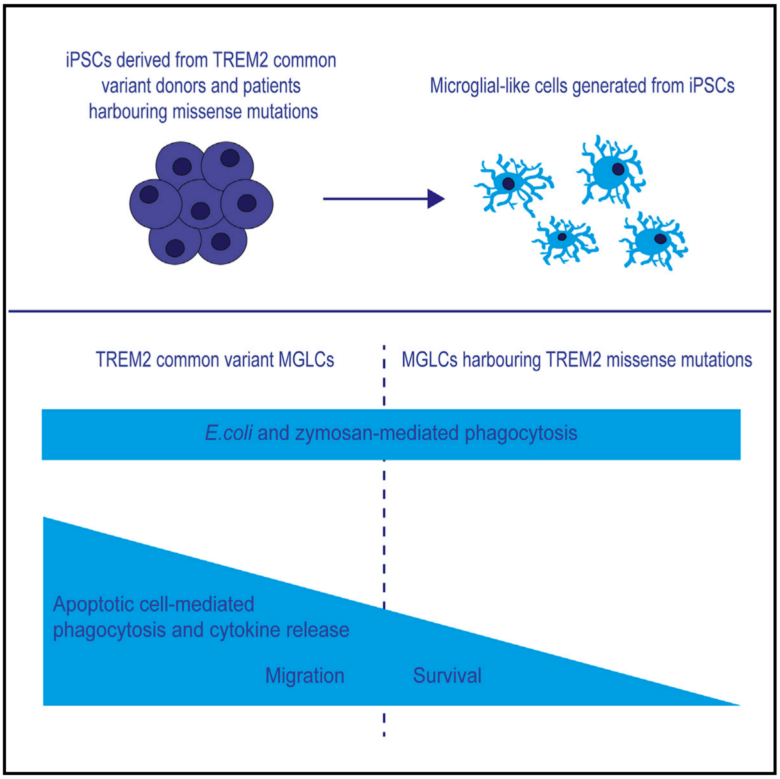

Authors

Pablo Garcia-Reitboeck, Alexandra Phillips, Thomas M. Piers, ..., Henry Houlden, John Hardy, Jennifer M. Pocock

Correspondence

j.pocock@ucl.ac.uk

In Brief

Garcia-Reitboeck et al. describe the generation of human induced pluripotent stem cell-derived microglia-like cells from patients with early-onset dementia caused by variants in the immune receptor gene *TREM2.* They observed functional deficits in TREM2 variant cells, including reduced soluble TREM2 secretion, selectively reduced phagocytosis of apoptotic neuronal cells, and a deficit in migratory capacity.

Highlights

- Generated human microglia-like cells from *TREM2* T66M and W50C mutation carriers
- Heterozygous and homozygous *TREM2* variants impair shedding of soluble TREM2
- Cytokine secretion not altered in *TREM2* variants following LPS exposure
- Substrate specific impairment of microglial function observed in *TREM2* variants

Garcia-Reitboeck et al., 2018, Cell Reports *24*, 2300–2311
August 28, 2018 © 2018 The Author(s).
https://doi.org/10.1016/j.celrep.2018.07.094

CellPress

OPEN ACCESS
CellPress

Cell Reports
Article

Human Induced Pluripotent Stem Cell-Derived Microglia-Like Cells Harboring TREM2 Missense Mutations Show Specific Deficits in Phagocytosis

Pablo Garcia-Reitboeck,[1,2,7] Alexandra Phillips,[1,7] Thomas M. Piers,[1,3,7] Claudio Villegas-Llerena,[2] Matt Butler,[1,3] Anna Mallach,[1] Celia Rodrigues,[2] Charles E. Arber,[2] Amanda Heslegrave,[2] Henrik Zetterberg,[2,4] Harald Neumann,[5] Stephen Neame,[6] Henry Houlden,[2] John Hardy,[2] and Jennifer M. Pocock[1,8,*]
[1]Department of Neuroinflammation, University College London Institute of Neurology, London WC1N 1PJ, UK
[2]Department of Molecular Neuroscience, University College London Institute of Neurology, London WC1N 1PJ, UK
[3]Eisai:UCL Therapeutic Innovation Group, University College London Institute of Neurology, London WC1N 1PJ, UK
[4]Department of Psychiatry and Neurochemistry, Institute of Neuroscience and Physiology, The Sahlgrenska Academy at the University of Gothenburg, Gothenburg 41345, Sweden
[5]Neural Regeneration Group, Institute of Reconstructive Neurobiology, University of Bonn, Bonn 53127, Germany
[6]Neurology Business Group, Hatfield Research Laboratories, Neurology Innovation Centre, Eisai Limited, Hatfield, AL10 9SN, UK
[7]These authors contributed equally
[8]Lead Contact
*Correspondence: j.pocock@ucl.ac.uk
https://doi.org/10.1016/j.celrep.2018.07.094

SUMMARY

Dysfunction of microglia, the brain's immune cells, is linked to neurodegeneration. Homozygous missense mutations in *TREM2* cause Nasu-Hakola disease (NHD), an early-onset dementia. To study the consequences of these *TREM2* variants, we generated induced pluripotent stem cell-derived microglia-like cells (iPSC-MGLCs) from patients with NHD caused by homozygous T66M or W50C missense mutations. iPSC-MGLCs expressed microglial markers and secreted higher levels of TREM2 than primary macrophages. TREM2 expression and secretion were reduced in variant lines. LPS-mediated cytokine secretion was comparable between control and TREM2 variant iPSC-MGLCs, whereas survival was markedly reduced in cells harboring missense mutations when compared with controls. Furthermore, TREM2 missense mutations caused a marked impairment in the phagocytosis of apoptotic bodies, but not in *Escherichia coli* or zymosan substrates. Coupled with changes in apoptotic cell-induced cytokine release and migration, these data identify specific deficits in the ability of iPSC-MGLCs harboring *TREM2* missense mutations to respond to specific pathogenic signals.

INTRODUCTION

Microglia, the resident macrophages of the brain, fulfill a variety of functions in the development and maintenance of the CNS, including synaptic pruning, surveillance, response to noxious and infectious agents, and tissue repair (Wolf et al., 2017). A number of microglial genes have been identified as genetic risk factors for Alzheimer disease (AD) (Villegas-Llerena et al., 2016). The immune receptor triggering receptor expressed on myeloid cells-2 (TREM2) was recently identified as a genetic risk factor for AD (Guerreiro et al., 2013a; Jonsson et al., 2013). In addition, gene network analyses of human AD brains and mouse models of AD have highlighted a central role for microglia in AD and, in particular, TREM2 and its binding partner TYRO protein tyrosine kinase-binding protein (TYROBP), also known as DNAX-activation protein 12 (DAP12) DAP12/TYROBP (Matarin et al., 2015; Zhang et al., 2013). Whereas heterozygous variants in *TREM2* are associated with AD (Guerreiro et al., 2013a; Jonsson et al., 2013), homozygous variants in *TREM2* or its binding partner *DAP12/TYROBP* cause polycystic lipomembranous osteodysplasia with sclerosing leukoencephalopathy (PLOSL), also known as Nasu-Hakola disease (NHD). NHD is a rare autosomal-recessive early-onset dementia characterized by behavioral changes and cognitive decline, with or without pathological bone fractures (Guerreiro et al., 2013b; Paloneva et al., 2002). How TREM2 contributes to neurodegeneration remains poorly understood. Furthermore, studies investigating the impact of TREM2 signaling on the inflammatory response have produced conflicting results, demonstrating either an anti-inflammatory or a pro-inflammatory role for TREM2 (Hamerman et al., 2006; Jay et al., 2015, 2017; Sieber et al., 2013; Turnbull et al., 2006). Recent studies have identified a role for TREM2 in microglial survival (Wang et al., 2015), as well in controlling energy metabolism (Ulland et al., 2017). Several studies have identified a role for TREM2 in phagocytosis (Hsieh et al., 2009; Kawabori et al., 2015; Kleinberger et al., 2014; Takahashi et al., 2005; Xiang et al., 2016), although others have observed no effect (e.g., Wang et al., 2015). One possible explanation for some of these discrepancies may be species differences between rodent and human immune cells (Smith and Dragunow, 2014) or differences in phagocytic materials. To investigate the effects of

This is an open access article under the CC BY license (http://creativecommons.org/licenses/by/4.0/).

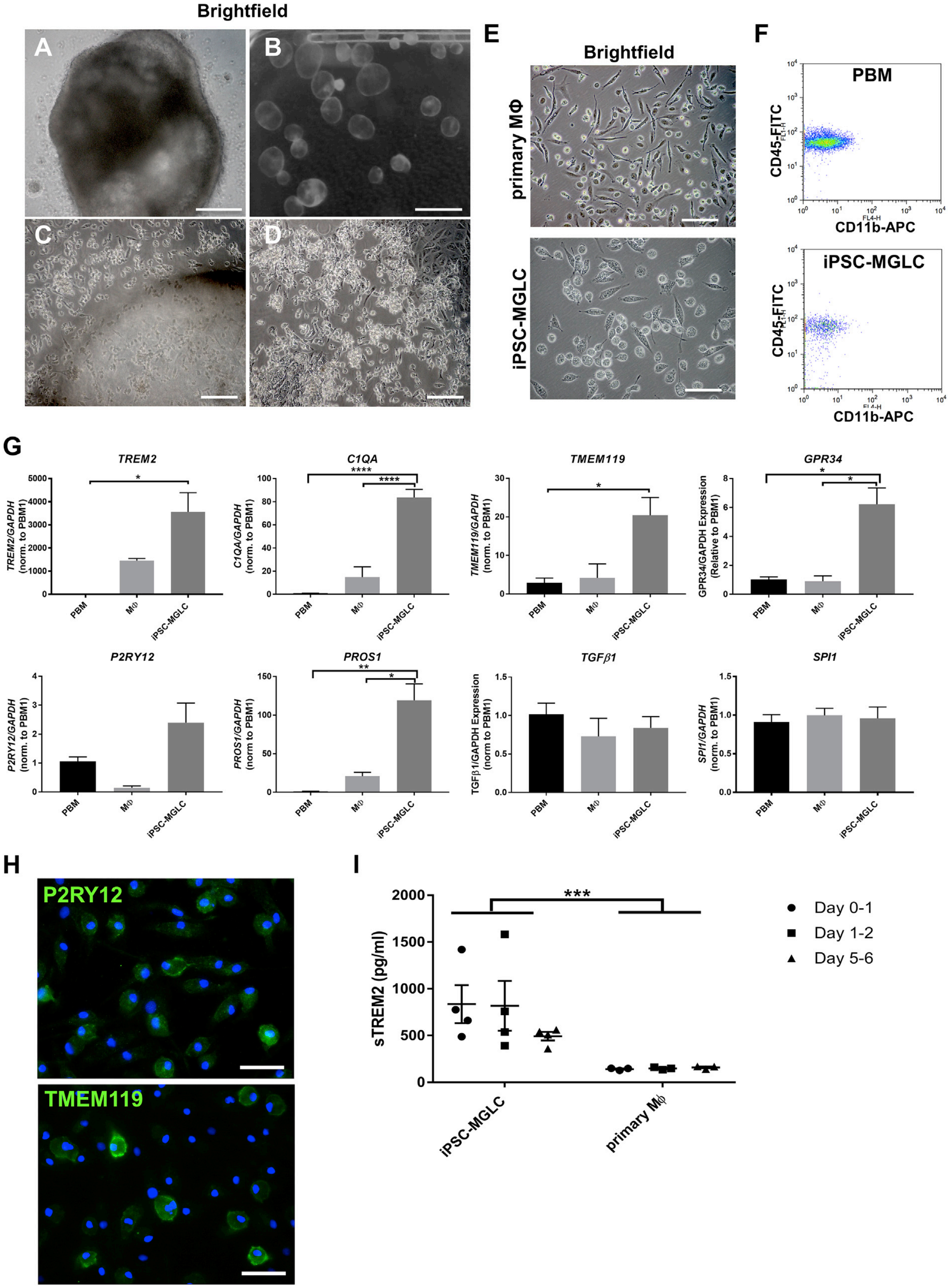

(legend on next page)

dementia-causing *TREM2* missense mutations on human macrophage function, we took advantage of a recently developed protocol to derive macrophages from human induced pluripotent stem cells (iPSCs) (van Wilgenburg et al., 2013). These iPSC-macrophages were shown to arise through a transcription factor MYB-independent developmental pathway, similar to yolk sac-derived tissue-resident macrophages such as brain-resident microglia (Buchrieser et al., 2017). We confirmed that the iPSC-macrophages we isolated are in fact very similar to microglia by demonstrating the expression of microglial genes, and we therefore refer to them as iPSC-microglial-like cells (iPSC-MGLCs). We tested whether iPSC-MGLCs could be used to study the role of TREM2 in neurodegeneration by generating iPSC-MGLCs from two patients with NHD caused by homozygous T66M and W50C TREM2 variants, as well as two unaffected relatives harboring one T66M variant allele and four controls expressing common variant TREM2. We confirmed that iPSC-MGLCs express and shed soluble TREM2 (sTREM2) protein and provide the first report to assess the functional consequences of the recently described W50C mutation in our iPSC-MGLC model. We identify deficits in the ability of cells harboring TREM2 missense mutations to survive a macrophage colony stimulating factor (MCSF) starvation regimen, and furthermore, to identify a specific deficit in phagocytosis. Taken together, these data provide insights into specific pathways known to be aberrant in chronic neurodegenerative pathologies and link these pathways to TREM2.

RESULTS

Generation of Human iPSC-MGLCs

We generated iPSC-MGLCs using recently developed macrophage differentiation protocols (van Wilgenburg et al., 2013), with minor modifications as detailed in the Supplemental Experimental Procedures. By generating embryoid bodies (EBs) in ultralow adherence 96-well plates (Figure 1A), we could reliably generate several million iPSC-MGLCs per week. Most EBs floated and generated large cystic structures (Figures 1B and 1C) or sometimes adhered to the bottom of the flasks (Figure 1D). Like other investigators (Hale et al., 2015; van Wilgenburg et al., 2013), we noticed the appearance of smaller-diameter cells 10–14 days after seeding EBs in myeloid progenitor medium containing MCSF and interleukin-3 (IL-3) that did not attach to tissue culture plates (not shown). Three to 4 weeks after seeding the EBs, the free-floating small cells were replaced by cells of a larger diameter, with fine processes that subsequently adhered to tissue culture plates and differed in morphology from primary macrophages (Mϕ) (Figure 1E), and they expressed similar levels of the myeloid markers CD45 and CD11b when compared to primary blood-derived monocytes (PBMs; Figure 1F). These cells could be harvested on a weekly basis, with several million iPSC-MGLCs being harvested from one 175-cm^2 flask containing approximately 150 EBs.

iPSC-MGLCs Express and Shed TREM2 and Have a Microglial Gene Signature

A recent study found that macrophages derived from iPSCs using this protocol are ontogenetically similar to tissue resident macrophages such as microglia (Buchrieser et al., 2017). We therefore investigated whether the iPSC-MGLCs generated here expressed the tissue-resident macrophage marker *TREM2* and other microglial genes. Expression of *TREM2*, as well as complement factor *C1QA*, *TMEM119*, *GPR34, PROS1*, genes that are preferentially expressed in microglia as opposed to monocytes (Butovsky et al., 2014), were higher in iPSC-macrophages than in PBM and/or primary Mϕ (Figure 1G: *$p < 0.05$, **$p < 0.01$, ****$p < 0.0001$). The levels of *SPI1* (encoding for the myeloid transcription factor PU1) and another microglial gene, *TGFb1*, were comparable between groups, whereas we observed a trend toward higher mRNA levels of P2RY12, another marker for microglia, in iPSC-macrophages compared with PBM and primary Mϕ (Figure 1G). Immunocytochemistry, with antibodies against the microglial genes P2RY12 and TMEM119, confirmed expression at the protein level of these microglial markers (Figure 1H). We also investigated whether iPSC-MGLCs shed sTREM2 into the extracellular space, as has been reported for cell lines and murine microglia (Kleinberger et al., 2014; Wunderlich et al., 2013). Levels of sTREM2 were measured in supernatants of iPSC-MGLCs and primary Mϕ at day 1 (D1), D2, and D6 in culture, and it was found that sTREM2 levels from iPSC-MGLCs were consistently higher than primary Mϕ in a period of 6 days (Figure 1I; iPSC-MGLC: 835 ± 203 pg/mL versus PBM 141 ± 6 pg/mL on D1, iPSC-MGLC 817 ± 266 pg/mL versus PBM 147 ± 7 pg/mL on D2, and iPSC-MGLC 492 ± 45 pg/mL versus PBM 159 ± 9.5 pg/mL on D6 of culture; ***$p < 0.001$).

In conclusion, our results demonstrate that iPSC-MGLCs differ substantially from PBMs and primary Mϕ through expression of a microglial gene signature, protein expression, and the functional shedding of TREM2. Taken together, these data allow us to refer to the cells as iPSC-MGLCs.

Figure 1. Generation and Characterization of iPSC-MGLCs

(A and B) Brightfield microscopy of an embryoid body (EB) after generation in a 96 well low adherence plate (A) and free floating EBs forming large cystic structures during further culture in myeloid progenitor differentiation medium (B).

(C and D) New iPSC-MGLCs are shed from either free-floating cystic EBs (C) or adherent cell layers (D).

(E) Comparison of iPSC-MGLCs with primary Mϕ cultured in M-CSF by brightfield microscopy.

(F) FACS analysis of the macrophage markers CD45 and CD11b in iPSC-MGLCs and primary blood-derived monocytes (PBMs), directly isolated from blood.

(G) qPCR analysis of *SPI1*, *TGFβ1*, or *P2RY12* levels, as well as the microglial markers *TREM2*, *C1QA*, *GPR34*, and *TMEM119* in iPSC-MGLCs, freshly isolated PBMs, and primary Mϕ (*$p < 0.05$, ****$p < 0.0001$ using one-way ANOVA with Dunnett's correction for multiple comparisons).

(H) Immunocytochemistry with antibodies against the microglial proteins P2RY12 and TMEM119 in iPSC-MGLCs.

(I) Secreted sTREM2 levels measured by ELISA from supernatant collected at days 1, 2, and 6 in iPSC-MGLCs and primary Mϕ. ***$p < 0.001$ for each cell type using two-way ANOVA.

Scale bars: 100 μm in (C)–(E), 250 μm in (A), 5 mm in (B), and 50 μm in (H). Data are represented as means ± SEMs; n = 3; **$p < 0.01$.

See also Figures S2, S3, and S4.

Table 1. Characteristics of TREM2 Variant and Control Common Variant iPSC Lines

iPSC Line	Age, y	Sex	Clinical Details	TREM2 Variant	ApoE Status
CTRL1	78	M	control	common variant	apoE3/E3
CTRL2	64	M	control	common variant	apoE2/E3
CTRL3	36	F	control	common variant	apoE2/E3
CTRL4	67	F	control	common variant	apoE3/E3
T66Mhet1	75	F	unaffected relative of T66Mhom	heterozygous T66M	apoE2/E3
T66Mhet2	47	M	unaffected relative of T66Mhom	heterozygous T66M	apoE3/E3
T66Mhom	51	M	NHD	homozygous T66M	apoE2/E3
W50Chom	36	F	NHD	homozygous W50C	apoE3/E3

NHD, Nasu-Hakola disease.

Generation of iPSC-MGLCs Harboring TREM2 Variants

To investigate the effects of TREM2 variants on microglial function, iPSC-MGLCs were generated from a patient with NHD caused by a homozygous TREM2 T66M variant, two unaffected relatives carrying a heterozygous TREM2 T66M variant, and one NHD patient carrying a homozygous TREM2 W50C variant (Table 1; Dardiotis et al., 2017; Guerreiro et al., 2013b). Both mutations reside in the extracellular domain of *TREM2* (Figure 2A). TREM2 mutant iPSCs were characterized in regard to karyotype and copy-number variation (CNV; Figure S1) pluripotency and genotype (Brownjohn et al., 2018). Four iPSC control lines were used for comparison (Table 1). Sanger sequencing of *TREM2* exon 2 confirmed the homozygous and heterozygous T66M variants and the homozygous W50C variant in the TREM2 mutant lines (Figure S2A). Two clones per line were used in all of the experiments.

The morphology of controls and TREM2 variant iPSC-MGLCs was similar (Figure S2B), as was the expression of the myeloid markers CD45 and CD11b (Figure S2C). The weekly iPSC-MGLC yield did not differ between control and TREM2 variant iPSC (Figure S2D). Immunocytochemistry confirmed that control and TREM2 variant iPSC-MGLCs displayed similar staining patterns of the macrophage markers Iba1 and CD68 (Figure S3A) and CD45 and the myeloid transcription factor PU.1 (Figure S3B). Overall, no consistent difference in the expression of microglial genes or AD risk factor genes was observed between controls and TREM2 variant iPSC-MGLCs (Figure S4A). Furthermore, using fluorescence-activated cell sorting (FACS) analysis, we did not detect a difference in CD33 or CSF1R surface expression between TREM2 variant iPSC-MGLCs (Figure S4B).

Reduced TREM2 Expression and Shedding in TREM2 Variant iPSC-MGLC

The subcellular localization of TREM2 in TREM2 variant iPSC-MGLCs was determined by confocal microscopy. In control iPSC-MGLCs, the staining pattern of TREM2 was diffuse in the cytosol, with additional prominent focal staining that colocalized with the Golgi complex (Figure 2B). TREM2 staining in T66Mhet iPSC-MGLC was similar to controls, with diffuse cytosolic staining as well as prominent perinuclear staining of TREM2 (Figure 2B). A significant reduction in TREM2 staining was observed in T66M homozygous (T66Mhomo) iPSC-MGLC and W50C iPSC-MGLCs, with an absence of focal staining (Figure 2B). *TREM2* mRNA levels did not differ between controls and T66Mhet iPSC-MGLCs, but they were markedly reduced in T66Mhom and W50Chom iPSC-MGLC (Figure 2C: $^{**}p < 0.01$). mRNA levels of the TREM2-binding protein *TYROBP*/*DAP12* did not differ between control and TREM2 variant iPSC-MGLCs (Figure 2D). Western blot analysis further demonstrated a reduction in TREM2 protein levels in both T66Mhet iPSC-MGLCs and T66Mhom/W50Chom iPSC-MGLCs (Figures 2E and S5A). The levels of immature TREM2 (Figures 2E and S5A, arrows) and mature, glycosylated TREM2 (Figures 2E and S5A, brackets) were reduced in TREM2 variant iPSC-MGLCs. Using antibody D8I4C, which also recognizes the C terminus of TREM2, we observed the presence of the C-terminal fragment (CTF) of TREM2 (resulting from protease-mediated cleavage of the protein at the cell membrane) (Wunderlich et al., 2013) and identified a significant reduction in T66Mhet, T66Mhom, and W50C iPSC-MGLCs compared with control iPSC-MGLCs, which is suggestive of altered processing of mutant TREM2 protein (Figures 2E and 2F). Quantification of TREM2 western blot (WB) bands with optical densitometry confirmed a significant reduction of immature, mature, and CTF TREM2 levels (Figure 2F: $^{*}p < 0.01$, $^{**}p < 0.001$, $^{****}p < 0.0001$).

Because of the reduced maturation of TREM2 observed here, we investigated whether TREM2 variants influenced the secretion of sTREM2 in our model. Cerebrospinal fluid sTREM2 levels were found to be significantly reduced in patients with dementia harboring NHD-associated TREM2 variants (Piccio et al., 2016), and reduced shedding of sTREM2 was observed in human kidney cell lines overexpressing NHD-associated mutant forms of TREM2 (Kleinberger et al., 2016). However, data on the secretion of sTREM2 in human iPSC-MGLCs expressing endogenous levels of mutant TREM2 protein are lacking. Using an in-house ELISA system, we measured sTREM2 levels in supernatants of control and TREM2 variant iPSC-MGLCs. sTREM2 levels were markedly reduced in T66Mhet iPSC-MGLC lines and were virtually absent in T66Mhom and W50Chom iPSC-MGLCs compared with controls (Figures 2G and S5B). Together, these results demonstrate that *TREM2* mRNA and protein expression, maturation, and shedding of sTREM2 are markedly affected by *TREM2* missense mutations in iPSC-MGLCs.

Growth Factor Withdrawal Triggers Cell Death in TREM2 Homozygous Missense iPSC-MGLCs

Previous studies have demonstrated an increased sensitivity of murine TREM2 knockout microglia to the withdrawal of

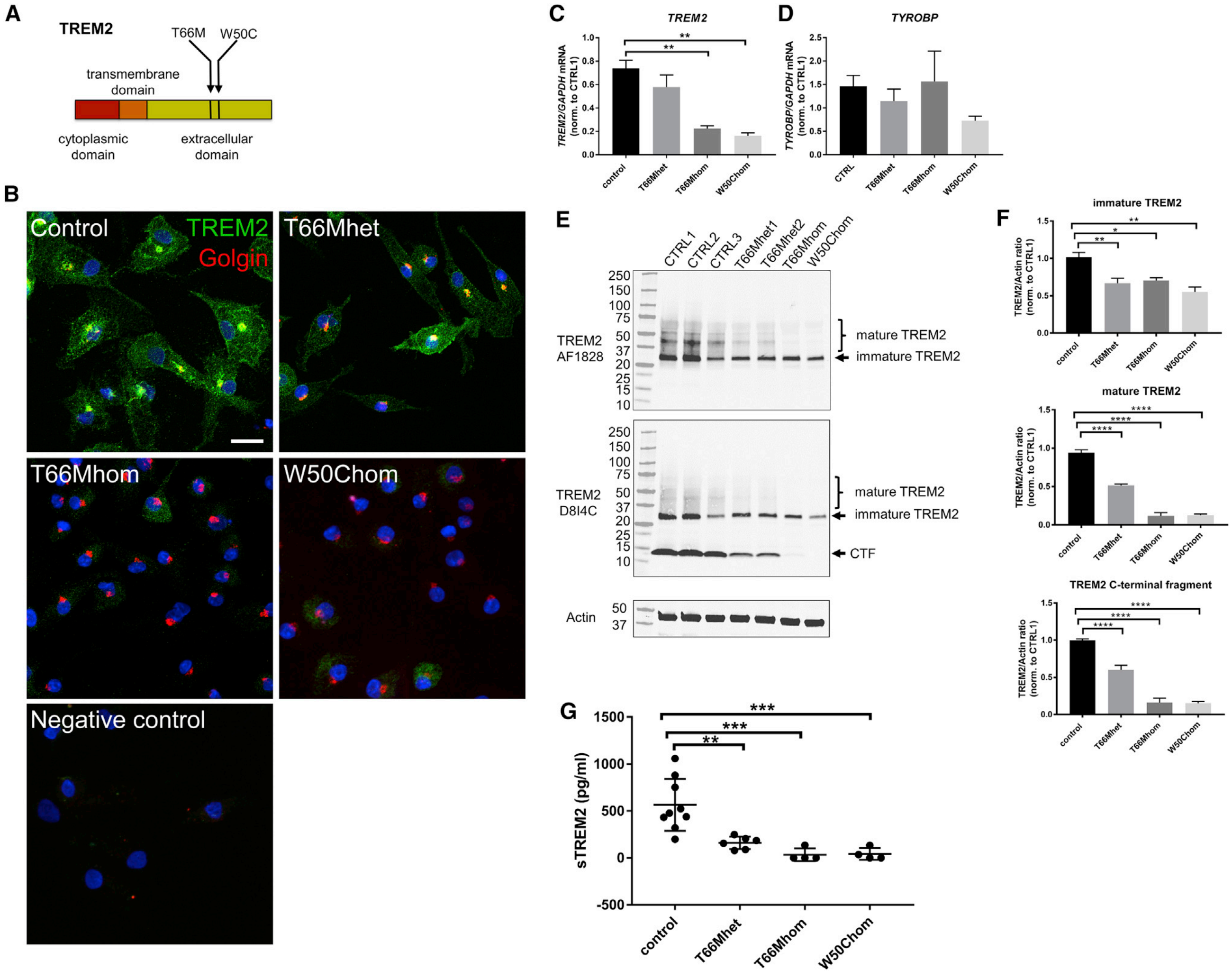

Figure 2. Characterization of TREM2 Expression and Secretion from Controls and TREM2 Variant iPSC-MGLCs

(A) Diagram of TREM2, indicating the T66M and W50C variants in the extracellular domain of TREM2.

(B) Immunocytochemistry and confocal imaging for TREM2 in control and TREM2 T66Mhet iPSC-MGLC and in TREM2 T66Mhom and W50Chom iPSC-MGLC. Scale bar: 20 μm.

(C and D) qPCR of *TREM2* (C) and *TYROBP/DAP12* (D) mRNA expression in control, T66Mhet, T66Mhom, and W50Chom iPSC-MGLCs (**$p < 0.01$).

(E) Western blot analysis of immature (arrows) and mature, glycosylated TREM2 protein (brackets) in *TREM2* T66Mhet, T66Mhom, and W50Chom compared with controls. D8I4C antibody detects a C-terminal fragment (CTF) of TREM2 (arrow labeled CTF).

(F) Quantification of TREM2 WB bands with optical densitometry; immature TREM2 levels reduced 34%, 30%, and 38%; mature TREM2 levels reduced 45%, 87%, and 86%; and TREM2 CTF levels reduced 39%, 83%, and 84% in T66Mhet, T66Mhom, and W50Chom iPSC-MGLCs compared with controls (n = 3, one clone per iPSC line, *$p < 0.01$, **$p < 0.001$, ****$p < 0.0001$).

(G) Quantification of sTREM2 secretion measured by ELISA after 24 hr in culture in T66Mhet, T66Mhom, and W50Chom iPSC-MGLCs compared with controls (control: 565 ± 92 pg/mL, T66Mhet: 160 ± 27 pg/mL, T66Mhom: 33 ± 33 pg/mL, W50Chom: 42 ± 31, **$p < 0.01$, ***$p < 0.001$). Data are means ± SEMs.

See also Figures S1, S2, S3, S4, and S5.

M-CSF, leading to cell death (Wang et al., 2015). iPSC-MGLCs were cultured for 24 and 72 hr in the presence or absence of 100 ng/mL M-CSF. Staining with the cell dye propidium iodide (PI), a marker of the loss of cell membrane integrity, was measured with FACS. No difference in PI uptake was observed either under basal conditions or following withdrawal of M-CSF for 24 hr between control and TREM2 variant iPSC-MGLCs (Figure 3A). However, after 72 hr withdrawal of M-CSF, T66Mhom cells showed a significant increase in PI uptake compared with controls, indicating an increased sensitivity to growth factor withdrawal, leading to cell death (Figure 3B).

Cytokine Secretion in Response to LPS in TREM2 Variant iPSC-MGLCs Does Not Differ from Controls

The effect of TREM2 variants on the secretion of inflammatory cytokines in response to pro-inflammatory stimuli was determined, as previous studies report opposing effects of TREM2 on inflammation (Sieber et al., 2013; Turnbull et al., 2006). Using

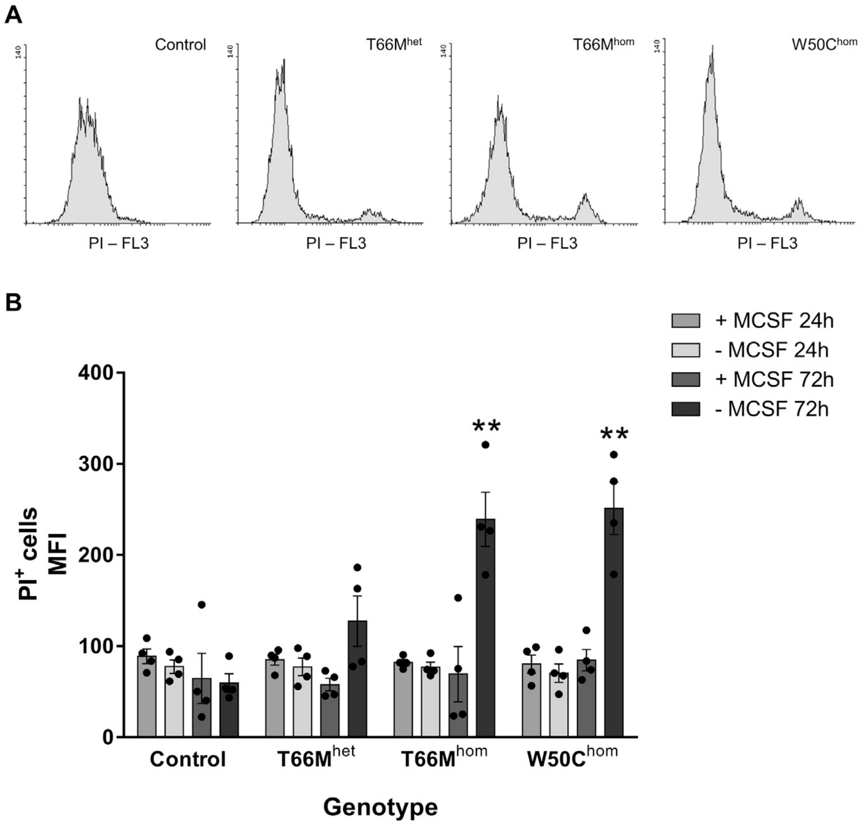

Figure 3. Survival in Control and TREM2 Variant iPSC-MGLCs

(A) FACS analysis of propidium iodide (PI) staining of iPSC-MGLCs following 24–72 hr incubation in DMEM ± 100 ng/mL M-CSF. Representative histograms from 72 hr DMEM minus M-CSF group (n = 4).

(B) Two to four control iPSC lines and 1–2 iPSC clones per TREM2 variant iPSC lines were analyzed, and mean fluorescence intensity was plotted.

Two-way ANOVA with Dunnett's multiple comparison was used for analysis. Data are presented as means ± SEMs; $^{**}p < 0.01$.

lipopolysaccharide (LPS), a well-characterized activator of Toll-like receptor-4 (TLR-4) and a binding partner of TREM2 (Daws et al., 2003), as a stimulant to activate iPSC-MGLCs, we undertook proteome analysis of 105 cytokines in two controls, 2 × T66Mhet lines, 1 × T66Mhom, and 1 × W50C iPSC clones (Figures 4A–4C, S6A, and S6B). An increase in the secretion of a number of cytokines, including tumor necrosis factor-α (TNF-α), IL-6, IL-8, and chitinase-3-like protein 1 (CHI3L1, also known as YKL-40), was observed following LPS stimulation, with small differences in release observed in T66Mhet and T66Mhom variant lines (Figures 4A–4C, S6A, and S6B). To investigate these differences more quantitatively, we used a V-PLEX Proinflammatory Panel 1 Human Kit (Meso Scale Diagnostics; Figures 4D and S6C). In this more sensitive assay, we found no significant differences in the levels of these cytokines between controls, T66Mhet, T66Mhom, and W50Chom iPSC-MGLCs, both under basal conditions and following LPS secretion (Figures 4D and S6C). Thus, *TREM2* missense mutations do not affect cytokine secretion under basal conditions or in the context of TLR-4 stimulation in human iPSC-MGLCs.

iPSC-MGLCs Harboring Missense *TREM2* Mutations Show Deficits in Phagocytosis

Because recent studies using TREM knockout (KO) mice and genetically manipulated cell lines have reported conflicting findings regarding the effect of TREM2 on phagocytosis (Kleinberger et al., 2014; Wang et al., 2015; Xiang et al., 2016), we investigated whether phagocytosis was impaired in human TREM2 variant iPSC-MGLCs. Non-evoked phagocytic activity was measured using fluorescent pH-sensitive rhodamine *Escherichia coli* (pHrodo *E. coli*), using FACS analysis. Cytochalasin D, a widely used inhibitor of phagocytosis, abolished the uptake of *E. coli* particles (Figures 5A and 5B). No significant differences were observed between T66Mhet, T66Mhom, and W50Chom iPSC-MGLCs when compared with control iPSC-MGLCs (Figure 5A). Furthermore, phagocytosis of pHrodo *E. coli* particles in iPSC-MGLCs (control cells) that had been treated with TREM2 or non-targeting small interfering (siRNA) showed no significant differences (Figure 5B), with the TREM2 knockdown being confirmed with qPCR, WB, and sTREM2 ELISA (Figures S7A–S7C). We also observed no difference in the phagocytosis of pHrodo zymosan, a putative ligand of the TLR-2 and TLR-6 receptors, when comparing genotypes (Underhill, 2003).

Because TREM2 binds phospholipids, including phosphatidylserine (Wang et al., 2015), which becomes exposed on the surface of apoptotic cells, we hypothesized that TREM2 may affect microglial phagocytosis in a substrate-specific way. We therefore measured the phagocytosis of apoptotic Vybrant CM-Dil dye-labeled human neuronal SH-SY5Y cells by FACS. Apoptosis was induced with UV radiation, and the presence of phosphatidylserine on the surface of apoptotic cells was confirmed with fluorescently labeled annexin V (Figure S7E). Cytochalasin D significantly inhibited the uptake of apoptotic SH-SY5Y cells (Figures 5C–5E). Comparing with controls, we found a marked reduction in the phagocytosis of apoptotic neuronal cells in T66Mhet, T66Mhom, and W50Chom (Figures 5C and 5D: $^{*}p < 0.5$, $^{***}p < 0.001$, $^{****}p < 0.0001$). We also found a significant reduction in apoptotic cell uptake in TREM2 siRNA versus non-targeting siRNA-treated iPSC-MGLCs, confirming a TREM2-specific effect (Figure 5E: $^{**}p < 0.01$). These data, taken together, suggest a substrate-specific deficit in phagocytic ability that is dependent on the TREM2 genotype or, more specifically, the availability of TREM2 protein for signaling.

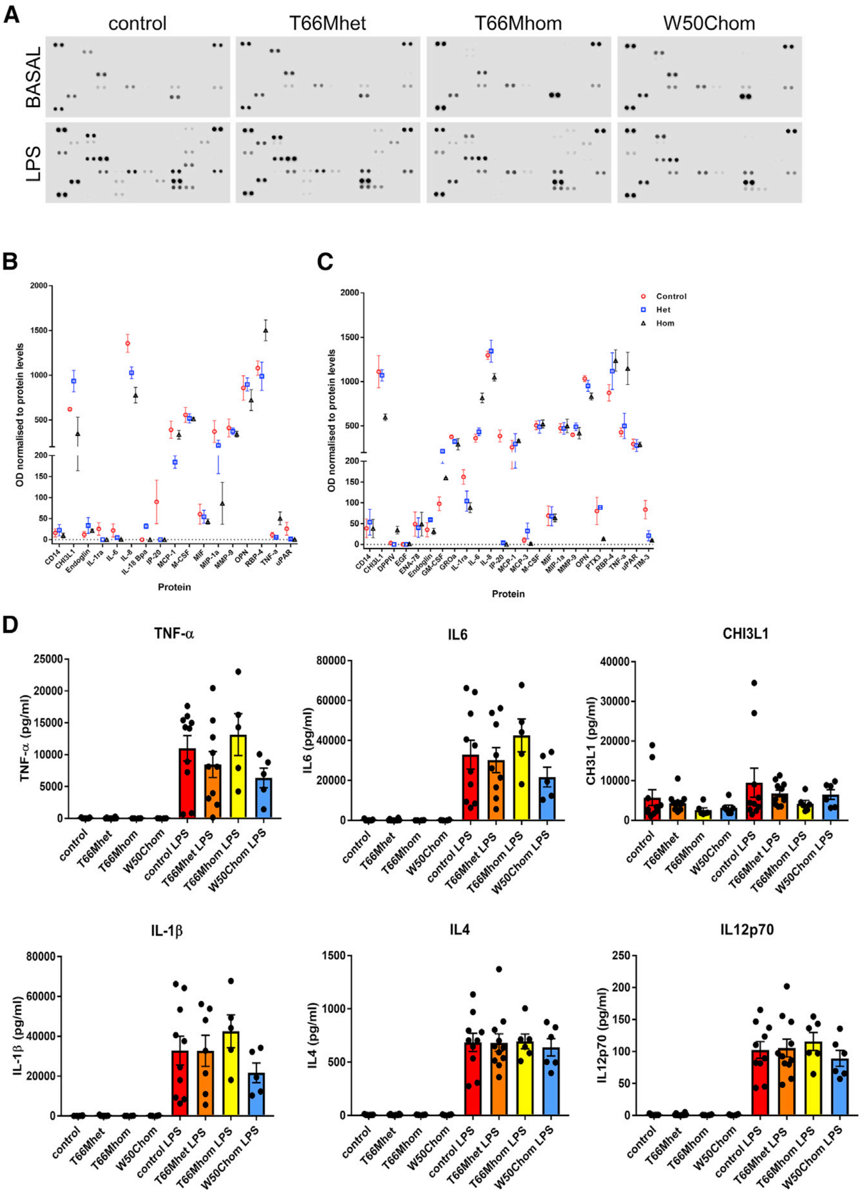

Figure 4. Analysis of Secreted Factors from Controls and TREM2 Variant iPSC-MGLCs after LPS Exposure

(A) Proteome profiles of secreted factors from control, T66Mhet, T66Mhom, and W50Chom iPSC-MGLCs analyzed using a Human XL Cytokine Array Kit (R&D Systems). Representative dot blots from iPSC-MGLC culture supernatants from one control line, one T66Mhet line, one T66Mhom, and W50Chom iPSC-MGLCs are shown under basal (untreated) conditions and LPS-treated conditions; 100 ng/mL after 18 hr.

(B and C) Analysis of dot blot intensity from supernatants of basal (B) and LPS-treated (C) cultures. Mixed supernatants from independent experiments were used; n = 3 for all genotypes.

(D) Levels of 10 cytokines, including TNF-α, IL-6, CHI3L1, IL-1β, IL-4, and IL-12p70 were measured simultaneously on the Meso Scale Diagnostics (MSD) platform using a V-PLEX Pro-inflammatory Panel 1 (n = 2 for untreated, n = 3 for LPS-treated samples in four control iPSC lines, and two clones per TREM2 variant iPSC line were analyzed; data are presented as mean ±SEM).

See also Figure S6.

iPSC-MGLCs Harboring Missense *TREM2* Mutations Show Deficits in Chemokine Release

Deficits in the ability of cells harboring TREM2 missense mutations to phagocytose apoptotic cells suggest either aberrant ligand recognition, defects in the cells' ability to efficiently migrate to areas of damage, or both. Using a proteome profiler array, we identified clear deficits in the release of particular cytokines related to chemotaxis and chemoattraction, namely MIP1α and CXCL10, from cells harboring the T66Mhom mutation when exposed to apoptotic cells (Figures 6A, 6B, S7F, and S7G). Furthermore, scratch assays to determine the ability of cells to migrate toward an area of damage, in this case a concentration of apoptotic cells, identified a significant deficit in iPSC-MGLCs from T66Mhom patient lines (Figures 6C and 6D). Taken together, these data strongly suggest a global deficit in the ability of cells harboring TREM2 missense mutations to efficiently respond to signaling associated with neurodegenerative pathologies.

DISCUSSION

Transcriptome analysis indicated that the macrophages or microglial-like cells derived from iPSCs have a high expression of genes associated with tissue-resident macrophages (Alasoo et al., 2015). A recent study using clustered regularly interspaced short palindromic repeats (CRISPR)/CRISPR-associated protein-9 nuclease (Cas9) demonstrated that iPSC-macrophages develop in an MYB-independent manner that is characteristic of tissue-resident macrophages, including microglia (Buchrieser et al., 2017). Furthermore, another recent study demonstrated that iPSC-microglia generated with the same protocol used in the present study have a gene transcription profile that is very similar to primary human microglia, which can be brought even closer by co-culturing with neurons (Haenseler et al., 2017). In addition to the tissue-resident macrophage marker *TREM2*, we found that our iPSC-MGLCs expressed *C1QA*, *TMEM119*, *GPR34*, and *PROS1*, which were previously identified as microglial markers (Butovsky et al., 2014). Whereas the mRNA levels of the microglial gene *P2RY12* were not markedly different from blood-derived monocytes, we found that iPSC-MGLCs nevertheless showed positive staining for P2RY12 by immunocytochemistry. Differences in the transcriptome between microglia, the resident macrophages of the brain, and macrophages were

OPEN
ACCESS
CellPress

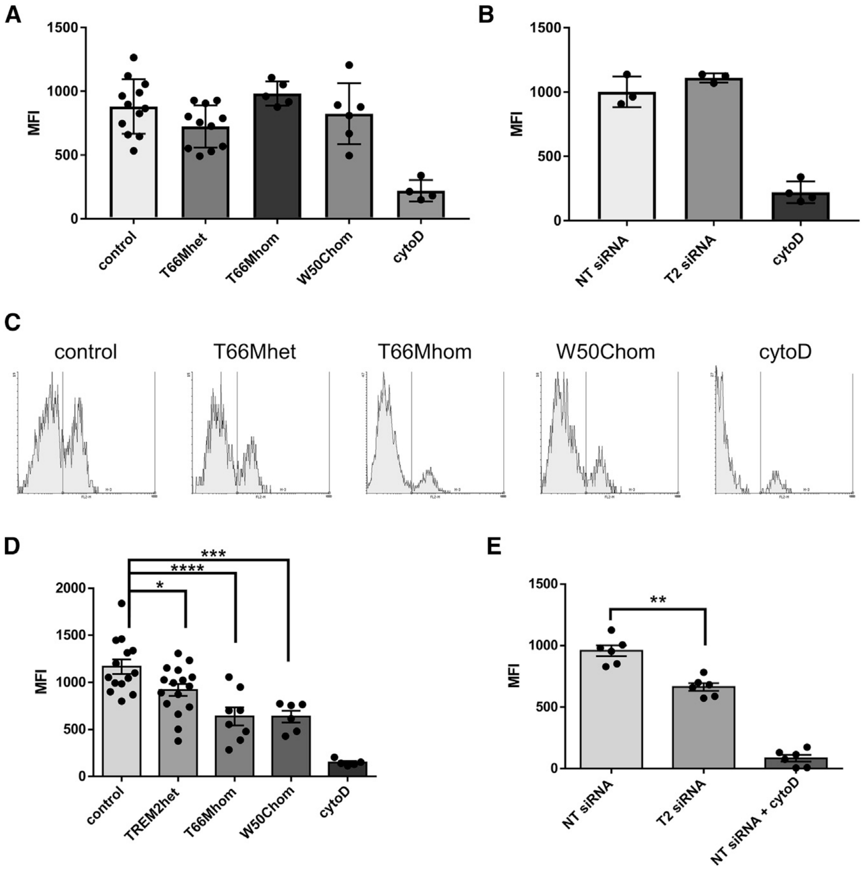

Figure 5. Analysis of Pathogen- and Apoptotic Cell-Associated Phagocytosis

(A) FACS analysis to measure uptake of pHrodo *E. coli* particles in variant TREM2 iPSC-MGLCs compared with controls. n = 3, four control iPSC lines and two iPSC clones per TREM2 variant iPSC line were analyzed.

(B) FACS analysis to measure uptake of pHrodo *E. coli* particles following siRNA knockdown of TREM2 in iPSC-MGLCs (T2 siRNA) or non-targeting siRNA (non-targeting [NT] siRNA) (n = 3).

(C) FACS analysis of phagocytosis of fluorescent dye-labeled apoptotic SH-SY5Y cells by control and TREM2 variant iPSC-MGLCs. Quantification of phagocytosis shown.

(D) 21%, 45%, and 45% reduction in T66Mhet, T66Mhom, W50Chom, respectively, compared with controls (n = 4, $^*p < 0.5$, $^{***}p < 0.001$, $^{****}p < 0.0001$; four control iPSC lines and two iPSC clones per TREM2 variant iPSC lines were analyzed).

(E) FACS analysis of phagocytosis of fluorescent dye-labeled apoptotic SH-SY5Y cells in iPSC-MGLC treated with TREM2 siRNA (T2 siRNA) or non-targeting siRNA (NT siRNA).

Presented siRNA data are from experiments using one control iPSC line for pHrodo *E. coli* or pooled normalized data from two control iPSC lines (for phagocytosis of apoptotic neuronal cells). Student's t test was used for the analysis of siRNA experiments and one-way ANOVA with Dunnett's correction for comparison between control and TREM2 variant iPSC-MGLC; data are presented as mean ± SEM.

See also Figure S7.

recently reported (Butovsky et al., 2014; Hickman et al., 2013); it is worth noting, however, that microglia and other macrophages share many similarities, including the ability to phagocytose and secrete pro-inflammatory cytokines. Recently, other protocols to generate human iPSC-MGLCs have been described (Muffat et al., 2016; Pandya et al., 2017). We used a protocol that allows the robust generation of large numbers of iPSC-MGLCs from several iPSC lines. We have shown that these iPSC-MGLCs serve as a strong model to study the impact of *TREM2* variants on phagocyte function and have demonstrated a strong deficit in the ability of iPSC-MGLCs harboring TREM2 missense mutations to phagocytose endogenously associated debris, with deficits observed in the ability to efficiently migrate to areas mimicking pathology.

Initial characterization identified mRNA, and protein expression, maturation, and shedding of sTREM2 into the extracellular space are markedly reduced in T66Mhet, T66Mhom, and W50Chom, with virtually undetectable sTREM2 in TREM2 homozygous iPSC-MGLCs. Given the recently developed role of sTREM2 in promoting macrophage survival and inflammatory responses (Wu et al., 2015; Zhong et al., 2017), the reduction or absence of sTREM2 is likely to contribute to macrophage dysfunction in NHD. Reduced cleavage of TREM2 from the cell surface has also been shown to affect TREM2 intracellular signaling (Glebov et al., 2016; Wunderlich et al., 2013). Further studies are needed to determine whether sTREM2 influences other aspects of microglial function and interactions of microglia with other brain cells. *TREM2* mRNA levels were previously found to be reduced in patients with NHD caused by TREM2 variants (Chouery et al., 2008; Sasaki et al., 2015), and we found this to be the case for T66Mhom and W50Chom but not for T66Mhet iPSC-MGLCs. We found no difference in the mRNA and protein surface expression level of the dementia-causing or associated genes *CSF1R* and *CD33*. It was recently reported that *CD33* modulates TREM2 surface expression in human peripheral blood mononuclear cells (Chan et al., 2015) and that TREM2 KO macrophages have increased apoptosis in response to the withdrawal of CSF-1 (Wu et al., 2015). At least under basal conditions, we found no impact of NHD-causing TREM2 variants on these 2 AD-associated macrophage receptors in iPSC-MGLCs. However, after analyzing the sensitivity of TREM2 variant iPSC-MGLCs to growth factor withdrawal, we observed an increase in cell death in TREM2 homozygous missense mutation-harboring iPSC-MGLCs, when starved for 72 hr (three times the length of time used for TREM2 KO mouse microglia; Wang et al., 2015).

No deficits were observed in non-endogenous phagocytic ligands of *E. coli* or zymozan-mediated cytokine release, suggesting that TREM2 missense mutations alone are not sufficient to alter these phagocytic mechanisms. This was further supported by siRNA studies that showed no difference in *E. coli* phagocytosis. However, phagocytosis of apoptotic neuronal cells was markedly reduced in both *TREM2* heterozygous and

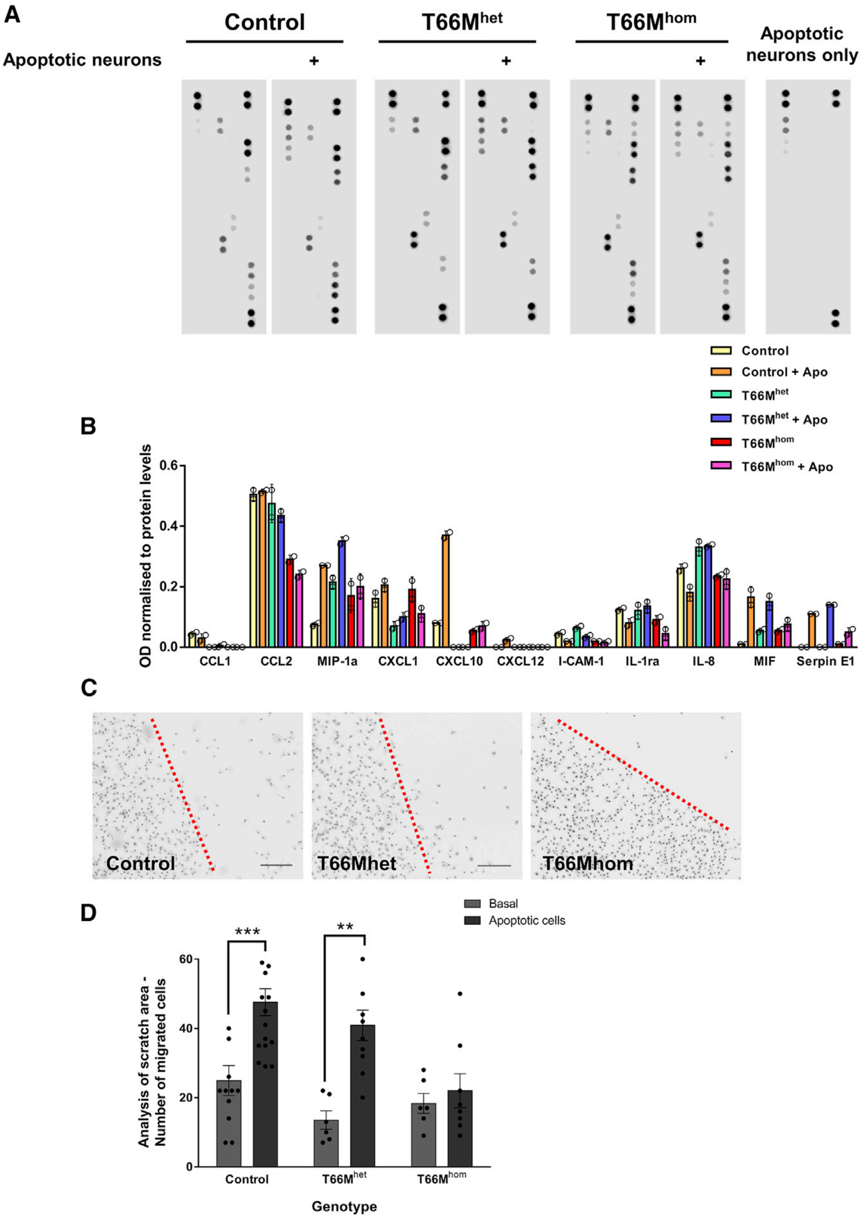

Figure 6. Apoptotic Cell-Mediated Cytokine Responses Are Dependent on Genotype

(A) Proteome profiles of secreted factors from control, T66Mhet, and T66Mhom iPSC-MGLCs were analyzed using a human cytokine array. Representative dot blots from pooled cell culture supernatants from two control, T66Mhet, and T66Mhom lines, and apoptotic cells only are shown under basal (untreated) conditions and following exposure to apoptotic SH-SY5Y cells for 48 hr.

(B) Analysis of dot blot intensity from supernatants of basal cell- and apoptotic cell-exposed cultures revealed modulation of secreted factors associated with chemotaxis and chemoattraction with a dependence on genotype

(C and D) Mixed supernatants from independent experiments were used; n = 3 for all genotypes. Representative images from cell culture scratch assays. Scale bar: 200 μm (C). Cultures were imaged live after 24h exposure to trans-wells containing apoptotic SH-SY5Y and subsequent Hoechst staining (D). Analysis was performed using ImageJ thresholding and particle quantification of the scratch area in (D). Two-way ANOVA with Bonferroni multiple comparison was used for analysis. Data are presented as means ± SEMs from two control lines, and one T66Mhet, and one T66Mhom line; n = 3 (**$p < 0.01$, ***$p < 0.001$).

See also Figure S7.

homozygous iPSC-MGLCs in a gene dosage-dependent manner and confirmed with siRNA knockdown studies. The fact that TREM2 heterozygous variants in particular impair the phagocytosis of apoptotic cells is of relevance to AD, in which heterozygous TREM2 variants (mainly the R47H variant) were found to be associated with AD (Guerreiro et al., 2013a; Jonsson et al., 2013), although further studies with cells from patients harboring this specific mutation are required for confirmation. A possible explanation for the observed substrate-dependent impairment in phagocytosis may be that *E. coli* or zymosan particles, known ligands of TLR4 and TLR2, respectively, do not engage the TREM2 receptor, whereas apoptotic cells (possibly through phosphatidylserine and other phospholipid molecules on their surface) do, and could therefore induce TREM2 signaling, which may boost phagocytic capability. Our results using human iPSC-MGLCs extend previous studies looking at the effects of TREM2 on phagocytosis, including those in TREM2-overexpressing cell lines, isolated microglia from TREM2 KO mice, and CRISPR-Cas9-manipulated microglial cell lines (Atagi et al., 2015; Kleinberger et al., 2014; Takahashi et al., 2005; Xiang et al., 2016). We saw a reduction in the phagocytosis of apoptotic cells in all TREM2 disease variants (including two clones per TREM2 variant iPSC line), with a gene dose response in TREM2 variants, strongly suggesting that these data did not result from other human genetic variability.

The strength of our approach lies in the fact that we have studied the biology of TREM2 in patient-derived iPSC-MGLCs that harbor NHD-causing TREM2 variants and express TREM2 at endogenous levels. We conclude that these iPSC-MGLCs serve as a strong model to study TREM2 and other myeloid genes associated with neurodegenerative disease, including NHD and AD. Both TREM2 heterozygous and homozygous variants affected the phagocytosis of apoptotic cells, with observations from cytokine array profiles suggesting deficits in chemotaxis and chemoattractant pathways when harboring a TREM2 homozygous mutation, which were supported by deficits in functional migration studies and thus supported previously published data

(Mazaheri et al., 2017). Further work investigating the mechanisms of how TREM2 influences the reaction of microglia to neuronal injury may shed light on the microglial dysfunction in neurodegenerative diseases and identify suitable drug targets for future treatments.

EXPERIMENTAL PROCEDURES

Generation of Human iPSC-Derived Microglia-Like Cells

TREM2 variant primary fibroblast lines were generated from 4 mm skin punch biopsies, obtained under informed consent. Ethical permission for this study was obtained from the National Hospital for Neurology and Neurosurgery and the Institute of Neurology joint research ethics committee (study reference 09/H0716/64) or approved by the ethics committee of the Istanbul Faculty of Medicine, Istanbul University (for collection of T66M mutant fibroblasts to Dr. Ebba Lohmann). iPSCs were generated from fibroblast cultures using 4-factor Sendai virus reprogramming at The Gurdon Institute and the Department of Biochemistry, University of Cambridge (Cambridge, UK). TREM2 mutations were confirmed with Sanger sequencing (Figure 3A), and pluripotency was confirmed (Brownjohn et al., 2018). Human iPSC-derived MGLCs were generated using recently published protocols (van Wilgenburg et al., 2013), with minor modifications (see Supplemental Experimental Procedures).

Isolation of Human Blood-Derived Monocytes

Monocytes were obtained from blood through centrifugation with Histopaque (Sigma, USA) to isolate peripheral blood mononuclear cells followed by separation with CD14-labeled magnetic beads (Miltenyi, USA). Primary blood-derived monocytes (PBMs) were either analyzed immediately or matured into primary macrophages (Mϕ) by incubation with macrophage end-differentiation medium: X-Vivo 15 medium (Lonza, Switzerland) with 1% GlutaMAX (Life Technologies, USA), 100 U penicillin/streptomycin (Life Technologies), and 100 ng/mL M-CSF (PeproTech, USA).

sTREM2 ELISA

iPSC-MGLCs were seeded at 4 × 10^4 cells/well in 96-well plates (Corning, USA) in macrophage end-differentiation medium. The following day, the medium was changed to fresh medium, and supernatants were collected after 24 hr. Quantification of sTREM2 from cell culture supernatants was performed using an in-house-generated ELISA using MaxiSORP 96-well plates (Nunc, Thermo Fisher Scientific, USA) coated with 1 μg/mL of a rat anti-mouse/human TREM2 monoclonal antibody (clone 237920, R&D Systems, USA) overnight at 4°C. After a blocking step, cell culture supernatant samples and standards (recombinant human TREM2-His; Life Technologies) were incubated for 2 hr at room temperature (RT) with biotinylated polyclonal goat anti-human TREM2 capture antibody (0.1 μg/mL; AF1828, R&D Systems). After incubation with streptavidin-horseradish peroxidase (HRP) (0.1 μg/mL; Invitrogen), followed by the addition of a chromogenic substrate solution (TMB, Life Technologies), the reaction was terminated with the addition of stop solution (0.16 M H_2SO_4) and absorbance was read at 450 nm (GENios, Tecan, USA). In addition, a second method using a mesoscale assay validated the results (see Supplemental Experimental Procedures).

FACS

At least 1 × 10^5–1 × 10^6 cells per iPSC line were used for FACS analysis. Cells were harvested into PBS without Ca^{2+}/Mg^{2+}. Cells were washed with FACS buffer (PBS + 0.5% BSA (Sigma) + 2 mM EDTA), incubated with Fc block (1:20, Miltenyi) and primary antibodies (1:10) or isotype control antibodies (1:10) for 1 hr at 4°C, washed three times with FACS buffer, and analyzed using a Becton Dickinson FACSCalibur analyzer. The results were analyzed using FlowJo software (version 8.8.7; Tree Star, USA).

PI Cell Death Assay

iPSC-MGLCs were seeded 3 days before the assay in 6-well plates (Corning) at 5 × 10^5 cells/well in macrophage end-differentiation medium. The day before the assay, the medium was changed to DMEM (Life Technologies), with or without 100 ng/mL M-CSF. After 24–72 hr, supernatants were collected, cells were rinsed with PBS without Ca^{2+}/Mg^{2+}, and cells were harvested with trypsin LE medium (Lonza). Cells were pelleted and resuspended in FACS buffer before incubation for 10 min with PI (Miltenyi). Cells were analyzed using a Becton Dickinson FACSCalibur analyzer, and data were analyzed using Flowing software version 2.5.1.

Cytokine Arrays and Pro-inflammatory Cytokine Panel

iPSC-MGLCs were seeded at 2 × 10^4 cells/well in 96-well plates (Corning) in macrophage end-differentiation medium. The following day, cells were stimulated with 100 ng/mL LPS (Sigma) or 1 × 10^5 apoptotic SH-SY5Y cells. Proteome Profiler Human XL Cytokine antibody array membranes or human cytokine array membranes (R&D Systems) were incubated with supernatants pooled from three independent experiments, according to *TREM2* genotype after basal, LPS, or apoptotic cell treatment, as per the manufacturer's instructions. Data were analyzed using the Protein Array Analyzer Palette plug-in (ImageJ, USA), and plotted as a mean ± SEM after normalizing to membrane reference positive controls and intracellular protein concentrations. The levels of 10 cytokines were measured using a V-PLEX Proinflammatory Panel 1 Human Kit (Meso Scale Diagnostics, USA) on a SECTOR Imager 6000 analyzer (Meso Scale Diagnostics), according to the manufacturer's instructions.

Transfection with TREM2 siRNA

iPSC-MGLCs were seeded at 5 × 10^5 cells/well in 6-well plates (Corning) for protein or siRNA extraction, or 1 × 10^5 cells/well in 24-well plates (Corning) for phagocytosis assay or sTREM2 ELISA. Cells were transfected using SMARTpool ON-TARGETplus TREM2 (Dharmacon, USA) or non-targeting siRNA (Horizon Discovery, UK) and Lipofectamine RNAiMAX reagent (Thermo Fisher Scientific), according to the manufacturer's instructions. Medium was changed before transfection. Cells were transfected 72 hr before assays or collection.

Phagocytosis of pHrodo *E. coli or* pHrodo Zymosan

iPSC-MGLCs were seeded at 1 × 10^5 cells/well in 24-well plates (Corning) 48 hr before the assay. As a negative control, cells were preincubated for 30 min with 10 μM cytochalasin D (Sigma). Cells were incubated with pHrodo *E. coli* (50 μg) or pHrodo zymosan (25 μg) (Life Technologies) particles for 2 hr. Cells were analyzed with a Becton Dickinson FACSCalibur flow cytometer, and results were analyzed with Flowing software (Cell Imaging Core of the Turku Centre for Biotechnology, flowingsoftware.btk.fi).

Phagocytosis of Apoptotic Neuronal Cells

SH-SY5Y cells (a gift from Dr. R. de Silva, University College London Institute of Neurology [UCL ION]) were seeded at 4 × 10^5 cells/well in 6-well plates in DMEM with 10% heat-inactivated bovine calf serum (Life Technologies) and 1% penicillin/streptomycin (Life Technologies). The following day, cells were loaded with Vybrant CM-DiI dye (1:200; Thermo Fisher Scientific) for 15 min, and the medium was changed to 500 μL PBS. Cells were irradiated with 500 J/m^2 using a UV Crosslinker (Stratech, UK) and then incubated for 24 hr in normal DMEM. Apoptosis was confirmed using fluorescein isothiocyanate (FITC)-labeled annexin V (Miltenyi; see Figure S4B). Dye-labeled apoptotic SH-SY5Y cells were harvested in PBS without Ca^{2+}/Mg^{2+}, spun down, resuspended in macrophage end-differentiation medium, and counted. A total of 500,000 apoptotic SH-SY5Y cells were added to each well of the iPSC-MGLCs (seeded at 1 × 10^5 cells/well in 24-well plates for 7–9 days and changed into 200 μL of fresh macrophage end-differentiation medium before the assay) for 2 hr. As a negative control, iPSC-MGLCs were pre-incubated for 30 min with 10 μM cytochalasin D (Sigma). iPSC-MGLCs were harvested with trypsin LE (Life Technologies) before being re-suspended in FACS buffer and analyzed using a Becton Dickinson FACSCalibur analyzer. Data were analyzed using Flowing software version 2.5.1.

Functional Migration Studies Using Scratch Assays and Trans-Wells

iPSC-MGLCs were scratched away in a line down the central area of the population with a sterile metal spatula, followed by washing to remove residual floating cells. Heat-shocked apoptotic SH-SY5Y cells were added to trans-wells (0.4 μm pore size) and placed over the scratched region for 24 hr. Trans-wells were removed before live cell staining with Hoechst 33342 for 45 min, followed by image capture on a Zeiss Axioskop 2 fluorescent microscope and image analysis using AxioVision 4.8 and ImageJ software.

Statistical Analysis

Results were analyzed using Prism software (version 7, GraphPad, USA). One-way ANOVA with Dunnett's correction for multiple comparisons was used unless otherwise indicated. Statistical analysis was performed on pooled controls and separate groups for each TREM2 heterozygous or homozygous mutation.

SUPPLEMENTAL INFORMATION

Supplemental Information includes Supplemental Experimental Procedures and seven figures and can be found with this article online at https://doi.org/10.1016/j.celrep.2018.07.094.

ACKNOWLEDGMENTS

P.G.R. was supported by a clinical research fellowship from Alzheimer's Research UK. A.P. was supported by a PhD studentship from Complement UK/Alexion (to J.M.P., J.H., and S. Lovestone). T.M.P. and M.B. were scientists at UCL, supported by Eisai, working with the Eisai:UCL Therapeutic Innovation Group (TIG). T.M.P. was also supported by funding to J.M.P. and J.H. from the Innovative Medicines Initiative 2 Joint Undertaking under grant agreement 115976. The Joint Undertaking receives support from the European Union's Horizon 2020 research and innovation programme and the European Federation of Pharmaceutical Industries and Associations (EFPIA). C.V.-L. was supported by a studentship from the Peruvian government. C.R. was supported by the Alzheimer's Society, UK. C.E.A. was supported by the National Institute for Health Research University College London Hospitals Biomedical Research Centre. A.H. was supported by funding from UK Dementia Research Institute, University College London and the Leonard Wolfson Experimental Neurology Centre, University College London. H.Z. is a Wallenberg Academy Fellow.

This work was supported by the UK Medical Research Council funding to the MRC Dementia Platform UK (MR/M02492X/1) and Medical Research Council core funding to the High-Content Biology Platform at the Medical Research Council-University College London Laboratory for Molecular Cell Biology university unit (MC_U12266B).

We thank K. Smith for access to his confocal microscope, D. Schiza and R. Desai for their assistance with the confocal microscope, and K. Cosker for invaluable advice on key experiments. We would like to thank the patients and their families for their participation in this research project; P. Brownjohn and F. Livesey, The Gurdon Institute, ARUK Stem Cell Research Centre and Department of Biochemistry, University of Cambridge for providing cell lines and advice on protocols; E. Lohmann, Istanbul University Faculty of Medicine, Department of Neurology, Behavioral Neurology and Movement Disorders Unit; S. Cowley, William Dunn School of Pathology, University of Oxford; S. Mukhopadhyay, Sanger Institute; and M. Matthews, Institute of Reconstructive Neurobiology, University of Bonn for their help with cells and advice with the iPSC macrophage protocols.

AUTHOR CONTRIBUTIONS

Conceptualization, P.G.R., J.H., and J.M.P. Methodology, P.G.R., A.P., M.B., T.M.P., C.V.-L., C.E.A., C.R., A.H., H.Z., and J.M.P. Formal Analysis, P.G.R. A.P., T.M.P., A.M., C.V.-L., and J.M.P. Investigation, P.G.R., A.P., T.M.P., A.M., C.V.-L., A.H., and J.M.P. Writing – Original Draft, P.G.R. and J.M.P. Writing – Review & Editing, J.M.P., P.G.R., T.M.P., and A.P. Visualization, P.G.R, T.M.P., A.P., and J.M.P. Resources, H.N., H.Z., H.H., and S.N. Supervision, J.M.P. and J.H. Funding Acquisition: P.G.R., J.H., and J.M.P.

DECLARATION OF INTERESTS

The authors declare no competing interests.

Received: May 3, 2018
Revised: May 31, 2018
Accepted: July 27, 2018
Published: August 28, 2018

REFERENCES

Alasoo, K., Martinez, F.O., Hale, C., Gordon, S., Powrie, F., Dougan, G., Mukhopadhyay, S., and Gaffney, D.J. (2015). Transcriptional profiling of macrophages derived from monocytes and iPS cells identifies a conserved response to LPS and novel alternative transcription. Sci. Rep. *5*, 12524.

Atagi, Y., Liu, C.-C., Painter, M.M., Chen, X.-F., Verbeeck, C., Zheng, H., Li, X., Rademakers, R., Kang, S.S., Xu, H., et al. (2015). Apolipoprotein E Is a Ligand for Triggering Receptor Expressed on Myeloid Cells 2 (TREM2). J. Biol. Chem. *290*, 26043–26050.

Brownjohn, P.W., Smith, J., Solanki, R., Lohmann, E., Houlden, H., Hardy, J., Dietmann, S., and Livesey, F.J. (2018). Functional Studies of Missense TREM2 Mutations in Human Stem Cell-Derived Microglia. Stem Cell Reports *10*, 1294–1307.

Buchrieser, J., James, W., and Moore, M.D. (2017). Human Induced Pluripotent Stem Cell-Derived Macrophages Share Ontogeny with MYB-Independent Tissue-Resident Macrophages. Stem Cell Reports *8*, 334–345.

Butovsky, O., Jedrychowski, M.P., Moore, C.S., Cialic, R., Lanser, A.J., Gabriely, G., Koeglsperger, T., Dake, B., Wu, P.M., Doykan, C.E., et al. (2014). Identification of a unique TGF-β-dependent molecular and functional signature in microglia. Nat. Neurosci. *17*, 131–143.

Chan, G., White, C.C., Winn, P.A., Cimpean, M., Replogle, J.M., Glick, L.R., Cuerdon, N.E., Ryan, K.J., Johnson, K.A., Schneider, J.A., et al. (2015). CD33 modulates TREM2: convergence of Alzheimer loci. Nat. Neurosci. *18*, 1556–1558.

Chouery, E., Delague, V., Bergougnoux, A., Koussa, S., Serre, J.-L., and Mégarbané, A. (2008). Mutations in TREM2 lead to pure early-onset dementia without bone cysts. Hum. Mutat. *29*, E194–E204.

Dardiotis, E., Siokas, V., Pantazi, E., Dardioti, M., Rikos, D., Xiromerisiou, G., Markou, A., Papadimitriou, D., Speletas, M., and Hadjigeorgiou, G.M. (2017). A novel mutation in TREM2 gene causing Nasu-Hakola disease and review of the literature. Neurobiol. Aging *53*, 194.e13–194.e22.

Daws, M.R., Sullam, P.M., Niemi, E.C., Chen, T.T., Tchao, N.K., and Seaman, W.E. (2003). Pattern recognition by TREM-2: binding of anionic ligands. J. Immunol. *171*, 594–599.

Glebov, K., Wunderlich, P., Karaca, I., and Walter, J. (2016). Functional involvement of γ-secretase in signaling of the triggering receptor expressed on myeloid cells-2 (TREM2). J. Neuroinflammation *13*, 17.

Guerreiro, R., Wojtas, A., Bras, J., Carrasquillo, M., Rogaeva, E., Majounie, E., Cruchaga, C., Sassi, C., Kauwe, J.S.K., Younkin, S., et al.; Alzheimer Genetic Analysis Group (2013a). TREM2 variants in Alzheimer's disease. N. Engl. J. Med. *368*, 117–127.

Guerreiro, R.J., Lohmann, E., Brás, J.M., Gibbs, J.R., Rohrer, J.D., Gurunlian, N., Dursun, B., Bilgic, B., Hanagasi, H., Gurvit, H., et al. (2013b). Using exome sequencing to reveal mutations in TREM2 presenting as a frontotemporal dementia-like syndrome without bone involvement. JAMA Neurol. *70*, 78–84.

Haenseler, W., Sansom, S.N., Buchrieser, J., Newey, S.E., Moore, C.S., Nicholls, F.J., Chintawar, S., Schnell, C., Antel, J.P., Allen, N.D., et al. (2017). A Highly Efficient Human Pluripotent Stem Cell Microglia Model Displays a Neuronal-Co-culture-Specific Expression Profile and Inflammatory Response. Stem Cell Reports *8*, 1727–1742.

Hale, C., Yeung, A., Goulding, D., Pickard, D., Alasoo, K., Powrie, F., Dougan, G., and Mukhopadhyay, S. (2015). Induced pluripotent stem cell derived macrophages as a cellular system to study salmonella and other pathogens. PLoS One *10*, e0124307.

Hamerman, J.A., Jarjoura, J.R., Humphrey, M.B., Nakamura, M.C., Seaman, W.E., and Lanier, L.L. (2006). Cutting edge: inhibition of TLR and FcR responses in macrophages by triggering receptor expressed on myeloid cells (TREM)-2 and DAP12. J. Immunol. *177*, 2051–2055.

Hickman, S.E., Kingery, N.D., Ohsumi, T.K., Borowsky, M.L., Wang, L.C., Means, T.K., and El Khoury, J. (2013). The microglial sensome revealed by direct RNA sequencing. Nat. Neurosci. *16*, 1896–1905.

Hsieh, C.L., Koike, M., Spusta, S.C., Niemi, E.C., Yenari, M., Nakamura, M.C., and Seaman, W.E. (2009). A role for TREM2 ligands in the phagocytosis of apoptotic neuronal cells by microglia. J. Neurochem. *109*, 1144–1156.

Jay, T.R., Miller, C.M., Cheng, P.J., Graham, L.C., Bemiller, S., Broihier, M.L., Xu, G., Margevicius, D., Karlo, J.C., Sousa, G.L., et al. (2015). TREM2 deficiency eliminates TREM2+ inflammatory macrophages and ameliorates pathology in Alzheimer's disease mouse models. J. Exp. Med. *212*, 287–295.

Jay, T.R., Hirsch, A.M., Broihier, M.L., Miller, C.M., Neilson, L.E., Ransohoff, R.M., Lamb, B.T., and Landreth, G.E. (2017). Disease progression-dependent effects of TREM2 deficiency in a mouse model of Alzheimer's disease. J. Neurosci. *37*, 637–647.

Jonsson, T., Stefansson, H., Steinberg, S., Jonsdottir, I., Jonsson, P.V., Snaedal, J., Bjornsson, S., Huttenlocher, J., Levey, A.I., Lah, J.J., et al. (2013). Variant of TREM2 associated with the risk of Alzheimer's disease. N. Engl. J. Med. *368*, 107–116.

Kawabori, M., Kacimi, R., Kauppinen, T., Calosing, C., Kim, J.Y., Hsieh, C.L., Nakamura, M.C., and Yenari, M.A. (2015). Triggering receptor expressed on myeloid cells 2 (TREM2) deficiency attenuates phagocytic activities of microglia and exacerbates ischemic damage in experimental stroke. J. Neurosci. *35*, 3384–3396.

Kleinberger, G., Yamanishi, Y., Suárez-Calvet, M., Czirr, E., Lohmann, E., Cuyvers, E., Struyfs, H., Pettkus, N., Wenninger-Weinzierl, A., Mazaheri, F., et al. (2014). TREM2 mutations implicated in neurodegeneration impair cell surface transport and phagocytosis. Sci. Transl. Med. *6*, 243ra86.

Kleinberger, G., Capell, A., Brouwers, N., Fellerer, K., Sleegers, K., Cruts, M., Van Broeckhoven, C., and Haass, C. (2016). Reduced secretion and altered proteolytic processing caused by missense mutations in progranulin. Neurobiol. Aging *39*, 220.e17–220.e26.

Matarin, M., Salih, D.A., Yasvoina, M., Cummings, D.M., Guelfi, S., Liu, W., Nahaboo Solim, M.A., Moens, T.G., Paublete, R.M., Ali, S.S., et al. (2015). A genome-wide gene-expression analysis and database in transgenic mice during development of amyloid or tau pathology. Cell Rep. *10*, 633–644.

Mazaheri, F., Snaidero, N., Kleinberger, G., Madore, C., Daria, A., Werner, G., Krasemann, S., Capell, A., Trümbach, D., Wurst, W., et al. (2017). TREM2 deficiency impairs chemotaxis and microglial responses to neuronal injury. EMBO Rep. *18*, 1186–1198.

Muffat, J., Li, Y., Mitalipova, M., Omer, A., Corcoran, S., Bakiasi, G., Tsai, L.-H., Aubourg, P., Ransohoff, R.M., and Jaenisch, R. (2016). Derivation of microglia-like cells from human pluripotent stem cells. Nat. Neurosci. *22*, 1358–1367.

Paloneva, J., Manninen, T., Christman, G., Hovanes, K., Mandelin, J., Adolfsson, R., Bianchin, M., Bird, T., Miranda, R., Salmaggi, A., et al. (2002). Mutations in two genes encoding different subunits of a receptor signaling complex result in an identical disease phenotype. Am. J. Hum. Genet. *71*, 656–662.

Pandya, H., Shen, M.J., Ichikawa, D.M., Sedlock, A.B., Choi, Y., Johnson, K.R., Kim, G., Brown, M.A., Elkahloun, A.G., Maric, D., et al. (2017). Differentiation of human and murine induced pluripotent stem cells to microglia-like cells. Nat. Neurosci. *20*, 753–759.

Piccio, L., Deming, Y., Del-Águila, J.L., Ghezzi, L., Holtzman, D.M., Fagan, A.M., Fenoglio, C., Galimberti, D., Borroni, B., and Cruchaga, C. (2016). Cerebrospinal fluid soluble TREM2 is higher in Alzheimer disease and associated with mutation status. Acta Neuropathol. *131*, 925–933.

Sasaki, A., Kakita, A., Yoshida, K., Konno, T., Ikeuchi, T., Hayashi, S., Matsuo, H., and Shioda, K. (2015). Variable expression of microglial DAP12 and TREM2 genes in Nasu-Hakola disease. Neurogenetics *16*, 265–276.

Sieber, M.W., Jaenisch, N., Brehm, M., Guenther, M., Linnartz-Gerlach, B., Neumann, H., Witte, O.W., and Frahm, C. (2013). Attenuated inflammatory response in triggering receptor expressed on myeloid cells 2 (TREM2) knock-out mice following stroke. PLoS One *8*, e52982.

Smith, A.M., and Dragunow, M. (2014). The human side of microglia. Trends Neurosci. *37*, 125–135.

Takahashi, K., Rochford, C.D.P., and Neumann, H. (2005). Clearance of apoptotic neurons without inflammation by microglial triggering receptor expressed on myeloid cells-2. J. Exp. Med. *201*, 647–657.

Turnbull, I.R., Gilfillan, S., Cella, M., Aoshi, T., Miller, M., Piccio, L., Hernandez, M., and Colonna, M. (2006). Cutting edge: TREM-2 attenuates macrophage activation. J. Immunol. *177*, 3520–3524.

Ulland, T.K., Song, W.M., Huang, S.C.-C., Ulrich, J.D., Sergushichev, A., Beatty, W.L., Loboda, A.A., Zhou, Y., Cairns, N.J., Kambal, A., et al. (2017). TREM2 Maintains Microglial Metabolic Fitness in Alzheimer's Disease. Cell *170*, 649–663.e13.

Underhill, D.M. (2003). Macrophage recognition of zymosan particles. J. Endotoxin Res. *9*, 176–180.

van Wilgenburg, B., Browne, C., Vowles, J., and Cowley, S.A. (2013). Efficient, long term production of monocyte-derived macrophages from human pluripotent stem cells under partly-defined and fully-defined conditions. PLoS One *8*, e71098.

Villegas-Llerena, C., Phillips, A., Garcia-Reitboeck, P., Hardy, J., and Pocock, J.M. (2016). Microglial genes regulating neuroinflammation in the progression of Alzheimer's disease. Curr. Opin. Neurobiol. *36*, 74–81.

Wang, Y., Cella, M., Mallinson, K., Ulrich, J.D., Young, K.L., Robinette, M.L., Gilfillan, S., Krishnan, G.M., Sudhakar, S., Zinselmeyer, B.H., et al. (2015). TREM2 lipid sensing sustains the microglial response in an Alzheimer's disease model. Cell *160*, 1061–1071.

Wolf, S.A., Boddeke, H.W.G.M., and Kettenmann, H. (2017). Microglia in Physiology and Disease. Annu. Rev. Physiol. *79*, 619–643.

Wu, K., Byers, D.E., Jin, X., Agapov, E., Alexander-Brett, J., Patel, A.C., Cella, M., Gilfilan, S., Colonna, M., Kober, D.L., et al. (2015). TREM-2 promotes macrophage survival and lung disease after respiratory viral infection. J. Exp. Med. *212*, 681–697.

Wunderlich, P., Glebov, K., Kemmerling, N., Tien, N.T., Neumann, H., and Walter, J. (2013). Sequential proteolytic processing of the triggering receptor expressed on myeloid cells-2 (TREM2) protein by ectodomain shedding and γ-secretase-dependent intramembranous cleavage. J. Biol. Chem. *288*, 33027–33036.

Xiang, X., Werner, G., Bohrmann, B., Liesz, A., Mazaheri, F., Capell, A., Feederle, R., Knuesel, I., Kleinberger, G., and Haass, C. (2016). TREM2 deficiency reduces the efficacy of immunotherapeutic amyloid clearance. EMBO Mol. Med. *8*, 992–1004.

Zhang, B., Gaiteri, C., Bodea, L.-G., Wang, Z., McElwee, J., Podtelezhnikov, A.A., Zhang, C., Xie, T., Tran, L., Dobrin, R., et al. (2013). Integrated systems approach identifies genetic nodes and networks in late-onset Alzheimer's disease. Cell *153*, 707–720.

Zhong, L., Chen, X.-F., Wang, T., Wang, Z., Liao, C., Wang, Z., Huang, R., Wang, D., Li, X., Wu, L., et al. (2017). Soluble TREM2 induces inflammatory responses and enhances microglial survival. J. Exp. Med. *214*, 597–607.

Cell Reports, Volume *24*

Supplemental Information

Human Induced Pluripotent Stem Cell-Derived Microglia-Like Cells Harboring TREM2 Missense Mutations Show Specific Deficits in Phagocytosis

Pablo Garcia-Reitboeck, Alexandra Phillips, Thomas M. Piers, Claudio Villegas-Llerena, Matt Butler, Anna Mallach, Celia Rodrigues, Charles E. Arber, Amanda Heslegrave, Henrik Zetterberg, Harald Neumann, Stephen Neame, Henry Houlden, John Hardy, and Jennifer M. Pocock

Supplementary Experimental Procedures

Supplementary text

The karyotype of TREM2 mutant iPSC-lines was assessed with CNV analysis. Both clones of T66Mhet2 had a 47, XYY phenotype (Suppl. Fig1A). Both T66Mhom clones had large areas of loss of heterozygosity on chromosome 6 and 7 (suppl. Fig1B). Clone 3 of the T66Mhom iPSC line featured a heterozygous deletion of approximately 7MB on chromosome 3, starting at position 63348134 and ending at 69934331 (suppl. Fig. 1C). Karyotype analysis of T66Mhom clone 3 was normal (suppl. Fig. 1D). The CNV analysis data for all TREM2 mutant lines can be seen in suppl. Table 1.

Generation of human iPSC-derived microglia-like cells

TREM2 mutant primary fibroblast lines were generated from 4mm skin punch biopsies, which were obtained under informed consent. Ethical permission for this study was obtained from the National Hospital for Neurology and Neurosurgery and the Institute of Neurology joint research ethics committee (study reference 09/H0716/64 for collection of W50C mutant fibroblasts) or approved by the Ethics Committee of Istanbul Faculty of Medicine, Istanbul University (for collection of T66M mutant fibroblasts to Dr Ebba Lohmann). iPSC were generated from fibroblast cultures using 4 factor Sendai virus reprogramming and characterised by RT-PCR for plasmid integration, immunofluorescence for expression of pluripotent markers, and 3-germ layer differentiation and at the NIHR Cambridge Biomedical Research Centre (BRC), human Induced Pluripotent Stem Cells core facility (see Brownjohn et al. 2018). Karyotypic integrity was assessed by CNV analysis using OmniExpress-24 chips run on the Illumina Infinum platform. CNVs were checked visually on GenomeStudio software. The following control iPSC lines were used: CTRL1 (kindly provided by Dr Selina Wray, UCL Institute of Neurology), ND (called CTRL2 in this paper, from Coriell Institute of Medical Research Biorepository, kindly provided by Dr Rickie Patani, UCL Institute of Neurology), SBAD03 (called CTRL3, from Stembancc), CTRL4 (SFC840, from Stembancc). iPSC colonies were maintained in E8 medium (Life Technologies), and split every 4-5 days using Versene (Lonza). iPSC colonies were differentiated into iPSC-macrophages within 5 passages. Human iPSC-derived microglia-like cells (iPSC-MGLC) were generated using recently published protocols (van Wilgenburg et al. 2013), with minor modifications. To generate embryoid bodies at day 0 in vitro (D0), iPSC colonies at 60-80% confluence were dissociated for 4 min with TrypLE (Life Technologies), collected in 10 x the volume of PBS without Ca^{2+}/Mg^{2+}, pelleted for 3 min at 300 g and resuspended in 1 ml EBDiff medium (E8 medium with 10 µmol ROCK inhibitor (Cambridge Bioscience), 50 ng/ml BMP-4 (Preprotech), 50 ng/ml VEGF, (Preprotech), 20 ng/ml SCF (Miltenyi)). Cells were counted, resuspended in EBDiff medium at 10^5 cells/ml and 100 µl /well (i.e. 10^4 cells/well) were distributed into low adherence 96 well plates (Corning 7007), centrifuged at 800rpm (115g) for 3 min, and gently

transferred to a tissue culture incubator at 37ºC with 5% CO_2. On D2, 50 µl of EBDiff medium/well were gently added to each well. On D4, embryoid bodies were collected with a P1000 Gilson pipette, transferred into a 15 ml Eppendorf tube, and left to settle at the bottom. The old medium was aspirated and myeloid differentiation medium (X-vivo 15 medium (Lonza) supplemented with 1% Glutamax (Life Technologies), 100U Penicillin/Streptomycin (Life Technologies) and 50 µM mercaptoethanol (Life Technologies), with 100 ng/ml MCSF (Peprotech) and 25 ng/ml IL-3 (Cell Guidance Systems) added. Approximately 150 embryoid bodies were transferred into a 175 cm^2 flask. Myeloid differentiation medium was changed every 5-7 days, making sure that the medium did not become acidotic. After 3-4 weeks, iPSC-MGLC were harvested once a week by replacing 2/3 of the medium and filtering the cells through a 40 micron filter (BD Bioscience). Harvested IPSC-MGLC were further differentiated in X-Vivo 15 medium with 1% Glutamax, 100 U Penicillin/Streptomycin and 100 ng/ml MCSF. iPSC-MGLC were used for experiments between day 2 and day 7 post harvesting.

TREM2 genotyping by Sanger sequencing

Genomic DNA was extracted from approximately 1 million cells using DNeasy Blood & Tissue Kits (QIAGEN), following the manufacturer's instructions. DNA was diluted with nuclease free water to 25ng/µl for TREM2 genotyping. TREM2 genotyping was carried out by PCR and Sanger sequence analysis of the complete second exon of the TREM2 gene. The amplification reaction was performed in a volume of 50µl, which contained 2XKAPA HiFi HotStart ReadyMix (Kapa Biosystems), 0.3µM of each primer (Forward primer: 5'-AGTGGGTGGTTCTGCACAC-3' and Reverse primer: 5'-TCCTTCAGGGCAGGATTTTT-3'), and 50ng genomic DNA. PCR cycling conditions were as follows: Initial denaturation at 95°C for 3 minutes followed by 35 cycles with denaturation at 98°C for 20 sec, annealing at 62°C for 15 sec, extension at 72°C for 30 sec; then a final extension at 72°C for 1 minute. PCR were subsequently visualized on a 1.5% agarose gel to verify the size of the amplicon (533 bp). Finally PCR products were purified using the QIAquick PCR Purification Kit (QIAGEN) and then sequenced commercially (Source Bioscience, Nottingham, UK). Alignment of sequences and analysis was carried out using SnapGene software (GSL Biotech, version 3.3.3).

Isolation of human blood derived-monocytes

Phlebotomy was performed and blood collected into heparinised tubes. This study was approved by University College London (UCL)/UCL Hospitals Joint Research Ethics Committee (Project ID: 7451/001) and all subjects gave informed written consent. Plasma was separated by incubating the heparinized blood with 3% dextran 500 (Sigma) in an upright position for 60 minutes at room temperature. The leukocytes were pelleted from plasma by centrifugation for 6 minutes at

1600 rpm (461g). The cells were resuspended in PBS, layered over Histopaque (Sigma) and centrifuged with low acceleration and brake for 20 minutes at 720g. The resulting peripheral blood mononuclear cell (PBMC) layer was collected and CD14 Microbeads (Miltenyi Biotec) were used to isolate the monocytes, according to the manufacturer's instructions. Blood-derived monocytes were cultured at a density of $5x10^5$ per well in X-Vivo medium (Lonza) supplemented with 100U/ml penicillin/streptomycin (Life Technologies), 1% Glutamax (Life Technologies) and matured into macrophages through continuous culture in 100 ng/ml MCSF (Preprotech) for 6 days.

Quantitative PCR

Cells were lysed and homogenised using RLT buffer (QIAGEN) and total RNA was extracted from cells using an RNeasy minikit (QIAGEN), with an on-column DNase digestion step using RNase-free DNase set (QIAGEN). Complementary DNAs were generated using the High-Capacity RNA-cDNA kit (Life Technologies), according to the manufacturer's instructions. Quantitative PCR were conducted using TaqMan Universal Mastermix II (ThermoFisher) in the Stratagene Mx3000p qPCR system and MxPro qPCR software. Expression was normalised to GAPDH. The following primers were used (all purchased from Applied Biosystems):

**Gene name**	**Primer ID**
APOE	Hs00171168_m1
C1QA	Hs00381122_m1
CD33	Hs01076281_m1
CSF1R	Hs00911250_m1
GAPDH	Hs0278991_g1
GPR34	Hs00758331_m1
PROS1	Hs00165590_m1
SPI1	Hs02786711_m1
P2Y12	Hs00224470_m1
TGFB1	Hs00998133_m1
TMEM119	Hs01938722_u1
TREM2	Hs00219132_m1

TYROBP	Hs00182426_m1

Western blotting

Cellular protein was solubilised using a modified RIPA buffer (25mM TRIS, pH 7.4, 50mM NaCl, 0.5% NP-40, 0.25% Na-Deoxycholate, 1mM EDTA) with protease/phosphatase inhibitors (Life Technologies), and denatured in 4 X sample buffer + 10% Dithiothreitol (DTT). Samples were resolved on sodium dodecyl sulfate (SDS)-polyacrylamide gels, and transferred to 0.2µm nitrocellulose membranes (BioRad). Primary antibodies against goat anti-TREM2 (AF1828, 1:500, R & D Systems; Cell Signaling), rabbit anti-DAP12 (MAB5240, 1:500, R & D Systems), rabbit anti-TREM2 (D8I4C, 1:1000, Cell Signaling Technology) and anti-β-actin (clone AC15, 1:15000, Sigma) were purchased from commercial sources. Immunoreactive bands were probed with goat/donkey anti-rabbit/mouse/goat IgG (H+L) IRDye 680RD or IRDye 800CW (1:10 000, LiCOR) secondary antibodies for 1h and developed using the Odyssey detection system (LiCOR). Optical densities (O.D.) of immunoreactivity were quantified using Odyssey version 3.0 software.

Fluorescence-activated cell sorting

At least $1x10^5$ – $1x10^6$ cells per iPSC line were used for FACS analysis. Cells were harvested by rinsing cells in PBS withouy Ca^{2+}/Mg^{2+} and incubating for 10 minutes in PBS without Ca^{2+}/ Mg^{2+}. Cells were washed 2x with FACS buffer (PBS + 0.5% bovine serum albumin (BSA, Sigma) + 2mM EDTA), incubated with FC-block (Miltenyi) and primary antibodies (1:10) or isotype control antibodies for 1h at 4ºC, washed 3x with FACS buffer and analysed using a BD FACS Calibur analyser. Results were analysed using FlowJo software (v8.8.7, Tree Star). The following antibodies were used for FACS: rat anti-CD11B-APC (ICRF44, Insight Biotechnologies), mouse anti-CD45-FITC (130-080-202, Miltenyi), anti-CSF1R FITC (FAB329F-100, R&D systems), mouse anti-CD33 FITC (130-098-695, Miltenyi).The following isotype antibodies were used: mouse IgG2a-FITC (Miltenyi), mouse IgG1-FITC (130-092-213, Miltenyi), rat IgG2-APC (130-103-085, Miltenyi).

Immunocytochemistry

Cells were seeded at $2.5x10^4$ cells/well, fixed in 4% paraformaldehyde in PBS, washed 1x with PBS, permeabilised with 0.2% Triton X in PBS for 10 minutes at room temperature (RT), blocked in 5% donkey (TREM2 AF1828 antibody) or 5% goat (all other antibodies) serum in PBS for 1h at RT, and incubated in 1% donkey or goat serum in PBS with primary antibody for 2h at RT. For double-immunofluorescence with anti-TREM2 and Golgin antibodies, cells were

blocked with 3% bovine serum albumin (BSA) and incubated with 1% BSA in PBS with primary and secondary antibodies, Cells were washed 3x with PBS and incubated for 1h with Alexa Fluor secondary antibodies in 1% serum PBS. Images were taken using a Leica DM550 microscope and LAS AF software (Leica, V2.6.3). For live cell imaging of pHrodo particles, images were taken using a Zeiss Axioskop 2 microscope and Axiovison software (Zeiss, v4.8). Confocal images were taken with a Zeiss LSM 710 confocal microscope using Zen software (Zeiss, Version 2012), and images processed with Image J1.5 (www.imagej.nih.gov/ij). The following antibodies were used for immunocytochemistry: primary antibodies: mouse anti-CD68 (1:100, DAKO), rabbit anti-iba1 (1:500, WAKO), goat anti-TREM2 AF1828 (10µg/ml, R&D systems), rabbit-anti-TREM2 (D814C, 1:400, Cell Signaling Technology), rabbit-anti-Golgin (1:100, Life Technologies). The following secondary antibodies were used: Alexa Fluor donkey anti-goat 568, goat-anti-rabbit 568, goat-anti mouse 488 (all 1:500, Life Technologies).

sTREM2 ELISA

Quantification of sTREM2 from cell culture supernatants was performed using an in house-generated ELISA. MaxiSORP 96 well plates (Nunc) were coated with 1µg/ml of a rat anti-mouse/human monoclonal antibody (R&D Systems; clone 237920) overnight at 4°C. Plates were washed with 1x PBS/0.1% Tween-20 (PBS-T) then blocked with 1% bovine serum albumin (BSA) in 0.1% Tween 20 in PBS (1% BSA/PBS-T; pH 7.4) for 45 minutes at room temperature (RT) with gentle shaking, followed by 3 washes with PBS-T. Cell culture supernatant samples and standards (Recombinant human TREM2-His; Life Technologies; 0 - 20ng/ml in 1% BSA/PBS-T) were then incubated for 2h at RT with gentle shaking, followed by 3 washes with PBS-T. For the detection of bound human sTREM2 from the cell culture supernatants, plates were incubated for 1.5h at RT with biotinylated polyclonal goat anti-human TREM2 capture antibody (0.1µg/ml; R&D Systems) diluted in 1% BSA/PBS-T. Plates were subsequently washed 4 times with PBS-T, followed by incubation with a streptavidin-HRP (Invitrogen; 0.1µg/ml) diluted in PBS-T for 45 minutes at RT with gentle shaking. Plates were washed 3 times with PBS-T followed by the addition of a chromogenic substrate solution (TMB) in the dark. The reaction was terminated with the addition of stop solution (0.16M H_2SO_4) and absorbance was read at 450 nm (Tecan Genios). To further verify sTREM2 secretion a second method was employed, adapted from Kleinberger et al., (2014). Streptavidin-coated 96-well plates (Meso-Scale discovery (MSD)) were blocked overnight at 4°C in block buffer (0.5% bovine serum albumin (BSA) and 0.05% Tween 20 in PBS (pH 7.4). The plates were next incubated with the biotinylated polyclonal goat anti-human TREM2 capture antibody (0.25µg/ml R&D Systems BAF1828) diluted in block buffer for 1h at room temperature. They were subsequently washed four times with wash buffer (0.05% Tween 20 in PBS) and incubated for 2h at RT with the undiluted cell culture media samples or a standard

curve constructed from recombinant human TREM2 protein (4000 -62.5pg/ml Sino Biological Inc 11084-H08H) diluted in assay buffer (0.25% BSA and 0.05% Tween 20 in PBS (pH=7.4). Plates were again washed three times with wash buffer before incubation for 1h at RT with the detector antibody monoclonal mouse anti-human TREM2 antibody (1µg/ml Santa Cruz Biotechnology; B-3, sc373828). After three additional washing steps, plates were incubated with the secondary antibody (SULFO-TAG–labeled anti-mouse secondary antibody, MSD) and incubated for 1h in the dark. Lastly, plates were washed three times with wash buffer followed by two washing steps in PBS alone. The electrochemical signal was developed by adding MSD Read buffer (1 in 2) and the light emission measured using the MSD SECTOR Imager 6000. The concentration of sTREM2 was calculated using a five-parameter logistic curve fitting method with the MSD Workbench software package. Intra-assay cvs were < 10%, and all samples were measured on the same day using the same reagents.

pHrodo *E.coli* and pHrodo zymosan phagocytosis assay

iPSC-MGLC were seeded at 10^5 cells /well in 24 well plates (Corning) 48h before the assay. As a negative control, cells were preincubated for 30 minutes with 10µM Cytochalasin D (Sigma). pHrodo green *E.coli* particles (Life Technologies) were diluted in PBS without Ca^{2+}/Mg^{2+} to a concentration of 1mg/ml, and 50µg were added per well in 200µl of macrophage end-differentiation medium. PHrodo zymosan particles (Life Technologies) were diluted in PBS without Ca^{2+}/Mg^{2+} to a concentration of 0.5mg/ml, and 25µg were added per well in 200µl of macrophage end-differentiation medium. Cells were incubated with pHrodo *E.coli* or pHrodo zymosan particles for 2h, rinsed and harvested with PBS without Ca^{2+}/Mg^{2+} and washed twice with FACS buffer (PBS + 0.1% BSA, 2mM EDTA, 0.05% sodium azide). Cells were analysed with a BD FACS Calibur flow cytometer and results analysed with Flowing software (Cell Imaging Core of the Turku Centre for Biotechnology, flowingsoftware.btk.fi). Mean fluorescence intensities were calculated for each sample from 3 independent experiments, normalised to one control line and pooled for statistical analysis.

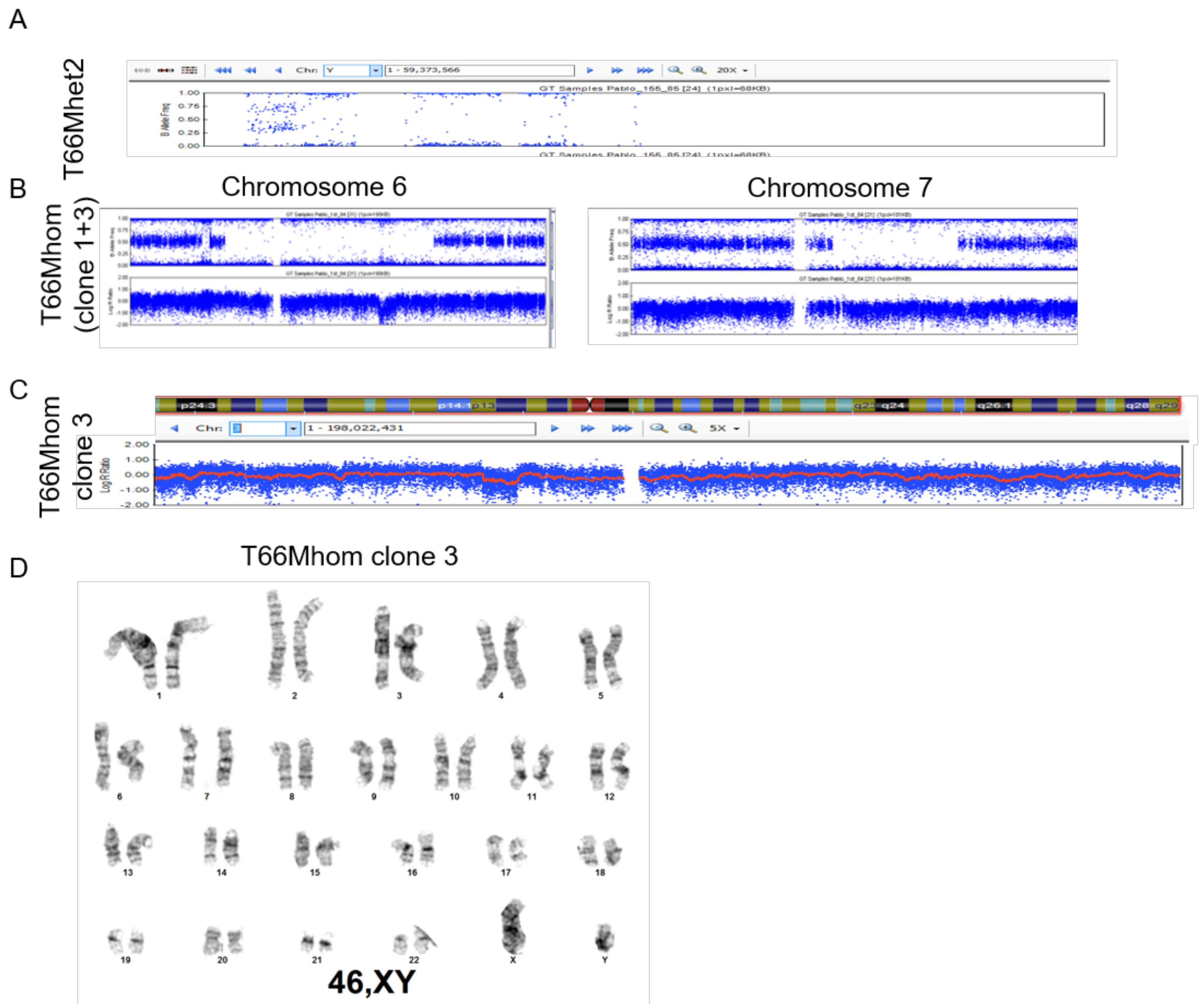

Figure S1. Karyotyping and CNV analysis of TREM2 mutant iPSC. Related to Figure 2.

A 47, XYY karyotype was identified for both clones of T66Mhet2 iPSC line (A, analysis of one clone shown). Areas of prominent loss of heterozygosity were identified on chromosome 6 and 7 for clones of the T66Mhom line (B). The T66Mhom clone 3 iPSC line featured a heterozygous deletion of approximately 7MB on chromosome 3, starting at position 63348134 and ending at 69934331 (C). Karyotype analysis of T66Mhom was normal (D).

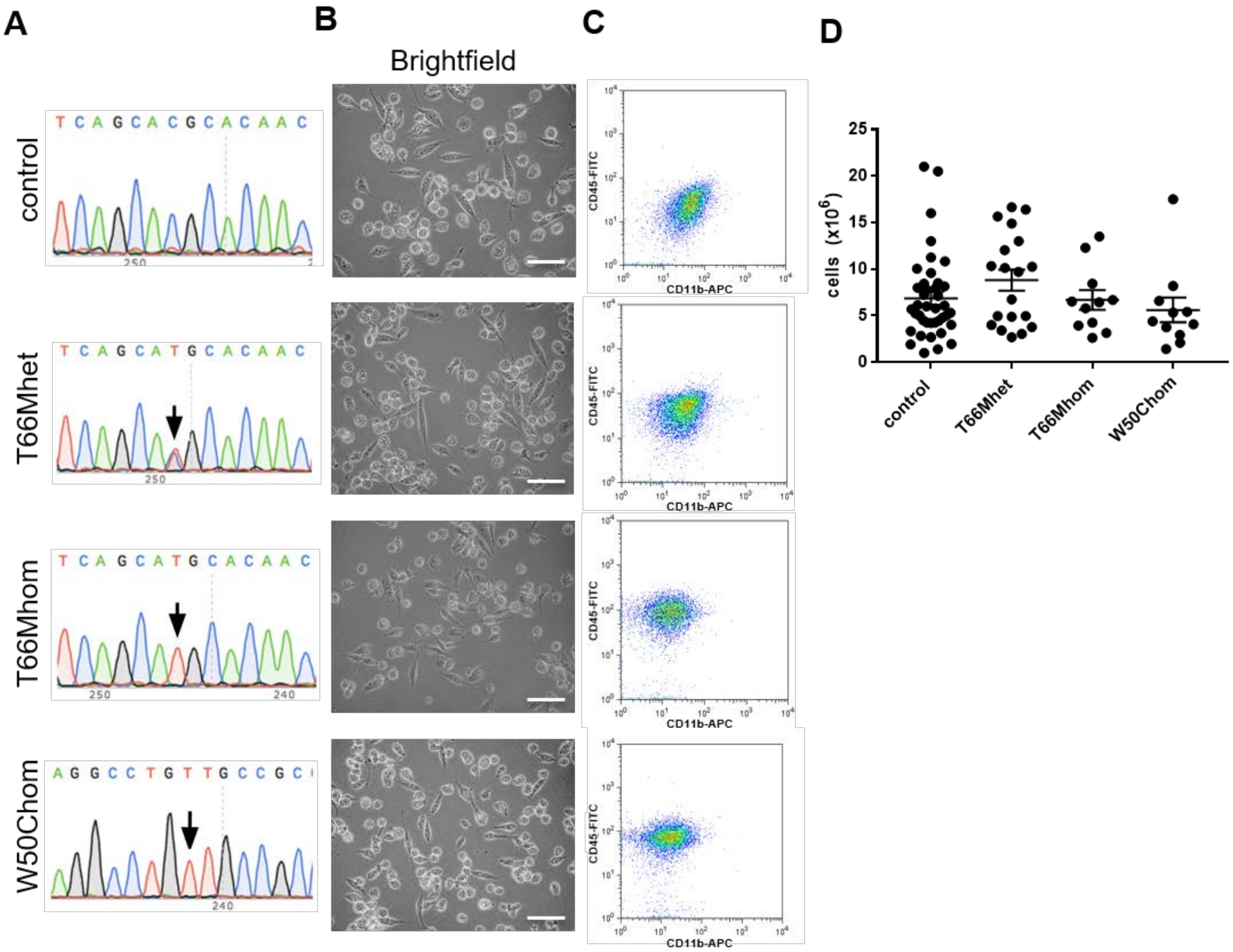

Figure S2. Genotype characterisation. Related to Figures 1 and 2.

Sanger sequencing of T66M heterozygous, T66M and W50C homozygous mutations in TREM2 variant iPSC-MGLC (arrows, A, one representative control and T66Mhet iPSC-MGLC shown). Brightfield microscopy of control and TREM2 variant iPSC-MGLC, scale bar=100 µm (B). FACS analysis of the macrophage markers CD45 and CD11b in control and TREM2 variant iPSC-MGLC (C). The weekly iPSC-MGLC yield between control and TREM2 variant iPSC (D). One representative control and T66Mhet line are shown in Figures 2A-C. Data are represented as mean ± SEM.

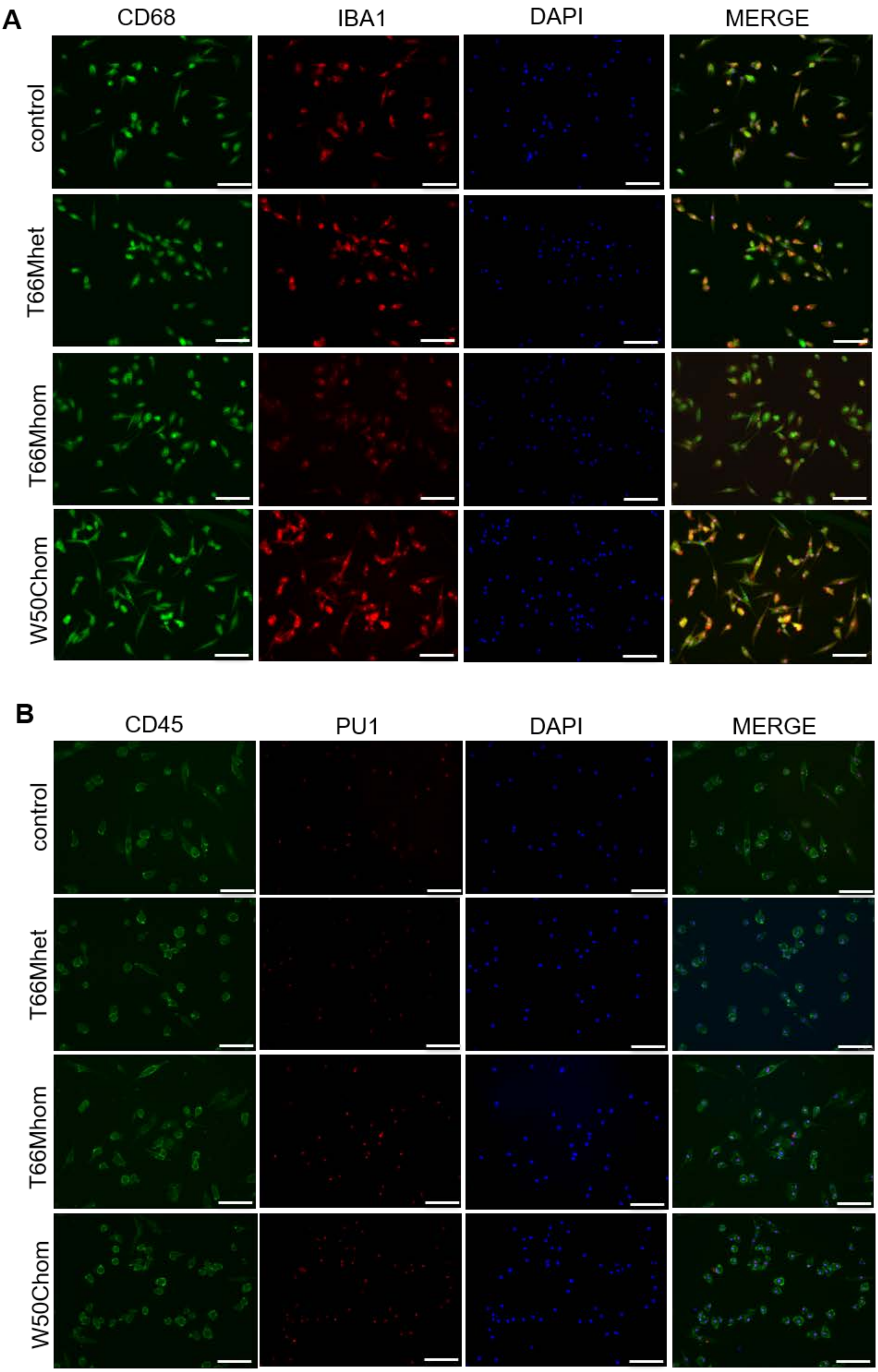
A
CD68
IBA1
DAPI
MERGE
control
T66Mhet
T66Mhom
W50Chom
B
CD45
PU1
DAPI
MERGE
control
T66Mhet
T66Mhom
W50Chom

Figure S3. Expression of macrophage markers in control and TREM2 variant iPSC-MGLC. Related to Figures 1 and 2.

Double-immunofluorescence staining for the macrophage markers CD68 and Iba1 (A) and CD45 and Pu1 (B) in control, T66Mhet, T66Mhom, and W50Chom iPSC-MGLC. Scale bar: 100µm

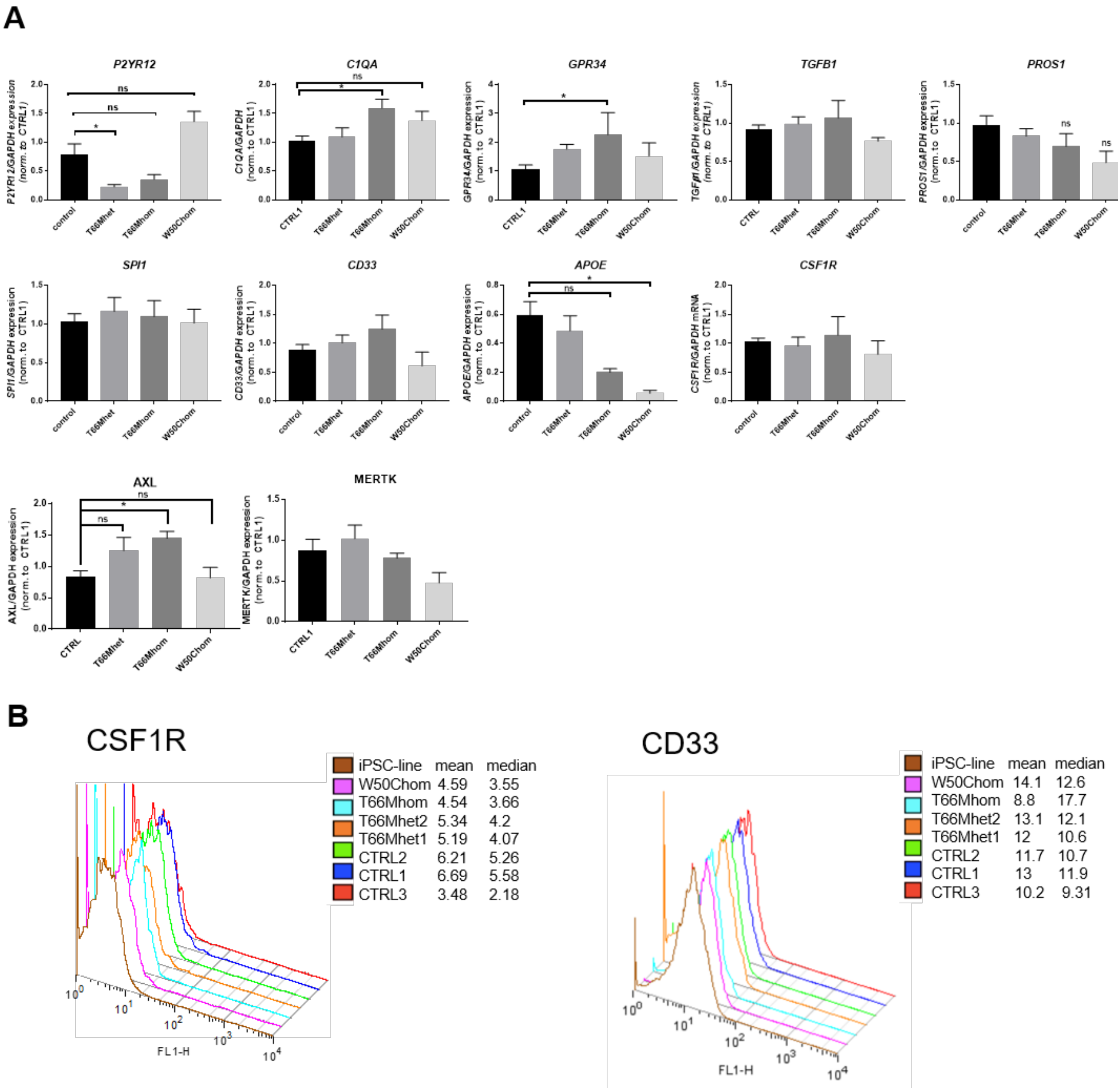

Figure S4. Microglial gene and protein signature analysis. Related to Figures 1 and 2.

Comparison of microglial gene expression in control and TREM2 variant iPSC-MGLC (A). (* $p<0.05$). FACS analysis of surface expression of CSF1R or CD33 (B) in TREM2 variant iPSC-MGLC compared with controls; n=3, the table displays mean and median fluorescent intensities.

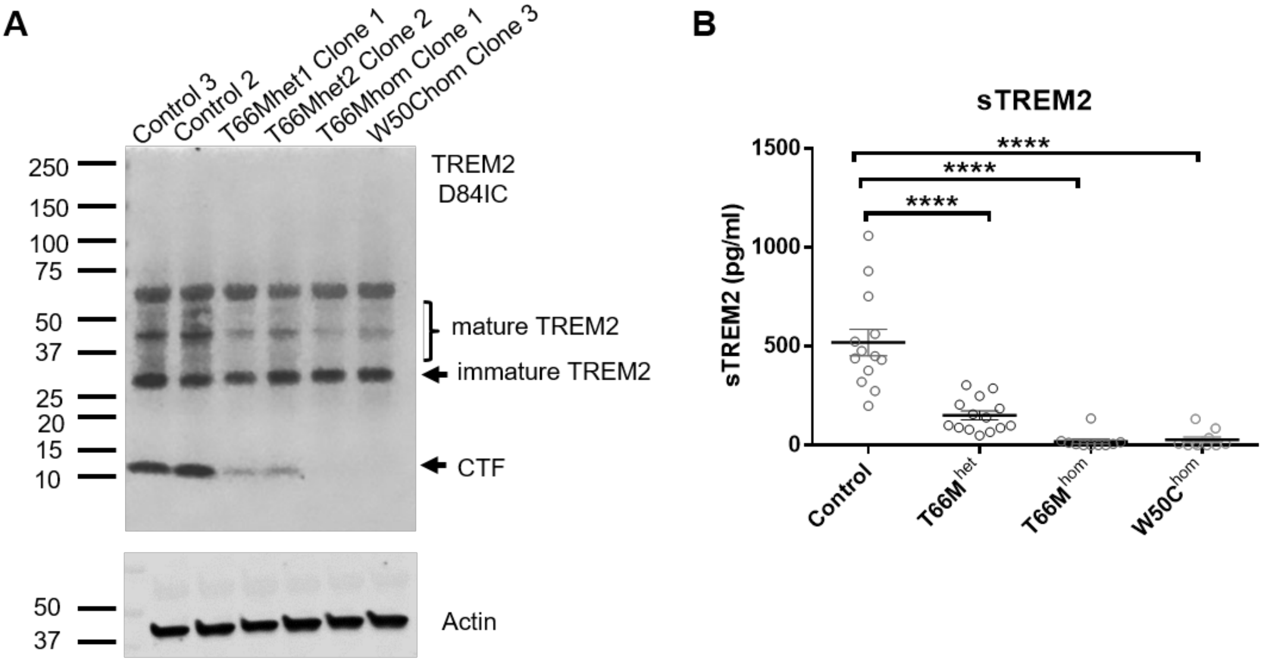

Figure S5. Protein analysis of additional clones. Related to Figure 2.

WB using D8I4C antibody against TREM2 on additional TREM2 variant iPSC clones (1 additional clones per TREM2 variant line) (A). sTREM2 ELISA of additional TREM2 variant iPSC clones (1 additional clone per TREM2 mutant line) (B, **** p<0.0001).

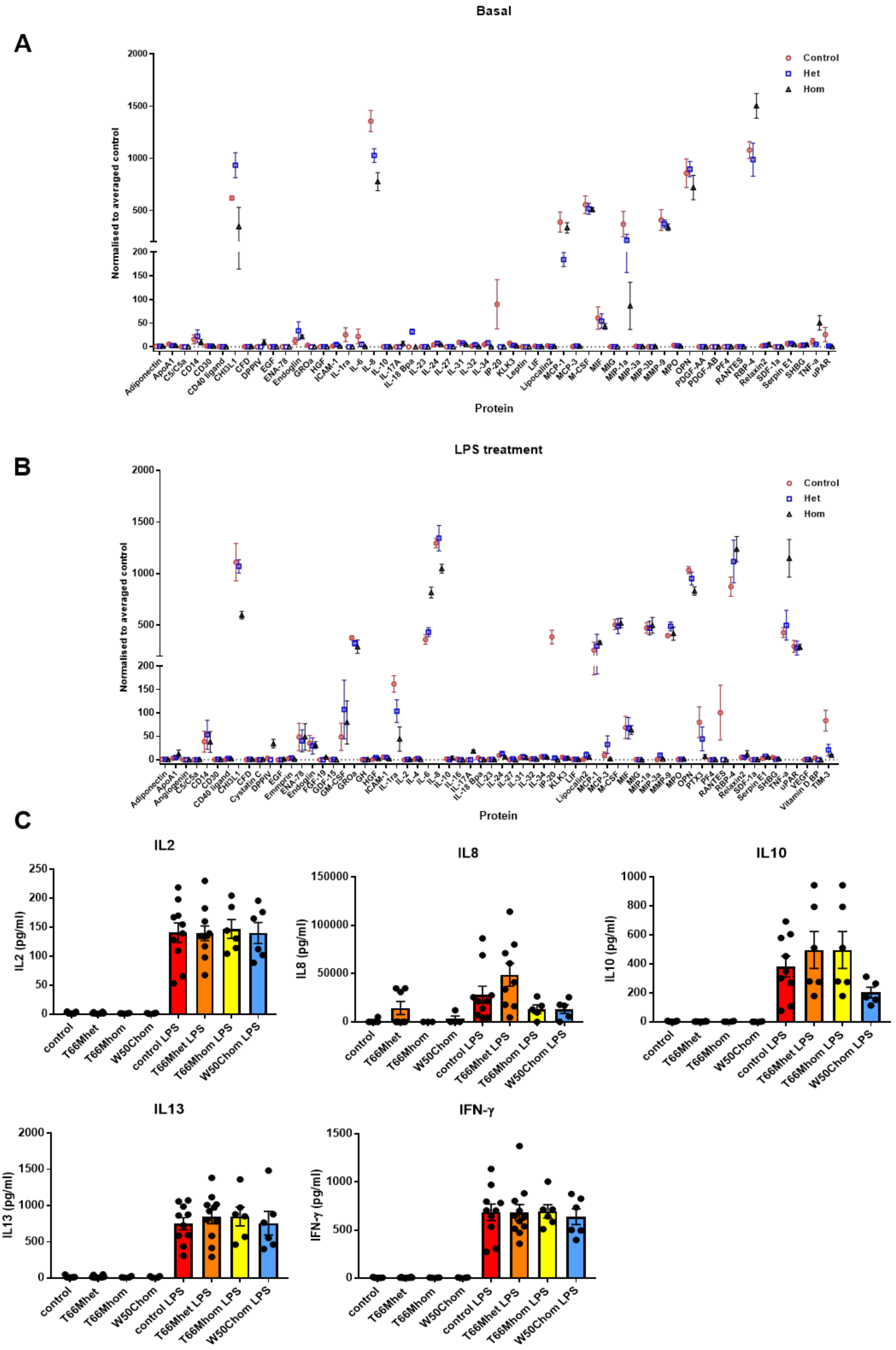

A
Basal
Normalised to averaged control
Protein
Control
Het
Hom
Adiponectin
ApoA1
C5/C5a
CD14
CD30
CD40 ligand
CHI3L1
CFD
DPPIV
EGF
ENA-78
Endoglin
GROa
HGF
ICAM-1
IL-1ra
IL-6
IL-8
IL-10
IL-17A
IL-18 Bpa
IL-23
IL-24
IL-27
IL-31
IL-32
IL-34
IP-20
KLK3
Leptin
LIF
Lipocalin2
MCP-1
MCP-3
M-CSF
MIF
MIG
MIP-1a
MIP-3a
MIP-3b
MMP-9
MPO
OPN
PDGF-AA
PDGF-AB
PF4
RANTES
RBP-4
Relaxin2
SDF-1a
Serpin E1
SHBG
TNF-a
uPAR
B
LPS treatment
Normalised to averaged control
Protein
Control
Het
Hom
Adiponectin
ApoA1
Angiogenin
C5/C5a
CD14
CD30
CD40 ligand
CHI3L1
CFD
Cystatin C
DPPIV
EGF
Emmprin
ENA-78
Endoglin
FGF-19
GDF-15
GM-CSF
GROa
GH
HGF
ICAM-1
IL-1a
IL-2
IL-4
IL-6
IL-8
IL-10
IL-16
IL-17A
IL-18 Bpa
IL-23
IL-24
IL-27
IL-31
IL-32
IL-34
IP-20
KLK3
LIF
Lipocalin2
MCP-1
MCP-3
M-CSF
MIF
MIG
MIP-1a
MIP-3a
MMP-9
MPO
OPN
PTX3
PF4
RANTES
RBP-4
Relaxin2
SDF-1a
Serpin E1
SHBG
TNF-a
uPAR
VEGF
Vitamin DBP
TIM-3
C
IL2
IL2 (pg/ml)
IL8
IL8 (pg/ml)
IL10
IL10 (pg/ml)
IL13
IL13 (pg/ml)
IFN-γ
IFN-γ (pg/ml)
control
T66Mhet
T66Mhom
W50Chom
control LPS
T66Mhet LPS
T66Mhom LPS
W50Chom LPS

Figure S6. Complete proteome profiler analysis and other MSD cytokine profiles. Related to Figure 4.

Complete cytokine profiles using Human XL cytokine array on cell culture supernatants from one control line, one T66Mhet line, one T66Mhom and W50Chom iPSC-MGLC under basal (A; untreated) conditions and following stimulation with LPS, 100 ng/ml after 18 h (B). Levels of remaining tested cytokines: IL2, IL8, IL10, IL13, and IFNγ measured on the MSD platform using a V-PLEX Pro-inflammatory Panel 1 (C). (n=2 for untreated, n=3 for LPS-treated samples in 4 control iPSC lines, and 2 clones per TREM2 variant IPSC-lines were analysed).

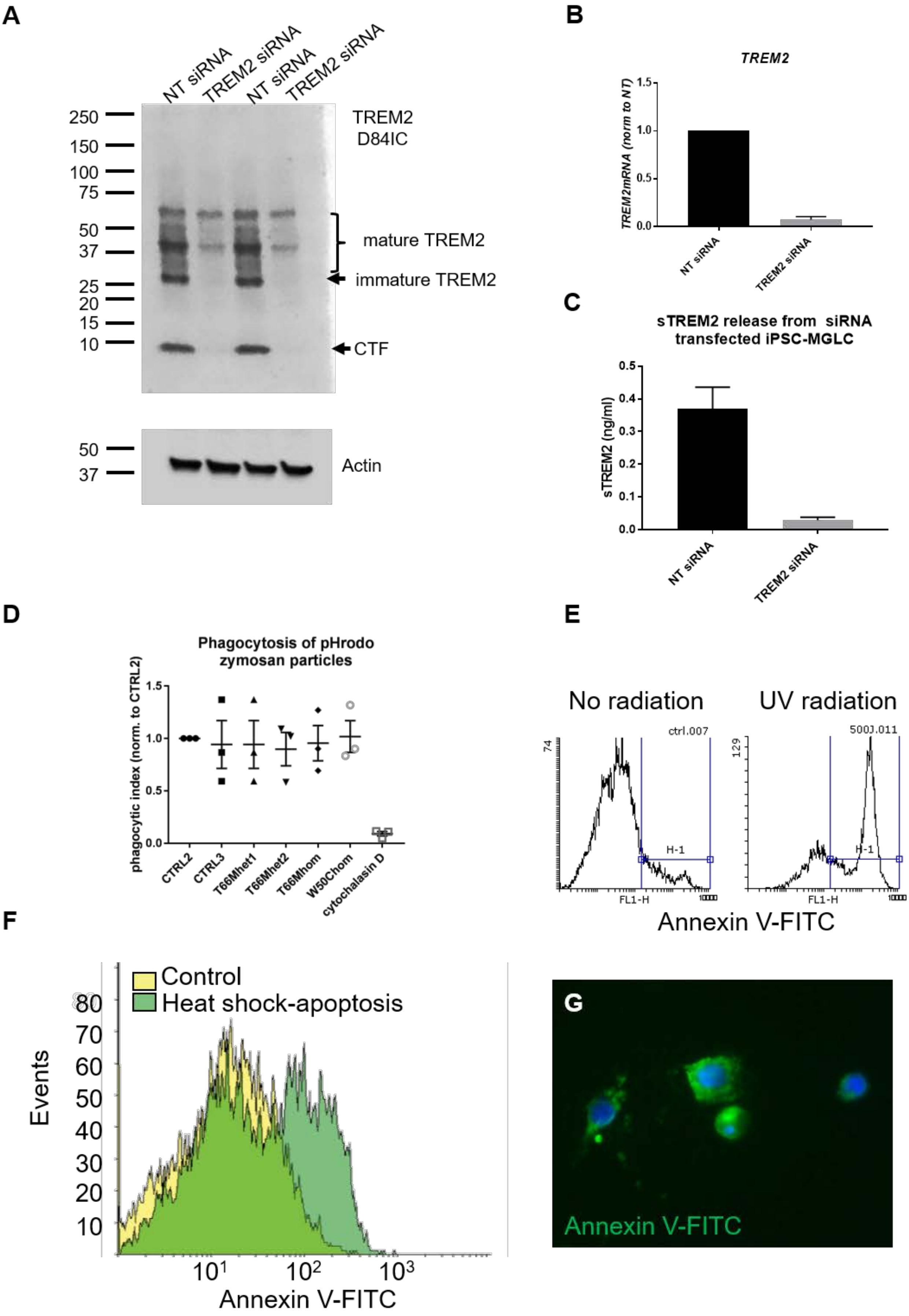
A
NT siRNA
TREM2 siRNA
NT siRNA
TREM2 siRNA
250
150
100
75
50
37
25
20
15
10
TREM2
D84IC
mature TREM2
immature TREM2
CTF
50
37
Actin
B
TREM2
TREM2mRNA (norm to NT)
1.5
1.0
0.5
0.0
NT siRNA
TREM2 siRNA
C
sTREM2 release from siRNA transfected iPSC-MGLC
sTREM2 (ng/ml)
0.5
0.4
0.3
0.2
0.1
0.0
NT siRNA
TREM2 siRNA
D
Phagocytosis of pHrodo zymosan particles
phagocytic index (norm. to CTRL2)
1.5
1.0
0.5
0.0
CTRL2
CTRL3
T66Mhet1
T66Mhet2
T66Mhom
W50Chom
cytochalasin D
E
No radiation
UV radiation
ctrl.007
74
H-1
FL1-H
500J.011
129
Annexin V-FITC
F
Control
Heat shock-apoptosis
Events
80
70
60
50
40
30
20
10
10^1
10^2
10^3
Annexin V-FITC
G
Annexin V-FITC

Figure S7. Characterisation of siRNA knockdown, pHrodo zymosan phagocytosis, and generation of apoptotic SH-SY5Y cells. Related to Figures 5 and 6.

Reduction of TREM2 protein (A), TREM2 mRNA (B), and TREM2 secretion (C) using TREM2 siRNA compared with non-targeting siRNA confirmed by WB, qPCR, or sTREM2 ELISA, respectively (n=3 for qPCR, n=2 for WB and sTREM2 ELISA). Phagocytosis of pHrodo zymosan particles in variant *TREM2* iPSC-MGLC and controls (D, n=3). Cytochalasin D was used as negative control. Data are presented as mean ± SEM. Fluorescent Annexin V staining to demonstrate apoptosis of SH-SY5Y cells following UV radiation and further incubation over a 24h period, compared with un-treated cells (E). FACS analysis for Annexin V staining in SH-SY5Y controls (non-heat shocked, yellow filled graph) versus heat-shock treated (green filled graph) (F). Annexin V immunocytochemistry in heat-shocked SH-SY5Y cells imaged by fluorescence microscopy (G).
